# Increased DNA methylation contributes to the early ripening of pear fruits during domestication and improvement

**DOI:** 10.1186/s13059-024-03220-y

**Published:** 2024-04-05

**Authors:** Bobo Song, Jinshan Yu, Xiaolong Li, Jiaming Li, Jing Fan, Hainan Liu, Weilin Wei, Lingchao Zhang, Kaidi Gu, Dongliang Liu, Kejiao Zhao, Jun Wu

**Affiliations:** 1 College of Horticulture, State Key Laboratory of Crop Genetics & Germplasm Enhancement and Utilization, Nanjing Agricultural University, Nanjing, 210095 Jiangsu China; 2 Zhongshan Biological Breeding Laboratory, Nanjing, 210014 Jiangsu China; 3 Key Laboratory of Quality and Safety Control for Subtropical Fruit and Vegetable, Ministry of Agriculture and Rural Affairs, College of Horticulture Science, Zhejiang A&F University, Hangzhou, 311300 Zhejiang China; 4 Institute of Fruit and Tea, Hubei Academy of Agricultural Sciences, Wuhan, 430072 China; 5 College of Horticulture and Plant Protection, Henan University of Science and Technology, Luoyang, 471023 China; 6 College of Horticulture Science and Engineering, Shandong Agricultural University, Tai’an, Shandong 271018 China

**Keywords:** DNA methylation, Domestication and improvement, Pear, Early ripening

## Abstract

**Background:**

DNA methylation is an essential epigenetic modification. However, its contribution to trait changes and diversity in the domestication of perennial fruit trees remains unknown.

**Results:**

Here, we investigate the variation in DNA methylation during pear domestication and improvement using whole-genome bisulfite sequencing in 41 pear accessions. Contrary to the significant decrease during rice domestication, we detect a global increase in DNA methylation during pear domestication and improvement. We find this specific increase in pear is significantly correlated with the downregulation of *Demeter-like1* (*DML1*, encoding DNA demethylase) due to human selection. We identify a total of 5591 differentially methylated regions (DMRs). Methylation in the CG and CHG contexts undergoes co-evolution during pear domestication and improvement. DMRs have higher genetic diversity than selection sweep regions, especially in the introns. Approximately 97% of DMRs are not associated with any SNPs, and these DMRs are associated with starch and sucrose metabolism and phenylpropanoid biosynthesis. We also perform correlation analysis between DNA methylation and gene expression. We find genes close to the hypermethylated DMRs that are significantly associated with fruit ripening. We further verify the function of a hyper-DMR-associated gene, *CAMTA2*, and demonstrate that overexpression of *CAMTA2* in tomato and pear callus inhibits fruit ripening.

**Conclusions:**

Our study describes a specific pattern of DNA methylation in the domestication and improvement of a perennial pear tree and suggests that increased DNA methylation plays an essential role in the early ripening of pear fruits.

**Supplementary Information:**

The online version contains supplementary material available at 10.1186/s13059-024-03220-y.

## Background

DNA methylation is one of the most important heritable epigenetic modifications that play critical roles in multiple processes, including genome stability, the regulation of gene expression, and gene imprinting [1–7]. In plants, cytosine DNA methylation occurs in three different contexts: CG, CHG, and CHG (H represents A, T, or G) [8, 9]. Different pathways maintain different methylation contexts. CG methylation is maintained by DNA METHYLTRANSFERASE 1 (MET1), CHG methylation is maintained by CHROMOMETHYLASE 3 (CMT3), and CHH methylation is maintained by the CHROMOMETHYLASE 2 (CMT2) and RNA-directed DNA methylation (RdDM) pathways [10–15]. In addition, the level of DNA methylation is regulated by four demethylases, including REPRESSOR OF SILENCING 1/DEMETER-LIKE1 (ROS1/DML1), DEMETER (DME), DEMETER-LIKE 2 (DML2), and DEMETER-LIKE 3 (DML3) [16, 17].

Pears (*Pyrus* ssp., subfamily Amygdaloideae in the family Rosaceae) are among the most important temperate fruit crops worldwide [18]. Pears originated in southwest China and were independently domesticated in Asia and Europe [19]. Compared to wild pears, the fruits of cultivated pears display significant changes in many morphological characteristics, including fruit size, sugar content, and stone cell content. In addition, early ripening, dwarf stature, and good disease resistance are also important targets in pear breeding programs [20]. A comparative analysis between wild pears and cultivated pears can provide insight into the evolution of critical phenotypic changes. To date, resequencing and transcriptomic analyses have provided insight into the genetic basis of pear domestication. Comparative resequencing analysis of 113 pear accessions revealed that Asian and European pears were domesticated independently, and identified some selective sweeps associated with fruit traits such as flesh texture, sugar, acidity, aroma, and stone cell content [19]. A comparative transcriptomic analysis of 41 pear accessions reported the influence of gene expression variation in the distinct fruit trait phenotypes between wild accessions, landraces, and improved pear varieties and identified an important set of differentially expressed genes (DEGs) associated with stone cell content, sugar content, and fruit size [21]. DNA methylation is a crucial heritable epigenetic marker that changes the accessibility of genomic regions and can suppress or activate gene expression, which eventually leads to phenotypic changes [22]. However, the importance of epialleles in domesticating perennial fruit trees has yet to be discovered.

This study obtained single-base-resolution methylomes of 41 accessions of the Asian pear, *Pyrus pyrifolia*, including wild collections, landraces, and improved pears. Comparative methylomic analysis showed that a global increase in DNA methylation occurred during pear domestication and improvement and that this correlates with decreased expression levels of genes encoding DNA demethylases. We identified differentially methylated regions (DMRs) that resulted from the domestication and improvement of pear. The genes near the hyper-DMRs were significantly associated with plant senescence and fruit ripening. Our study provides novel insight into the vital role of methylation variation at maturity during the pear domestication and improvement processes.

## Results

### Human selection on Demeter-like 1 (DML1) may have involved increases in DNA methylation during pear domestication and improvement

To dissect the genome-wide DNA methylation variation that occurred during pear domestication and improvement, we constructed bisulfite sequencing (BS-Seq) libraries with two biological replicates from 41 representative *P. pyrifolia* accessions that included 14 wild, 12 landrace, and 15 improved pear accessions (Additional file 1: Table S1). The average BS conversion rate was 99.44% (Additional file 1: Table S2). The *P. pyrifolia* “Cuiguan” genome was used as the reference genome. To eliminate putative bias that could arise from mapping reads to a single reference genome, we constructed pseudo-references for each accession using resequencing data (Additional file 2: Fig. S1). Compared to the “Cuiguan” reference, the mapping rates were enhanced using pseudo-references for each accession (Additional file 2: Fig. S2). After removing low-confidence cytosine sites (read support ≤ 3), an average of ~ 41 Mb of methylated cytosine sites were identified in each accession, accounting for approximately 40% of the cytosine sites present in the pear genome (Additional file 1: Table S3).

**Fig. 1:**
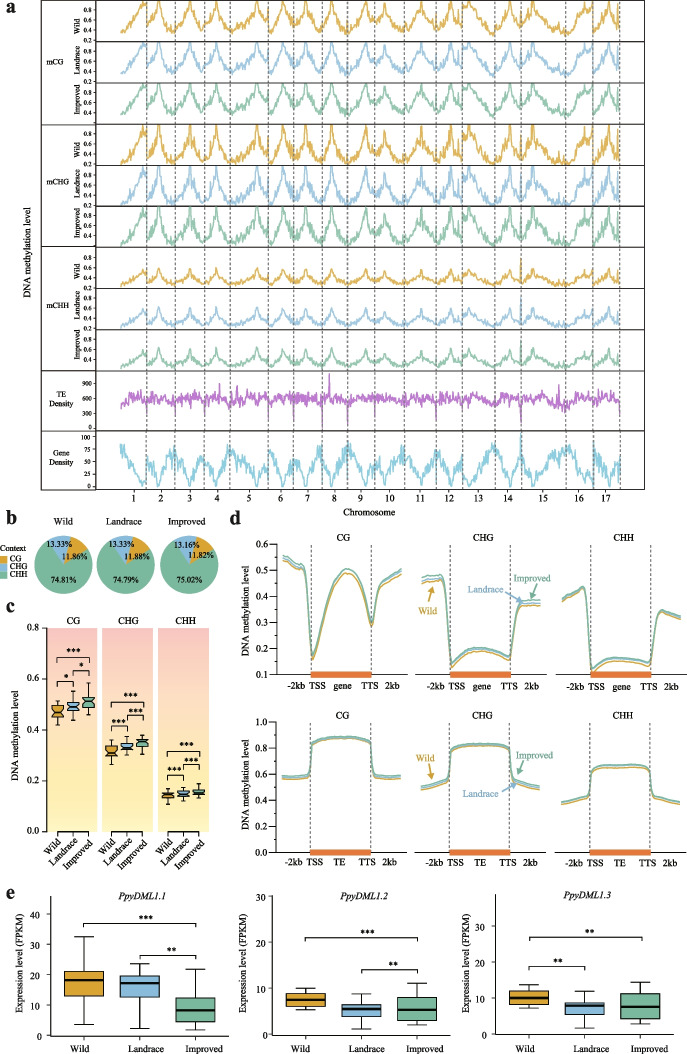
The distribution patterns of DNA cytosine methylation levels in the pear genome. **a** The density distribution of DNA methylation levels in three contexts (CG, CHG, and CHH), gene density, and TE density on the 17 pear chromosomes. **b** The average rates of methylated cytosines (mC) in the three contexts in the pear genome for the three pear populations (wild, landrace, and improved). **c** Comparisons of methylation levels of the three contexts in the wild, landrace, and improved populations (*P < 0.05; **P < 0.01; ***P < 0.001, two-tailed paired Student’s *t*-test). **d** Distribution of DNA methylation levels across the upstream 2 kb, gene body, and downstream 2-kb regions of genes and TEs. **e** Comparisons of relative gene expression levels (FPKM; fragments per kilobase of transcript per million mapped reads) of *PpyDML1.1*, *PpyDML1.2*, and *PpyDML1.3* in the wild, landrace, and improved pear populations. *PpyDML1.1*, *PpyDML1.2*, and *PpyDML1.3* showed continuous decreases in expression during pear domestication and improvement (*P < 0.05; **P < 0.01; ***P < 0.001, differentially expressed analysis using cuffdiff)

We found that the three types of methylated cytosines exhibited similar distribution patterns: more methylated cytosines in the CG, CHG, and CHH contexts were enriched when located in the pericentromeric regions compared to the ends of chromosomes (Fig. 1a). Pearson correlation analysis between DNA methylation and TE/gene density indicated that DNA methylation was positively correlated with TE distribution. In contrast, the correlation with gene distribution was negative (Additional file 2: Fig. S3). We further investigated the distribution of DNA methylation levels across the upstream 2 kb, gene body, and downstream 2-kb regions of different TEs. The result showed that all type TEs exhibited higher methylation levels compared to genes, the TE body in particular (Additional file 2: Fig. S4), which is consistent with previous reports that DNA methylation plays a vital role in preventing their transposition [5, 23].

We calculated the percentage of DNA methylation contexts and found that the CHH context (~ 75%) was the most frequently methylated in the pear genome. Our findings are similar to previously published results for *Prunus mume* (CHH, 75%). Still, they are much higher than for the CHH context methylation in the soybean genome (~ 20%) and in cassava flowers (~ 28%) (Fig. 1b).

We next compared the whole-genome methylation levels in wild pears, landraces, and improved populations. We found that a continuous increase in DNA methylation occurred from pear domestication to pear improvement (Fig. 1c and Additional file 2: Fig. S5). From the distribution of methylated cytosines in the body regions and flanking regions of genes and TEs, we also found that the DNA methylation levels increased in the 5′-3′ regions of genes and TEs during pear domestication and improvement (Fig. 1d). In addition, CG context methylation increased in the body, upstream 2 kb, and downstream 2-kb regions of genes, but not in the transcription start sites (TSS) and transcription termination sites (TTS). CHG and CHH context methylation was enriched into upstream 2 kb and downstream 2-kb sequences of genes, while lacking a gene body region. For all three methylation contexts, the gene body regions had much higher DNA methylation levels compared to the upstream 2 kb and downstream 2-kb sequences in TEs (Fig. 1d).

DNA methylation levels are dynamically regulated by DNA methyltransferase and demethylase enzymes. Therefore, the increases in the levels of DNA methylation in the pear genome during pear domestication and improvement may have resulted from the downregulated expression of DNA demethylase genes or the upregulated expression of DNA methyltransferase genes. To verify this possibility, we investigated the expression patterns of DNA demethylase and methyltransferase genes in all 41 pear accessions. First, we identified a total of 14 DNA methyltransferase genes and 12 DNA demethylase genes (*E*-value < 1E−10). We then chose 26 genes associated with DNA methylation for subsequent analyses based on the phylogenetic tree (Additional file 2: Fig. S6). We next determined the expression levels of these 26 genes in 3 different pear populations. The results did not show a continuous increase or decrease in the relative expression of methyltransferase genes during pear domestication and improvement. Interestingly, we found that the expression of *PpyDML1.1*, *PpyDML1.2*, and *PpyDML1.3* continuously decreased during pear domestication and improvement (Fig. 1e, Additional file 2: Figs. S7 and S8). An analysis of selective sweeps indicated that *PpyDML1.1*, *PpyDML1.2*, and *PpyDML1.3* had undergone human selection during pear domestication and improvement (Additional file 2: Fig. S9). These results show that the *DML1* genes might have been involved in regulating DNA methylation levels during pear domestication and improvement.

Based on the methylation level of each cytosine (mC reads/total reads), we divided them into ten windows and estimated the methylation level in the CG, CHG, and CHH contexts. Most of the CG and CHG context methylated cytosines mapped to a 90–100% window, while most CHH context methylated cytosines were located within a 10–20% window (Additional file 2: Fig. S10). This result indicated that the distribution is highly skewed towards the unmethylated status for the CHH sites. This phenomenon was also found in other plant species, maize, and tomato [24, 25], and the CHH context was removed in previous studies due to low methylation levels. Therefore, we excluded CHH context methylation from further analyses.

### The co-evolution of CG and CHG methylation during pear domestication and improvement

**Fig. 2:**
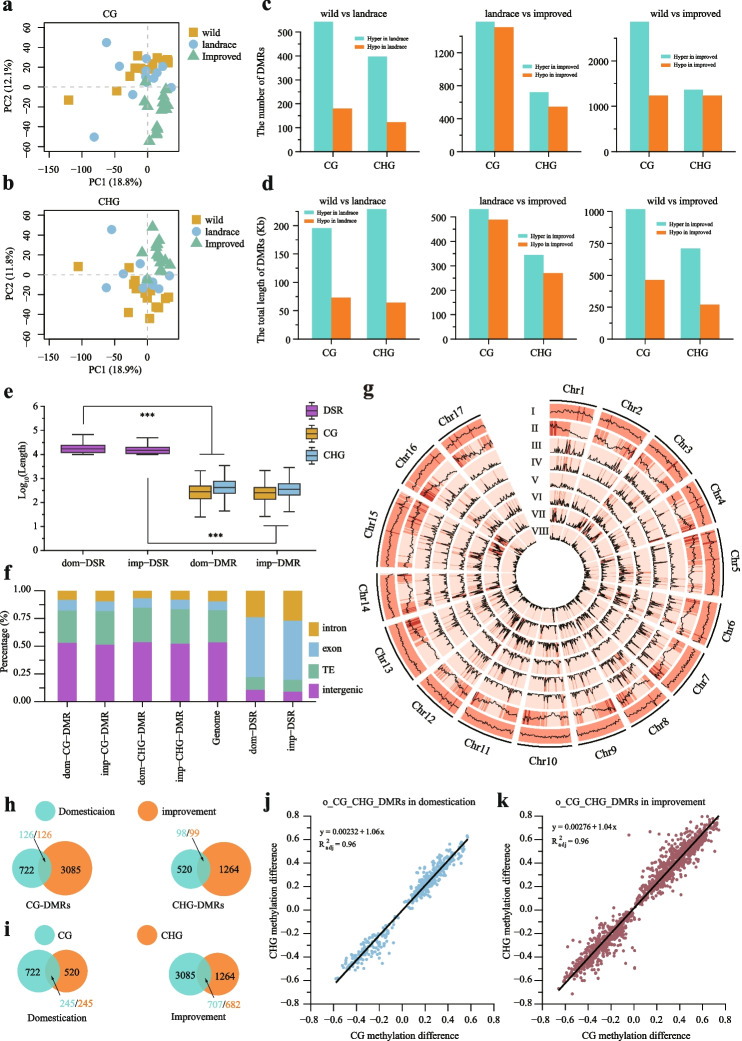
Comparisons of differentially methylated regions (DMRs) in the wild vs. landrace and landrace vs. improved populations. Principal component analysis (PCA) plots of DNA methylation levels in the CG (**a**) and CHG (**b**) contexts. The yellow squares represent the wild pear accessions, the blue points represent the landrace pear accessions, and the green triangles represent the improved pear accessions. **c** The number of hyper/hypo-DMRs in the wild vs. landrace, landrace vs. improved, and wild vs. improved comparisons. **d** The total lengths of hyper/hypo-DMRs in the wild vs. landrace, landrace vs. improved, and wild vs. improved comparisons. **e** Comparisons of the lengths of DNA sequence regions under selection (DSRs) and the CG and CHG context DMRs in the wild vs. landrace and landrace vs. improved comparisons (*P < 0.05; **P < 0.01; ***P < 0.001, two-tailed paired Student’s *t*-test). **f** Genomic compositions of the DSRs and DMRs, including TEs, introns, exons, and intergenic regions. **g** Distribution of the DMRs between the wild and landrace populations for the 17 pear chromosomes. Proceeding from the outer ring to the inner ring, the data represents TE density (I), gene density (II), dom-CG-DMR density (III), dom-CHG-DMR density (IV), imp-CG-DMR density (V), imp-CHG-DMR density (VI), dom-DSR (VII), and imp-DSR (VIII). **h** Overlap of DMRs in the wild vs. landrace and landrace vs. improved comparisons for the CG and CHG contexts. **i** Overlap of DMRs in the 2 methylation contexts in the wild vs. landrace (domestication process) and landrace vs. improved (improvement process) comparisons. Correlation analysis between methylation levels of the CG and CHG contexts in o_CG_CHG_DMRs during pear domestication (**j**) and improvement (**k**)

We performed principal component analysis (PCA) using the CG and CHG methylation levels, and the results showed that the wild and landrace pears were mixed, while the improved accessions were isolated (Fig. 2a, b), which agrees well with the classifications based on SNPs [21]. To examine the DNA methylation variation during pear domestication and improvement, we identified the differentially methylated regions (DMRs) using the “metilene” software [26]. For the domestication process, we identified 1242 DMRs, including 722 CG-DMRs and 520 CHG-DMRs. For the improvement process, we identified 4349 DMRs, including 3085 CG-DMRs and 1264 CHG-DMRs. Interestingly, we found more hypermethylated DMRs (hyper-DMRs) than hypomethylated DMRs (hypo-DMRs), and the total length of the hyper-DMRs was longer than that of the hypo-DMRs (Fig. 2c, d). This result provides further evidence to support the gradual increase in DNA methylation during pear domestication and improvement.

Compared to the DMRs, the DNA sequence regions under selection (DSRs) had longer lengths (Fig. 2e). We investigated the genomic composition of the DMRs and DSRs. Compared to the DSRs, the DMRs were preferentially located in intergenic regions. In contrast, DSRs were mainly found in the intron and exon regions, and the DMRs showed a more even genomic distribution than the DSRs (Fig. 2f, g). In addition, a total of 14–23% of DMRs were located in the upstream and downstream regions of genes (Additional file 2: Fig. S11).

Only a small fraction of the DMRs were shared between domestication and improvement processes (Fig. 2h). This result is similar to the selection sweep analysis in that only 3% of selection sweep regions (182 kb) are shared between domestication and improvement (Additional file 2: Fig. S12), which suggests that different selections have occurred at different stages. Interestingly, we found that approximately 50% of the CG-DMRs and CHG-DMRs overlapped in both the domestication and improvement processes (Fig. 2i). One possible reason is that the CG-DMRs and CHG-DMRs had undergone co-evolution during pear domestication and improvement. To examine this possibility, we divided the DMRs into three groups based on the different patterns of overlap: (1) o_CG_CHG_DMRs (CG-DMRs that overlap with CHG-DMRs, or CHG-DMRs that overlap with CG-DMRs), (2) u_CG_DMRs (unique CG-DMRs), and (3) u_CHG_DMRs (unique CHG-DMRs). We explored the CG and CHG methylation levels of the o_CG_CHG_DMRs and found that the CG and CHG methylation levels were highly positively correlated (Fig. 2j, k). In addition, the CG and CHG methylation levels were also highly positively correlated in the u_CG_DMRs and u_CHG_DMRs (Additional file 2: Fig. S13). These results indicate that the CG and CHG context methylation patterns might have co-evolved during pear domestication and improvement.

### The changes in genetic diversity are not correlated with DNA methylation changes during pear domestication and improvement

**Fig. 3:**
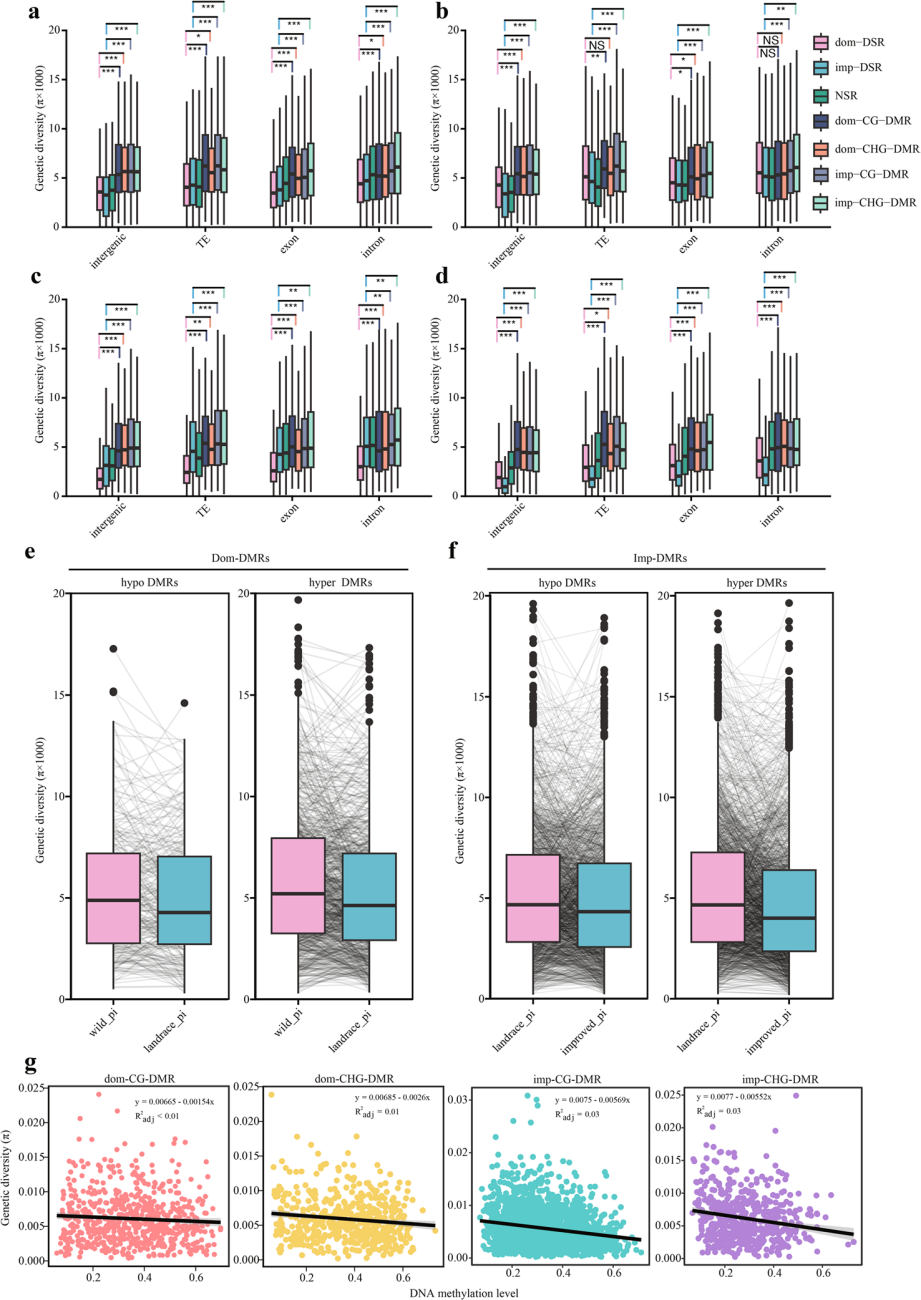
Genetic diversity changes in the DMRs. Comparison of genetic diversity between DMRs, DSRs, and NSRs in different genomic compositions for all pears (**a**) and the wild (**b**), landrace (**c**), and improved (**d**) populations (*P < 0.05; **P < 0.01; ***P < 0.001; NS, not significant; two-tailed paired Student’s *t*-test). The different genomic compositions include intergenic regions, TEs, exons, and introns. **e** Genetic diversity changes in the hyper DMRs and hypo DMRs during the domestication (Dom-DMRs) (**f**) and improvement processes (Imp-DMRs). Each black line represents one DMR. **g** The relationships between DNA methylation levels and genetic diversity in the dom-CG-DMRs, dom-CHG-DMRs, imp-CG-DMRs, and imp-CHG-DMRs

Genetic diversity is reduced during crop domestication because of genetic bottlenecks due to human-mediated selection. To explore the influence of DNA methylation on genetic diversity, we calculated and compared the genetic diversity of the DMRs, DSRs, and NSRs (outside of DMRs and DSRs) (Additional file 2: Fig. S14). In the pear genome, four genomic regions showed different diversity levels, and the introns had the highest genetic diversity (Additional file 2: Fig. S15). The genomic compositions of the DMRs and DSRs are significantly different (Fig. 2f). To eliminate the effects of different genomic compositions, we estimated the genetic diversity of the DMRs and DSRs in the different genomic regions. Compared to the DSRs, the DMRs had higher genetic diversity in all four genomic regions (Fig. 3a), and similar results were obtained for the three individual populations (Fig. 3b–d).

To further explore whether DNA methylation could lead to variation in genetic diversity, we calculated the diversity changes in both the hyper-DMRs and hypo-DMRs during pear domestication and improvement. If DNA methylation influences genetic diversity, we expect to observe a consistent pattern of change between DNA methylation and genetic diversity. However, we did not observe a consistent pattern of change between DNA methylation and genetic diversity in either the hyper-DMRs or the hypo-DMRs (Fig. 3e, f). We next calculated the correlations between DNA methylation levels and genetic diversity, but only weak positive correlations were detected (Fig. 3g). This result indicated that the higher genetic diversity in DMR is a natural characteristic and that DNA methylation variation does not directly influence genetic diversity.

### meQTL analysis of the genetic basis of the DMRs

**Fig. 4:**
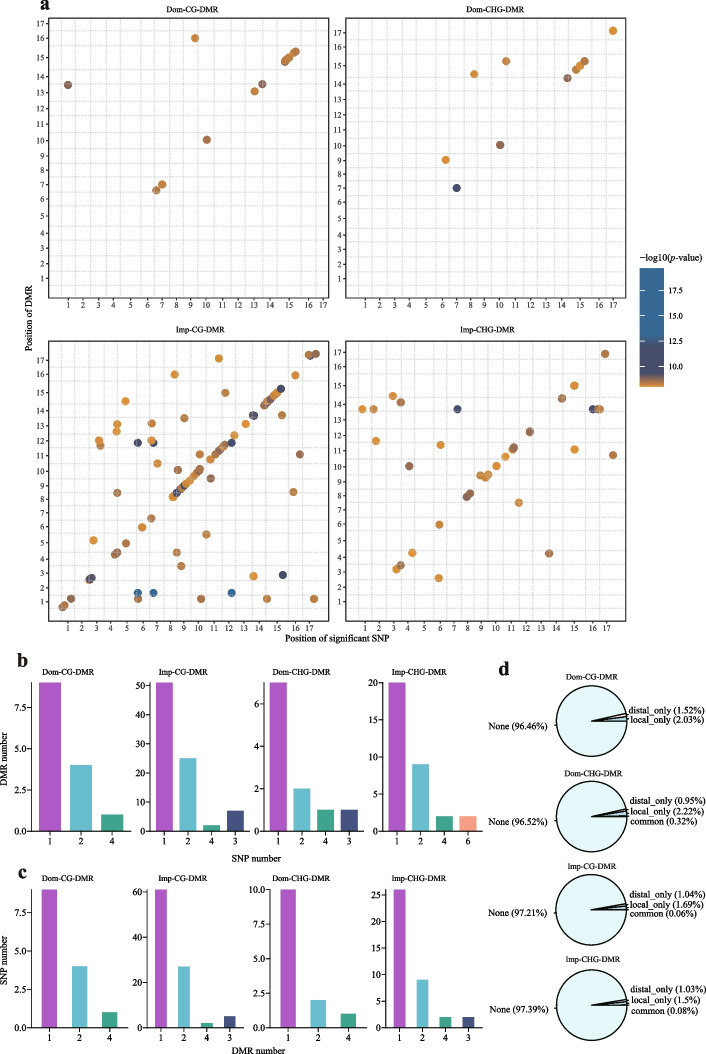
The genetic basis of the DMRs. **a** The chromosomal location distributions of the meQTLs identified for DMRs during pear domestication and improvement. The *x*-axis represents the genomic positions of significant SNPs, and the *y*-axis represents the genomic positions of the corresponding DMRs of the SNPs. The colors of the dots represent the *P*-value in the meQTL analysis. The meQTL significant threshold was set as 1.78 × 10^−9^ (0.01/*N*, *N*=5,618,948), and only significant meQTLs were plotted. Dom-CG-DMR represents the CG DMRs between wild and landrace accessions; Dom-CHG-DMR represents the CHG DMRs between the wild and landrace accessions; Imp-CG-DMR represents the CG DMRs between the landrace and improved pear accessions; Imp-CHG-DMR represents the CHG DMRs between the landrace and improved pear accessions. **b** Distribution of the number of significant SNPs per DMR. **c** Distribution of the number of significantly associated DMRs for each SNP. **d** Summary of the genetic basis for the CG and CHG DMRs during pear domestication and improvement

A previous study reported that genetic variation can affect DNA methylation levels [27]. To determine the genetic basis of DMRs during pear domestication and improvement, a methylation quantitative trait locus (meQTL) analysis was performed based on 5,618,948 SNPs as markers and using the DNA methylation level of each DMR as traits. We identified a total of 21, 18, 138, and 58 SNPs associated with the dom-CG-DMRs (CG DMRs between wild pears and landraces), dom-CHG-DMRs (CHG DMRs between wild pears and landraces), imp-CG-DMRs (CG DMRs between landraces and improved pears), and imp-CHG-DMRs (CHG DMRs between landraces and improved pears), respectively (Fig. 4a) (dom, domestication; imp, improvement). Using a previous method based on the distance between DMRs and associated SNPs, the meQTLs were divided into local and distal meQTLs (the “Methods” section). A total of 14 (67% of the total) and 13 (72% of the total) of the local meQTLs were identified as dom-CG-DMRs and dom-CHG-DMRs, respectively. Moreover, 80 (58% of the total) and 34 (59% of the total) of the local meQTLs were identified as imp-CG-DMRs and imp-CHG-DMRs, respectively. These results indicated that dom-DMRs and imp-DMRs tend to be controlled by local meQTLs. Most SNPs were only significantly associated with one DMR, and most DMRs were only tagged by one SNP (Fig. 4b, c).

Importantly, ~ 97% of DMRs were not associated with SNPs in each methylation context during pear domestication and improvement (Fig. 4d). This result indicated that these DMRs might represent unique information independent of genetic variation and, therefore, can be considered “pure DMRs.” Kyoto Encyclopedia of Genes and Genomes (KEGG) enrichment analysis was performed using genes near the “pure DMRs” in the CG and CHG contexts, respectively. “Pure-CG-DMRs” and “Pure-CHG-DMRs” were found to be associated with starch and sucrose metabolism. “Pure-CHG-DMRs” were associated with phenylpropanoid biosynthesis (Additional file 2: Fig. S16).

### Effects of DNA methylation on gene expression

**Fig. 5:**
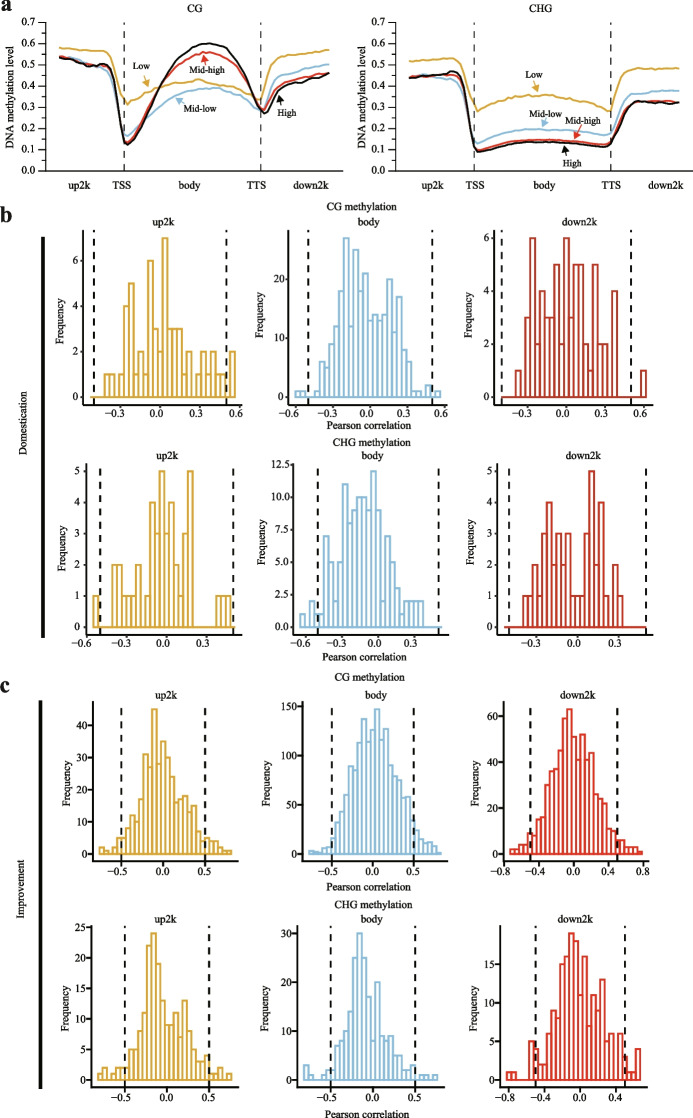
Correlation between DNA methylation and gene expression levels during pear domestication and improvement. **a** Relationships between CG and CHG methylation levels and expression levels for all genes in the 2-kb upstream, gene body, and 2-kb downstream regions. The genes were divided into four groups (low, mid-low, mid-high, and high) based on expression level. **b**, **c** Distribution of Pearson correlation coefficients between gene expression levels in the 2-kb upstream, gene body, and 2-kb downstream regions and methylation levels of the DMRs in the CG and CHG contexts during pear domestication (**b**) and improvement (**c**)

To test the effects of DNA methylation on gene expression, we divided all genes into four groups (low, mid-low, mid-high, and high) based on their expression levels. We then compared the DNA methylation levels of the four groups in the upstream 2-kb, gene body, and downstream 2-kb sequences of the genes. The results showed that the CG and CHG methylation levels in the upstream and downstream 2-kb regions (including the TSS and TTS) were negatively associated with gene expression levels. For the gene body regions, the CG and CHG methylation levels showed opposite correlations with gene expression levels: CG methylation was positively correlated with gene expression levels, while CHG methylation was negatively correlated with gene expression levels (Fig. 5a).

A total of 3159 genes identified during the pear domestication and improvement processes were found to be located near DMRs (Additional file 2: Fig. S17), in which genes might be regulated by DNA methylation. To determine the effect of DMRs on the relative expression of individual genes, we performed a Pearson correlation analysis between DMR methylation levels and gene expression levels in the 41 pear accessions. Because the genomic locations of the DMRs (2 kb upstream, 2 kb downstream, and gene body) usually exhibited differences in the relative direction of gene expression regulation, we performed Pearson correlation analysis for the three locations to distinguish their effects. As shown in Fig. 5b, c, we analyzed the distribution of the Pearson correlation coefficients between DMR methylation levels and gene expression levels. However, the results of Pearson correlation analysis in the upstream, downstream, and gene body regions were similar. Most DMR and gene pairs had low Pearson correlation coefficients (PCC < 0.5), while only a few genes had PCCs > 0.5 or < − 0.5 (Fig. 5b, c). The transcriptional profiling analysis showed no clear correlations between DNA methylation levels and gene expression levels of the genes near the DMRs.

### DNA hypermethylation is involved in the early ripening of fruits during pear domestication and improvement

**Fig. 6:**
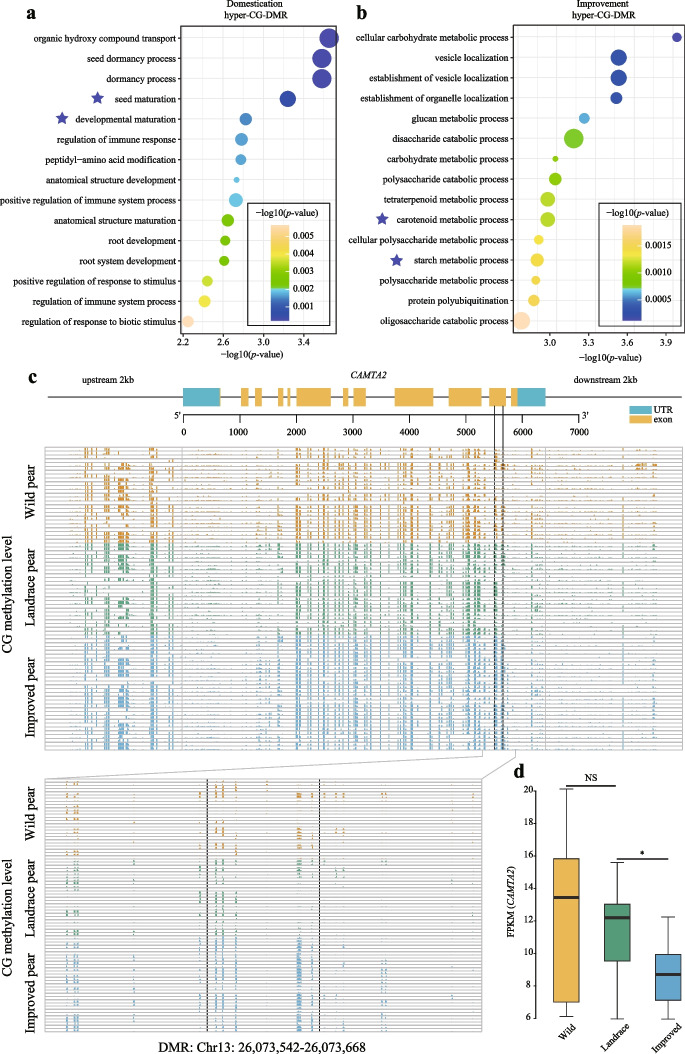
A hypermethylated DMR in the pear *CAMTA2* gene. **a** GO enrichment analysis (top 15 significant terms) of hyper-CG-DMR-associated genes during pear domestication. **b** GO enrichment analysis (top 15 significant terms) of hyper-CG-DMR-associated genes during pear improvement. The blue stars represent the GO terms associated with senescence. **c** The *CAMTA2* gene structure is shown at the top of the figure. Exons are represented by yellow-shaded boxes, introns are represented by black lines, and blue-shaded boxes represent the 5′ and 3′ UTRs. The bottom figure shows the CG methylation level of a DMR (Chr13:26,073,542–26,073,668) located in the *CAMTA2* gene in the wild, landrace, and improved pear populations. The entire gene (2-kb upstream, gene body, and 2-kb downstream regions) is shown in the upper panel, and the DMR in exon 11 is shown enlarged below. **d** Comparison of the expression levels (FPKM) of *CAMTA2* in the wild (yellow box), landrace (green box), and improved (blue box) pear populations (*P < 0.05; **P < 0.01; ***P < 0.001; NS, not significant; two-tailed paired Student’s *t*-test)

To explore the influence of DNA methylation on pear domestication, we performed Gene Ontology (GO) enrichment analysis of the genes located near the DMRs in both the domestication and improvement processes. Interestingly, we found that the hyper-CG-DMRs were enriched in terms associated with plant maturation and senescence, including seed maturation, development maturation, carotenoid metabolic process, and starch metabolic process (Fig. 6a, b). This result indicates that hypermethylation might associate with pear fruit ripening. For pear fruits, early ripening is a vital domestication and improvement trait because early ripening fruits usually have higher economic benefits. Therefore, one of the genes, *CAMTA2* (*EVM0036869.1*), which contains a hypermethylated DMR in the penultimate exon region (gene body), was selected for functional analysis because a previous study showed that *CAMTA* is associated with plant senescence [28] (Fig. 6c). Compared to wild pears, expression of the *CAMTA2* gene was continued downregulated in landrace and improved pear accessions (Fig. 6d). To further the relationship between DNA methylation and gene expression level of *CAMTA2*, we randomly selected 13 pear accessions to perform correlation analysis. The expression level of *CAMTA2* was measured by qRT-PCR. As a result, the DNA methylation level and expression level of *CAMTA2* exhibited a significant negative correlation (Additional file 2: Fig. S18).

**Fig. 7:**
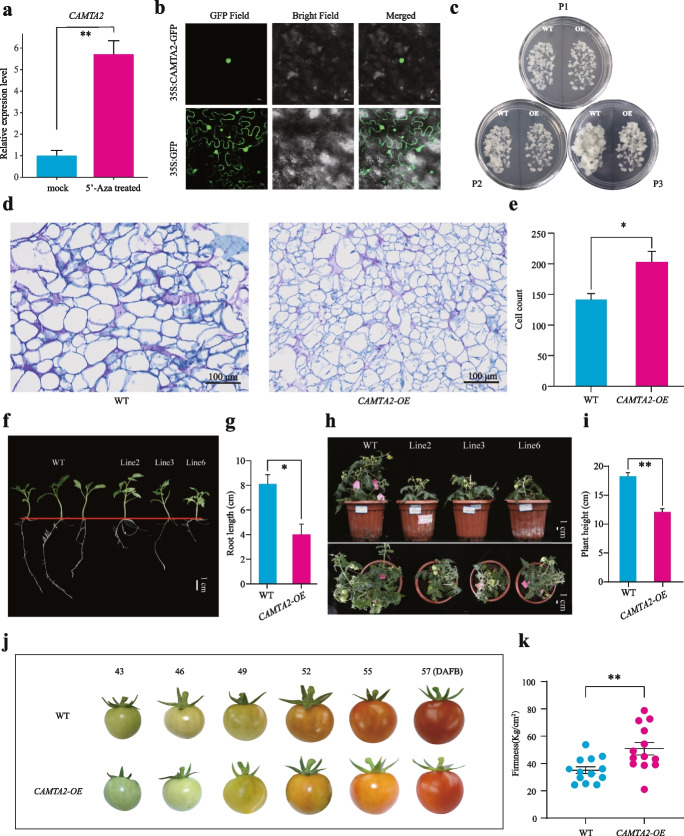
The function of *CAMTA2* in transgenic pear callus and transgenic tomato plants. **a** Relative expression of the *CAMTA2* gene in control and 5′-azacytidine (5′-Aza)-treated pear callus. **b** The CAMTA2-GFP fusion protein is localized to the nucleus of agroinfiltrated *Nicotiana benthamiana* leaf cells. **c** Growth of WT and *CAMTA2*-overexpressing (OE) pear callus. P1 = immediately after subculture, P2 = 14 days after subculture, and P3 = 24 days after subculture. **d**, **e** Cross-sections of pear callus stained with Toluidine Blue. The images show the number of cells in the same visual field of cross-sections of WT (**d**) and transgenic pear callus overexpressing *CAMTA2* (**e**). Scale bars = 100 μm. **f** Growth status of T_1_-generation transgenic seedlings before transplanting. Scale bar = 1 cm. **g** Statistical analysis of root lengths of WT and T_1_-generation *CAMTA2-OE* seedlings. **h** Phenotypes of WT and *CAMTA2-OE* transgenic tomato plants. Scale bars = 1 cm. **i** Statistical analysis of plant height in the WT and transgenic tomato plants. **j** Representative phenotypes of WT *CAMTA2-OE* transgenic tomato fruits at 43, 46, 49, 52, 55, and 57 days after full bloom (DAFB). **k** Statistical analysis of fruit firmness in the WT and *CAMTA2-OE* transgenic tomato fruits harvested at the red stage (*P < 0.05; **P < 0.01; ***P < 0.001, two-tailed paired Student’s *t*-test)

5-Azacytidine (5′-Aza) is an epigenetic drug that inhibits DNA methylation and has been widely used in many studies of DNA methylation in plants [29, 30]. To determine whether DNA methylation can affect the expression level of *CAMTA2*, we treated pear callus on an MS solid medium containing 5′-Aza to decrease the DNA methylation levels. The results showed that *CAMTA2* expression was significantly upregulated in the 5′-Aza-treated callus (Fig. 7a). We also treated pear fruit using 5′-Aza, and the results showed that fruit firmness after 5 days of 5′-Aza treatment was significantly higher than that of mock (Additional file 2: Fig. S19a), which indicated 5′-Aza treatment delayed fruit softening. Furthermore, bisulfite sequencing PCR indicated that the CG methylation level in candidate DMR (Chr13: 26,073,542–26,073,668) significantly decreased (Additional file 2: Fig. S19b). Meanwhile, qRT-PCR showed that *CAMTA2* was significantly upregulated in 5′-Aza treated samples than mock (Additional file 2: Fig. S19c). Therefore, the fruit and callus treatments showed similar results, showing that DNA methylation plays an essential role during the fruit ripening of pears by regulating the gene expression of *CAMTA2*.

To determine where the CAMTA protein is localized within the plant cell, *Agrobacterium tumefaciens* strains harboring the 35S:CAMTA::GFP fusion protein and control (35S::GFP only) constructs were infiltrated into leaves of young *Nicotiana benthamiana* plants and incubated for 3 days. Under a laser confocal microscope, we observed green fluorescence throughout the cell in the control group, while in the leaves infiltrated with the 35S:CAMTA::GFP construct, we observed green fluorescence only in the cell nuclei (Fig. 7b).

To further investigate the role of *CAMTA2* in pear fruit development, the empty vector and *CAMTA2* overexpression construct were stably transformed into pear callus, and qRT-PCR assays were used to identify the transgenic pear calli (Additional file 2: Fig. S20). Compared to the CK, the growth of the transgenic pear callus was repressed (Fig. 7c). Toluidine Blue staining showed that the number of cells in the transgenic pear callus was higher, and the cells were smaller compared to the CK in the same size visual field (Fig. 7d, e). qRT-PCR indicated that the expression of *ACO*, which encodes a key ethylene synthetase, was significantly decreased in transgenic callus compared to the WT control (Additional file 2: Fig. S21). This result indicated that overexpression of *CAMTA2* suppressed the growth and development of pear callus.

To confirm the role of *CAMTA2* in plant senescence and fruit ripening, *Agrobacterium tumefaciens* carrying the *CAMTA2* overexpression construct was used to transform tomato (T_0_ generation). We obtained T_1_-generation *CAMTA2*-positive transgenic tomato plants (Additional file 2: Fig. S22). Root lengths in the transgenic tomato seedlings were significantly shorter than in the WT control plants (Fig. 7f, g), and a dwarf phenotype also became apparent during transgenic tomato development (Fig. 7h, i). As shown in Fig. 7j, fruit development in the transgenic tomato lines lagged behind that of the control. WT tomato fruits ended the turning stage and entered the red stage at about 52 DAFB (days after full bloom), and ended the red stage at 56–57 DAFB, while the *CAMTA2*-OE transgenic tomato fruits only entered the turning stage around 52 DAFB and started the red stage at 56–57 DAFB. This result indicated that fruit ripening was delayed in the *CAMTA2* transgenic tomato plants. We next measured the postharvest firmness of fruits in the red stage, and the results showed that fruit firmness in the *CAMTA2* transgenic plants was significantly higher than in the WT (Fig. 7k). In addition, qRT-PCR assays indicated that transcription of the *ACO* gene was significantly decreased in *CAMTA2* transgenic tomato compared to the WT control (Additional file 2: Fig. S23). These results strongly suggest that overexpression of *CAMTA2* in tomatoes delayed fruit ripening by repressing ethylene synthetase.

## Discussion

### DNA methylation reprogramming during pear domestication and improvement

The reprogramming of DNA methylation levels is a common phenomenon in the plant kingdom and plays broad and critical roles during plant development and fruit ripening. The first study associated with the reprogramming of DNA methylation was reported during the tomato ripening process. *SIDML2*-mediated DNA demethylation is important for fruit ripening in tomatoes through its activation of the expression of ripening-related genes, including genes related to flavor synthesis, ethylene synthesis and signaling, and cell wall hydrolysis [31]. Interestingly, unlike tomatoes, a global increase in DNA methylation was reported during orange fruit ripening. Huang et al. reported that hypermethylation might be caused by a decreased expression of DNA demethylase genes and could further lead to a reduction in the expression of genes associated with photosynthesis and an increase in the expression of genes related to the abscisic acid response [7]. In *Arabidopsis*, Yuan et al. reported that DNA demethylation regulates the expression of senescence-associated genes (SAGs) during the leaf senescence process and affects the leaf senescence. Compared to wild-type (WT), SAGs were hypermethylated and exhibited remarkably downregulated expression in *demeter-like 3* (*dml3*) *Arabidopsis* plant, and leaf senescence was delayed [32]. The results of these three studies suggest that the remodeling of DNA methylation is vital for fruit ripening and leaf senescence and that demethylases are essential for remodeling DNA methylation. In our study, we found that an increase in DNA methylation occurred during pear domestication and improvement, which specific pattern of DNA methylation in pear was opposite to the significant decrease during rice domestication [33], and the remodeling of DNA methylation was associated with the downregulated expression of three *DML1* genes. However, there was no significant difference in the expression of these three genes in the leaf tissues between wild, landrace, and improved pear populations (Additional file 2: Fig. S24), indicating that *PpyDML1.1*, *PpyDML1.2*, and *PpyDML1.3* exhibited tissue specificity expression. The result is consistent with previous reports that DNA methylation exhibited tissue specificity [34–36]. Genetic variations in promoter might affect the gene expression [37]. To explore the potential reason for the downregulation of DML1, we performed haplotype analysis of the promoter sequence of *PpyDML1.1*, *PpyDML1.2*, and *PpyDML1.3*. As a result, we found the apparent divergence of genotype in the promoter sequence of *PpyDML1.1*, *PpyDML1.2*, and *PpyDML1.3* between wild, landrace, and improved pears (Additional file 2: Fig. S25–S27). These results indicated that the downregulation of *PpyDML1.1*, *PpyDML1.2*, and *PpyDML1.3* might be caused by the variation in promoter sequence.

In plants, three cytosine methylation contexts exist: CG, CHG, and CHH. CG methylation is maintained by METHYLTRANSFERASE1 (MET1), and CHG methylation is maintained by CMT3 and CMT2. Although the enzymes that maintain CG and CHG methylation differ, we found that the CG and CHG methylation levels exhibited significant correlations in the DMRs during pear domestication and improvement. In soybeans, ~ 66% and ~ 75% of the CHG DMRs also showed CG methylation difference during soybean domestication and improvement [38]. This result indicates that CG methylation and CHG methylation are usually coupled to regulate gene expression, and CG and CHG methylation may show co-evolution during crop domestication. For example, the promoter of *OsHXK1* is hypomethylated at CG and CHG sites, which leads to an increase in *OsHXK1* expression, which in turn leads to a decrease in the starch content in rice [39]. While DNA methyltransferases maintain cytosine methylation in specific contexts, four DNA demethylases in *A. thaliana* (ROS1, DME, DML2, and DML3) can erase the methylation status of cytosines in all sequence contexts. The increase in DNA methylation is consistent with the decreased expression of *DML1* during pear domestication and improvement. Therefore, it is possible that DNA demethylases played important roles in the variation in DNA methylation that occurred during pear domestication and improvement.

### The relationship between DNA methylation and gene expression

It is well-documented that DNA methylation can regulate gene expression, and the hypermethylation of promoters generally inhibits gene expression [3]. For example, methylation of the *ROS1* (reactive oxygen species) gene promoter negatively regulates the *ROS1* gene transcription [40]. During tomato ripening, the expression of hundreds of genes is upregulated due to the loss of DNA methylation in their promoter regions [31]. Promoter DNA methylation inactivates gene transcription by promoting the binding of transcription repressors or by inhibiting the binding of transcription activation factors. In addition, promoter DNA methylation also regulates gene expression by mediating histone modifications or histone acetylation [41, 42]. DNA methylation can also promote gene expression in some instances, but the regulatory mechanism is unclear [43]. These results indicate a complex relationship between DNA methylation and gene expression. In our study, we divided all genes in the pear genome into four groups based on their expression level and estimated the relationship between expression and methylation levels. In addition to the promoter (upstream 2 kb) regions, we found that DNA methylation levels, in both the CG and CHG contexts, were highly positively correlated with expression in the region 2 kb downstream of the TSS. Interestingly, we found that CG methylation was enriched into the gene body regions, especially in the high and mid-high expression groups, consistent with results reported in *Arabidopsis* [9].

### DNA hypermethylation contributed to early fruit ripening during pear domestication and improvement

DNA methylation levels are strictly regulated during plant growth and development, which reflects the important role of DNA methylation in plant physiology [3]. Many studies have reported dramatic changes in DNA methylation at single gene loci or the whole genome in response to abiotic stresses such as cold, heat, drought, high salinity, and phosphate starvation [44–48]. Low temperature can promote plant flowering by decreasing whole-genome methylation, and this was considered to be one of the most plausible explanations for how vernalization promotes flowering in plants. This was experimentally verified when it was shown that 5′-Aza treatment could promote early flowering [49, 50]. In addition, DNA methylation levels are also closely related to plant senescence and fruit maturation [7, 31, 51, 52]. For example, in tomatoes, many genes containing RIPENING-INHIBITOR (RIN) binding sites in the promoter regions are demethylated during tomato fruit development and ripening, and treatment with 5′-Aza was shown to promote early ripening in tomato [30]. The *CAMTA* genes, which encode a family of transcription factors, play important roles in abiotic and biotic stress responses, plant senescence, and fruit maturation [53–56]. *CAMTA* gene expression is associated with ethylene, and the expression of *AtCAMTA3* in tomatoes was significantly activated after exogenous treatment with ethylene [57]. Nei et al. (2012) obtained a gain-of-function mutation in *SIGNAL RESPONSIVE1* (*SR1*, a *CAMTA*-family gene) and found that *SR1* is involved in ethylene-induced plant senescence by directly regulating *ETHYLENE INSENSITIVE3* (*EIN3*). The expression of cell wall-related genes (*PG1*, *PE1*, and *CEL2*) was upregulated in *SlSR4* mutants. These results indicate that *CAMTA* genes involve plant senescence and fruit ripening. In our study, we found that domestication-induced DNA hypermethylation reduced the expression of *CAMTA2*, which contributed to the early ripening of pear fruits. This DMR with DNA hypermethylation is located on the penultimate exon region (gene body) of *CAMTA2*. A previous study reported that DNA methylation in the gene body might influence gene expression by promoting chromatin densification and interacting with functional elements in transcribed regions [22, 58–66]. Experimental verification analysis indicated that *CAMTA2* plays an essential role in pear callus development and tomato ripening. The demethylase expression of tomato is increased during fruit ripening [31], while the demethylase expression of pear is decreased during fruit ripening, indicating different methylation levels due to different species. In our study, the tomato was used to verify DNA methylation-related genes in pears; the reasons were as follows: Firstly, the tomato was often used as a model plant for fruit research [67]. The pear transformation system is especially difficult to accomplish, and it takes a long time to wait for fruit phenotyping after the transformation [68–70]. Secondly, in our experiment, the overexpression of the pear *CAMTA2* gene in transgenic tomato plants exhibited a 500-fold higher expression level than that of WT (Additional file 2: Fig. S22) and showed ripening delaying in tomato fruit. All these results supported the idea that the *CAMTA2* gene function in pears and tomatoes is similar. Although the DNA methylation pattern between tomato and pear during fruit ripening is different, the overexpression of *CAMTA2* gene function in tomato is obvious; it indicated that there is little effect on the function verification of the *CAMTA2* gene using tomato as transgenic material. In summary, this result demonstrates that DNA methylation played an essential role in the mature stage of development during the pear domestication process.

## Conclusions

In our study, we found a global increase in DNA methylation during the pear domestication and improvement process. This increase in DNA methylation significantly correlated with the downregulated expression of *Demeter-like 1* (*DML1*). A total of 1242 and 4349 DMRs were identified in the pear domestication and improvement process, respectively. The genes near hyper-DMRs were significantly associated with plant senescence and fruit ripening. We verified the function of a hyper-DMRs associated gene, *CAMTA2*, that overexpression of *CAMTA2* inhibited fruit ripening. In short, our study reported an increased pattern of DNA methylation during the domestication and improvement of pear and suggests that the increased DNA methylation plays a vital role in regulating the ripening period of pear fruit.

## Methods

### Plant materials

We collected 41 pear (*Pyrus pyrifolia*) fruit accessions at maturity from the Wuhan Sand Pear Germplasm collection (Hubei Academy of Agricultural Sciences, China). These included 14 wild (PYW1-PYW14), 13 landrace (PYW1-PYW12), and 15 improved (PYW1-PYW15) pear accessions. For each accession, 2 biological replicates were collected from 2 different trees for whole-genome bisulfite sequencing (WGBS). We separated the fruit flesh and the fruit skin, and the skin and flesh samples were flash-frozen in liquid nitrogen and stored at − 80 °C. The flesh samples were used for whole-genome bisulfite sequencing (WGBS) and strand-specific RNA sequencing (ssRNA-seq), and the skin samples were used for whole-genome sequencing (WGS).

### Bisulfite-seq, WGS, and ssRNA-seq library construction and sequencing

A cetyltrimethylammonium bromide (CTAB) method was used to extract DNA from the pear samples using a DNA isolation kit (Tiangen, Beijing, China), and the extracted DNA (OD_260/280_ = 1.8–2.0; total content > 6 μg) was used to construct libraries for whole-genome bisulfite sequencing (WGBS) and whole-genome sequencing (WGS). WGBS and WGS libraries were prepared according to the protocols described in previous reports. For each pear accession, WGBS was performed with two biological replicates. Lambda DNA was used to estimate the bisulfite conversion rate. The WGBS library preparations were sequenced on an Illumina HiSeq 2500 instrument (150-bp paired-end reads). The WGS library was sequenced using the Illumina HiSeq 4000 system (150-bp paired-end reads). Total RNA was extracted from the 41 pear accessions using an RNA isolation kit (Tiangen, Beijing, China) and was then used to construct the strand-specific RNA sequencing (ssRNA-seq) library. The ssRNA-seq library was sequenced on an Illumina HiSeq 4000 (150-bp paired-end reads). For all 41 accessions, 3 biological replicates were performed for the ssRNA-seq.

### Resequencing analysis

The FastQC (v0.11.9, http://www.bioinformatics.babraham.ac.uk/projects/fastqc) software package was used to check the quality of WGS data using default parameters [71]. Trimmomatic (v0.39) was used to trim the low-quality reads and adaptor sequences using the following parameters: “adapter.fa:3:30:10; SLIDINGWINDOW:4:20; MINLEN:50”, and clean data were mapped to the “Cuiguan” genome using the BWA software using the following parameters: “mem -t 6 -k 32 -M” [72, 73]. The “MarkDuplicates” function of the Picard software was used to filter reads that mapped to multiple positions (https://github.com/broadinstitute/picard). SNPs were called using the Genome Analysis Toolkit (GATK v4.1.4) HaplotypeCaller [74, 75], in which the following parameters were applied: “QD < 2.0, FS > 60.0, MQ < 40.0, MQRankSum < −12.5, and ReadPosRankSum < − 8.0.” A total of 5,618,948 SNPs were identified after filtering using VCFtools (0.1.16) with a missing rate of ≤ 70% and MAF ≥ 0.05. The “consensus” function of Bcftools was used to construct pseudo-reference genomes for each pear accession [76]. SNPs from individual accessions were used to replace the corresponding nucleotides in the reference genome to generate a pseudo-reference genome for each accession.

We performed genome scanning for selective signals using *π* ratios and *F*_*ST*_. First, we measured the nucleotide diversity (*π*) and *F*_*ST*_ values in 10 kb size windows with a 1 kb step size using VCFtools. The windows with the top 10% *π* ratios (*π*_wild_/*π*_landrace_; *π*_landrace_/*π*_improved_) and top 10% *F*_*ST*_ (wild vs. landrace; landrace vs. improved) were defined as selective sweep regions (domestication process: *π* ratio > 1.509 and *F*_*ST*_ > 0.075; improvement process: *π* ratio > 1.918 and *F*_*ST*_ > 0.157). The “merge” function in the Bedtools software package was used to combine the redundant selective sweep regions.

### RNA-seq analysis

For each sequence library, read quality was evaluated using the FastQC (v0.11.9, http://www.bioinformatics.babraham.ac.uk/projects/fastqc) software package with default parameters [71]. The Trimmomatic software package (v0.39) was used to trim the low-quality reads and adaptor sequences using the following parameters: “adapter.fa:2:40:15, LEADING:30, HEADCROP:10, TRAILING:30, SLIDINGWINDOW:4:15, AVGQUAL:30, and MINLEN:100”. Subsequently, the trimmed reads were mapped to the “Cuiguan” genome using the HISAT2 software (v 2.2.1) using default parameters. Samtools software packages (v1.3.1) were used to format conversion and sort of “bam” file [77]. The “MarkDuplicates” function of the Picard software was used to filter reads that mapped to multiple positions (https://github.com/broadinstitute/picard). The gene expression level of each transcript was counted and normalized into fragments per kilobase of transcript per million mapped reads (FPKM) using the Cufflinks software (v2.2.1, https://github.com/cole-trapnell-lab/cufflinks) with default parameters [78]. Cuffdiff function within Cufflinks was used to identify differentially expressed genes between wild, landrace, and improved pear following parameters: “|log2(FoldChange)| ≥ 0.58; [FDR] ≤ 0.05.”

### WGBS analysis

We checked each WGBS library using FastQC (v0.11.9) with default parameters. Trimmomatic (v0.39) was used to trim the low-quality reads and adaptors using the following parameters: “adapter.fa:2:40:15 LEADING:30 HEADCROP:6 TRAILING:30 SLIDINGWINDOW:4:15 AVGQUAL:30 MINLEN:100”, and the clean data was mapped to the pseudo-reference genome for each accession using Bismark (v2.2.5) [79]. After filtering out the duplicate reads using “deduplicate_bismark” in the bismark software package, the methylation information for each cytosine site was extracted using “bismark_methylation_extractor” in the bismark software package. We only retained sites covered by > 3 mapped reads to obtain reliable methylation sites. To investigate the chromosome distribution of DNA methylation, we divided the chromosome into windows with 500 kb length. Then, we calculated the methylation level of each window. To investigate the distribution of DNA methylation franking genes/TEs, we extracted the upstream 2 kb, downstream 2 kb, and body regions of genes/TEs, and we divided them evenly into 20, 20, and 40 windows, respectively. Then, we calculated the methylation level of each bin.

To investigate the reason for the increase of DNA methylation during the pear decomposition and improvement process, we identified the demethylase and methyltransferase genes in the pear genome. The pear protein database was constructed by the “makeblastdb” function in blast software packages using the following parameters: “-subtype prot -parse_seqids,” and then was scanned by BLASTP using all *Arabidopsis* demethylase and methyltransferase proteins as queries (*E*-value < 1e−10). The protein sequence alignments for the demethylase and methyltransferase proteins were performed using the MUSCLE software [80]. The phylogenetic trees for the demethylase and methyltransferase proteins were constructed using the tree software (bootstrap value = 1000) using the “JTT+F+R3” and “VT+F+R4” models, respectively [81].

### DMR identification

A program written in Perl, “metilene” (v0.2-8) [26], was used for differentially methylated region (DMR) identification between wild and landrace accessions, between landrace and improved accessions, and between wild and improved accessions. The “Metilene” software was widely used in DMR identification in many plant species, such as maize [24], soybean [38], and tomato [25] [82–84]. First, we used the “metilene_input.pl” script to construct the input file of “metilene” by combining DNA methylation of wild, landrace, and improved pear accessions. Then, we used the “metilene” software to identify DMRs and filtered the candidate DMRs using “metilene_output.pl” in “metilene” with the parameters as follows: (1) at least eight cytosine sites in a DMR, (2) the distance between any two adjacent cytosine sites was < 300 bp, and (3) the average methylation level between the two populations was > 25%. Finally, we used a corrected *P*-value < 0.01 as the cutoff.

The chromosome distribution of DMRs between wild and landrace accessions, between landrace and improved accessions, and between wild and improved accessions were displayed using the circos software [85]. The “intersect” function of the Bedtools software was used to identify common DMRs between different context DMRs. We divided the DMRs into three groups based on the different patterns of overlap: (1) o_CG_CHG_DMRs (CG-DMRs that overlap with CHG-DMRs, or CHG-DMRs that overlap with CG-DMRs), (2) u_CG_DMRs (unique CG-DMRs), and (3) u_CHG_DMRs (unique CHG-DMRs). Then, to explore the methylation level change of CG and CHG context methylation, we used the “stat_poly_eq” function of the “ggpmisc” package to annotate a plot with adjusted R2 or the fitted model equation and used the “geom_smooth” function of “ggplot2” package to add smoothed conditional means/regression line. The length of DNA sequence regions under selection (DSRs) and CG and CHG DMRs were measured, and the length of DSRs and DMRs were compared using the “geom_boxplot” of the “ggplot2” package. The “intersect” function in the Bedtools software was used to calculate the genomic compositions of the DSRs and DMRs, including exon, intron, TE, and intergenic (upstream 2 kb and downstream 2 kb). The nucleotide diversity (*π*) was calculated using SNPs calling from whole-genome resequencing (WGS) data. The VCFtools (0.1.16) software was used to calculate the nucleotide diversity (*π*) of each DMR, DSR, and NSR (outside of DMRs and DSRs) using the following parameters: “--site-pi.”

### meQTL analysis

For each DMR, the methylation level was normalized using rank-based inverse normal transformation using the “RNOmni” package. We used 5,618,948 SNPs as markers to perform the meQTL using “emmax-intel64” in EMMAX with a mixed linear model (MLM) [86]. Kinship was calculated using the “emmax-kin” function in EMMAX. The population structure was estimated using the admixture (v1.3.0) software with default parameters. The meQTL significance threshold was set as 1.78 × 10^−9^ (0.01/*N*, *N* = 5,618,948). To filter the false associations caused by SNPs in LD (linkage disequilibrium), we performed LD analysis using the plink software [87], and only independent SNPs (*r*^2^ < 0.25) were retained. Based on the distances between meQTLs and DMRs, the meQTLs were classified as either distal or local meQTLs. The local meQTLs represent meQTLs that are within 1 Mb of the DMRs. The distal meQTLs represent meQTLs that are > 1 Mb away from the DMRs or are located on different chromosomes.

### Whole-genome de novo transposable element annotation

The transposable elements (TEs) in the “Cuiguan” pear genome were predicted and annotated using the Extensive de-novo TE Annotator (EDTA) pipeline (v2.0.1) [88]. EDTA parameters were set to the following: “--species others; --sensitive 1; --anno 1; --evaluate 1; --threads 20.”

### KEGG and GO enrichment analysis

The KEGG and GO enrichment analyses of the DNA methylation-related genes were performed using the Tbtools software [89]. The GO ontology file, “go-basic.obo,” was downloaded from http://purl.obolibrary.org/obo/go/go-basic.obo. The KEGG backend file, “TBtoolsKEGGMap.DB,” was downloaded from https://tbtools.cowtransfer.com/s/566e88227a0045. We obtained GO and KEGG background files of the “Cuiguan” genome using EGGNOG-Mapper (http://eggnog-mapper.embl.de/). We visualized the results of GO and KEGG using the “ggplot2” packages in R.

### Gene cloning

The coding sequence (CDS) of *CAMTA2* was amplified from pear fruit using Q5 High-Fidelity 2× Master Mix (New England Biolabs, Ipswich, MA, USA). The names and nucleotide sequences of primers used to amplify *CAMTA2* are given in Additional file 1: Table S4. The *CAMTA2* PCR products were gel-purified and cloned into the binary vector pCAMBIA1302 using the ClonExpress Entry One-Step Cloning Kit (Vazyme, China). Plasmid DNA was isolated from at least ten *E. coli* colonies for DNA sequencing. The correct fusion constructs were then transformed into *Agrobacterium tumefaciens* strain GV3101 using the freeze-thaw method.

### 5′-Aza treatment

Pear calli induced from the cultivar “Clapp’s Favorite” were used for 5′-Aza treatment, and the pear calli were sub-cultured as previously described [90]. For the 5′-Aza treatment, a solid medium was made by adding 400 μM 5′-Aza to the basic MS medium containing 30 g/L sucrose, 0.5 mg/L 6-BA, and 1.0 mg/L 2, 4-D. The control (blank) consisted of pear calli cultured on the same medium without 5′-Aza. The pear calli treated with 5′-Aza were cultured in the dark at 25 °C for 7 days, after which we quantified the expression of *CAMTA2* using qRT-PCR.

We collected ripening fruit of *Pyrus pyrifolia* “Akizuki” from pear gardens (Xuzhou, China) for 5′-Aza treatment. Fifty mM of 5′-Aza (Sigma, St. Louis, MO, USA) (dissolved in sterilized ddH_2_O) was injected directly into pear fruits, and fruits injected with ddH_2_O were used as negative control (Mock). One fruit was injected into five holes. Each hole was injected with 1 mL of 5′-Aza/ddH_2_O. The injected fruit were placed in a room at 25 °C. After 5 days, we measured the fruit firmness and collected flesh samples for isolating genomic DNA and RNA. Fruit firmness was measured three times around each injection hole using a Brookfield CT3 Texture Analyzer (N/cm2) (AMETEK Brookfield, Middleboro, MA) with the following parameters: “Trigger force 0.5N, puncture distance 10 mm.”

### Genetic transformation of pear callus

We performed genetic transformation of pear callus using a previously reported method [90]. Pear callus was immersed for 15 min in MS liquid medium containing a suspension of GV3101 cells (OD_600_ = 0.6) carrying either the *CAMTA2* overexpression vector or the empty pCAMBIA vector as the control. The infected pear callus was co-cultured on solid MS medium for 48 h and then transferred to solid MS subculture medium containing 20 mg/L hygromycin, 0.5 mg/L 6-BA, and 1 mg/L 2,4-D and cultured for at least 1 month in the dark at 24 °C. Transgenic hygromycin-resistant calli were subcultured every 15 days. The subcultured transgenic pear calli were kept in the dark at room temperature for 30 days.

### Genetic transformation of tomato

Tomato (*Solanum lycopersicum* cv. “Micro-Tom”) was transformed using *A. tumefaciens* GV3101 carrying the *CAMTA2* overexpression DNA constructs. T_1_-generation transgenic plants were analyzed using qRT-PCR with RNA extracted from inflorescence stems to determine the relative expression of the pear *CAMTA2* gene.

### RNA isolation and qRT-PCR

Total RNA was extracted using the Plant Total RNA Isolation Plus kit (Foregene; http://www.foregene.com). The first-strand cDNA was synthesized using the RevertAid First Strand cDNA Synthesis Kit (TransGen Biotech, China). The primers used for qRT-PCR were designed using the NCBI online tool (https://www.ncbi.nlm.nih.gov/tools/primer-blast/). The qRT-PCR reactions were performed using the LightCycler 480 SYBR GREEN Master system (Roche, USA). Each 10 μL reaction contained 150 ng of template cDNA, 0.5 μM of each primer, and 5 μL of LightCycler 480 SYBR GREEN I Master. All reactions were performed in 96-well plates with three replicates for each cDNA sample. The qRT-PCR amplification conditions were as follows: 3 min at 95 °C followed by 45 cycles of 95 °C for 3 s, 60 °C for 10 s, and 72 °C for 30 s. Fluorescence signal data was collected at 60 °C. The *Pyrus* GAPDH gene was used as the internal control. Gene expression levels were calculated using the 2^−ΔΔCt^ method [91]. At least three biological replicates were performed for each experiment.

### Bisulfite sequencing PCR analysis

We performed bisulfite sequencing PCR (BSP) to measure the DNA methylation level. We extracted the genomic DNA from the fruit and injected it with ddH_2_O and 5′-Aza using FastPure Plant DNA Isolation Mini Kit (Vazyme, Nanjing, China). We used a DNA Methylation Kit (CW2140M, CoWin Biosciences) to convert bisulfite. BSP was performed using Phanta® Max Super-Fidelity DNA Polymerase (Vazyme, Nanjing, China). The BS primers are listed in Additional file 1: Table S4. All fragments generated by BSP were introduced into the pEASY-Blunt Zero vector (CB501-01, TransGen Biotech) using ClonExpress® II One Step Cloning Kit (Vazyme, Nanjing, China). For each sample, ten individual clones were sequenced. The *CyMATE* software (http://www.cymate.org/) was used to analyze the sequencing results and measure DNA methylation levels.

### Supplementary Information

**Additional file 1:** **Table S1.** The sample information of 41 pears used in our study. **Table S2.** The BS conversion rate of 82 Bisulfite sequencing (BS-Seq) libraries. **Table S3.** The number of three context DNA methylation in each BS-seq library. **Table S4.** The information of the primers used in this study.

**Additional file 2:** **Fig. S1.** The computational pipeline utilized to examine the WGBS (whole-genome bisulfite sequencing) data in our study. **Fig. S2.** Read mapping rates for the WGBS data across the 41 pear accessions using biological duplicates. The mapping rates (a) and the number of methylated cytosine (mC) sites (b) with and without using pseudo reference genomes across all 41 accessions. **Fig. S3.** The Pearson correlation coefficients between DNA methylation levels and gene/TE densities. DNA methylation levels were positively associated with TE density but negatively correlated with gene density. **Fig. S4.** Distribution of DNA methylation levels across the upstream 2 kb, gene body, and downstream 2 kb regions of different TEs. **Fig. S5.** DNA methylation changes during pear domestication and improvement process. (a) Kernel density distribution of DNA methylation changes between wild and landrace pear populations. (b) Kernel density distribution of DNA methylation changes across landrace and improved pear population. **Fig. S6.** Phylogenetic examination of DNA demethylases (a) and methyltransferases (b) from *Arabidopsis thaliana* and *Pyrus pyrifolia*. **Fig. S7.** The expression levels of 14 methylation genes in wild, landrace, and improved pear populations. **Fig. S8.** Expression levels of nine demethylation genes in wild, landrace, and improved pear populations. **Fig. S9.** Selective sweep examination for three *Demeter-like1* (*DML1*) genes from *P. pyrifolia* during pear domestication and improvement. Comparisons of π (nucleotide diversity) in the three pear populations on two chromosomes and one contig. The red line represents the wild population, the blue line represents the landrace population, and the orange line represents the improved population. The grey region depicts the genomic locations of the *DML* genes. **Fig. S10.** Methylated cytosine site frequency spectrum (mC reads/total reads) for CG, CHG, and CHH contexts in the wild, landrace, and improved pear populations. The x-axes are composed of 10 different degree windows (from 0-10% to 90%-100%) for DNA methylation levels. The y-axes depict frequency distribution. The CG and CHG sites are highly methylated. However, the distribution is highly skewed towards unmethylation for the CHH sites. **Fig. S11.** The distribution of DMRs in the upstream and downstream regions of various genes. **Fig. S12.** Selection sweep analysis throughout pear domestication and improvement processes. (a) The identification of selective sweeps during pear domestication. Each point represents a 10-kb sliding window. If one sliding window with π ratio > 1.509 and *F*_*ST*_ > 0.075 is defined as a selective sweep region during domestication. (b) The identification of selective sweeps during pear improvement. Each point represents a 10-kb sliding window. If one sliding window with π ratio > 1.918 and *F*_*ST*_ > 0.157 is defined as selective sweep regions during domestication. (c) Overlap of selective sweep region in the wild vs. landrace (domestication process) and landrace vs. improved (improvement process) comparisons. **Fig. S13.** Correlation analyses between methylation levels of the CG and CHG contexts in the u_CG_DMRs (a) and the u_CG_DMRs (b) during pear domestication. Correlation analysis between the methylation levels of the CG and CHG contexts in the u_CG_DMRs (c) and the u_CG_DMRs (d) throughout pear improvement. **Fig. S14.** Genetic diversity comparisons between DMRs, DSRs, and NSRs across all pears and the wild, landrace, and improved populations (*P < 0.05; **P < 0.01; ***P < 0.001, two-tailed paired Student’s *t*-test). **Fig. S15.** Genetic diversity differences in various genomic backgrounds for the wild, landrace, and improved populations across all pears. The four different genomic backgrounds exhibited varying degrees of diversity, and the introns had the highest levels of genetic diversity. **Fig. S16.** KEGG enrichment analysis of genes linked to“Pure-CG-DMRs” (a) and “Pure-CHG-DMRs” (b). **Fig. S17.** The number of genes identified near DMRs in wild vs. landrace, landrace vs. improved, and wild vs. improved comparisons. **Fig. S18.** Correlation analysis between DNA methylation level of DMRs (Chr13:26073542-26073668) and the expression level of *CAMTA2*. **Fig. S19.** 5’-Aza, and ddH_2_O (mock) were injected into pear fruits. (a) Comparison of fruit firmness between 5’-Aza treated samples and mock. (b) Comparison of CG methylation level (mCG) gene expression levels in candidate DMR (Chr13: 26,073,542-26,073,668) between 5’-Aza treated samples and mock. (c) Comparison of gene expression levels of *CAMTA2* between 5’-Aza treated samples and mock (*P < 0.05, two-tailed paired Student’s *t*-test).** Fig. S20.** Expression levels of *CAMTA2* in callus tissues of wild-type (WT) and three *CAMTA2*overexpression lines (*P < 0.05; **P < 0.01; ***P < 0.001; ****P < 0.0001; NS = not significant, two-tailed paired Student’s *t*-test).** Fig. S21.** Expression level of the *ACO* gene in wild-type (WT) and *CAMTA2*-overexpressing pear callus tissue (*P < 0.05; **P < 0.01; ***P < 0.001; ****P < 0.0001; NS = not significant, two-tailed paired Student’s*t*-test).** Fig. S22.** Identification of *CAMTA2* in transgenic T_1_-generation tomato plants. (a) Detection of transgenic plants carrying *CAMTA2* through PCR amplification. WT represents wild-type plants (the cultivar‘Micro-Tom’; the negative control), P denotes the plasmid used for transformation (the positive control), and Lines 1-6 are transgenic tomato plants. (b) Quantitative RT-PCR was employed to determine the relative expression of *CAMTA2* in plants of the three transgenic lines (*P < 0.05; **P < 0.01; ***P < 0.001; ****P < 0.0001; NS = not significant, two-tailed paired Student’s *t*-test).** Fig. S23.** Relative expression levels of the *SlACO* gene in wild-type (WT; the cultivar ‘Micro-Tom’) and transgenic *CAMTA2*-overexpressing (*CAMTA2-OE*) tomato plants *P< 0.05; **P < 0.01; ***P < 0.001; ****P < 0.0001; NS = not significant, two-tailed paired Student’s *t*-test).** Fig. S24.** Expression level of *PpyDML1.1, PpyDML1.2*, and* PpyDML1.3 *genes in the leaves of three wild, three landrace, and three improved pears.** Fig. S25.** The *cis*-acting element analysis and haplotype analysis of promoter sequences of *PpyDML1.1* genes.** Fig. S26.** The *cis*-acting elements analysis and haplotype analysis of promoter sequences of *PpyDML1.2* genes.** Fig. S27.** The *cis*-acting elements analysis and haplotype analysis of promoter sequences of *PpyDML1.3* genes.

**Additional file 3. **Review history. 

## Data Availability

The whole-genome bisulfite sequencing (WGBS) reads have been deposited into the NCBI sequence read archive (SRA) (https://www.ncbi.nlm.nih.gov/sra/) under BioProject accession number PRJNA1044459 [92]; the strand-specific RNA sequencing (ssRNA-seq) reads have been deposited into the NCBI sequence read archive (SRA) under BioProject accession number PRJNA1041946 [93]; raw genome re-sequencing reads have been deposited into the NCBI sequence read archive(SRA) under BioProject accession number of PRJNA1041944 [94]. No other scripts and software were used other than those mentioned in the “Methods” section.
