# Supplementary material for: Increased DNA methylation contributes to the early ripening of pear fruits during domestication and improvement
Source: Genome Biol. 2024 Apr 5;25:87. doi: 10.1186/s13059-024-03220-y (PMC10996114; doi:10.1186/s13059-024-03220-y)
Supplement: Supplementary file 2 — Additional file 2: Fig. S1. The computational pipeline utilized to examine the WGBS (whole-genome bisulfite sequencing) data in our study. Fig. S2. Read mapping rates for the WGBS data across the 41 pear accessions using biological duplicates. The mapping rates (a) and the number of methylated cytosine (mC) sites (b) with and without using pseudo reference genomes across all 41 accessions. Fig. S3. The Pearson correlation coefficients between DNA methylation levels and gene/TE densities. DNA methylation levels were positively associated with TE density but negatively correlated with gene density. Fig. S4. Distribution of DNA methylation levels across the upstream 2 kb, gene body, and downstream 2 kb regions of different TEs. Fig. S5. DNA methylation changes during pear domestication and improvement process. (a) Kernel density distribution of DNA methylation changes between wild and landrace pear populations. (b) Kernel density distribution of DNA methylation changes across landrace and improved pear population. Fig. S6. Phylogenetic examination of DNA demethylases (a) and methyltransferases (b) from Arabidopsis thaliana and Pyrus pyrifolia. Fig. S7. The expression levels of 14 methylation genes in wild, landrace, and improved pear populations. Fig. S8. Expression levels of nine demethylation genes in wild, landrace, and improved pear populations. Fig. S9. Selective sweep examination for three Demeter-like1 (DML1) genes from P. pyrifolia during pear domestication and improvement. Comparisons of π (nucleotide diversity) in the three pear populations on two chromosomes and one contig. The red line represents the wild population, the blue line represents the landrace population, and the orange line represents the improved population. The grey region depicts the genomic locations of the DML genes. Fig. S10. Methylated cytosine site frequency spectrum (mC reads/total reads) for CG, CHG, and CHH contexts in the wild, landrace, and improved pear populations. The x-axes are [file 13059_2024_3220_MOESM2_ESM.docx]

**Additional file 2**


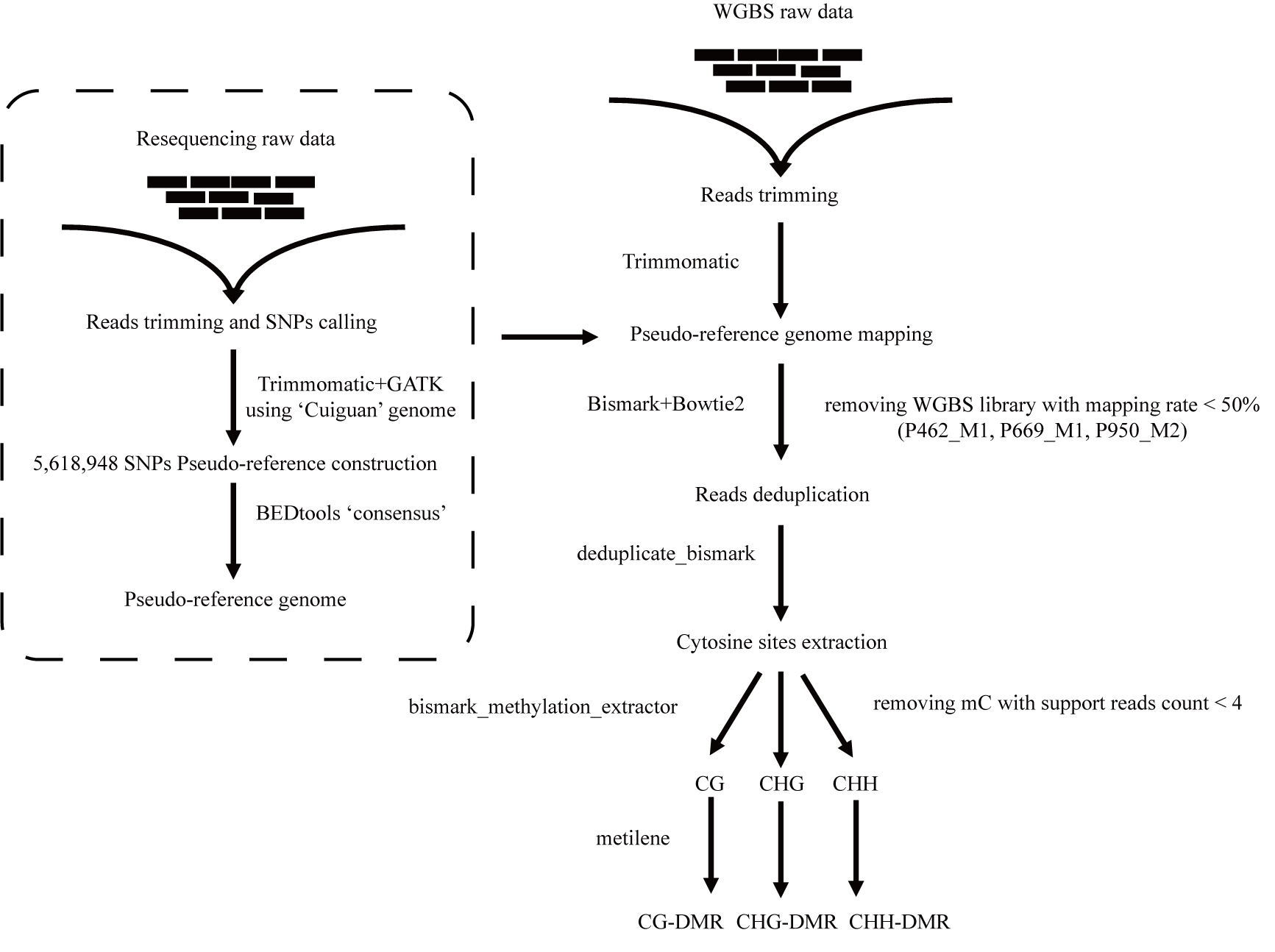


**Fig. S1**. The computational pipeline used to analyze the WGBS (whole-genome bisulfite sequencing) data in our study.


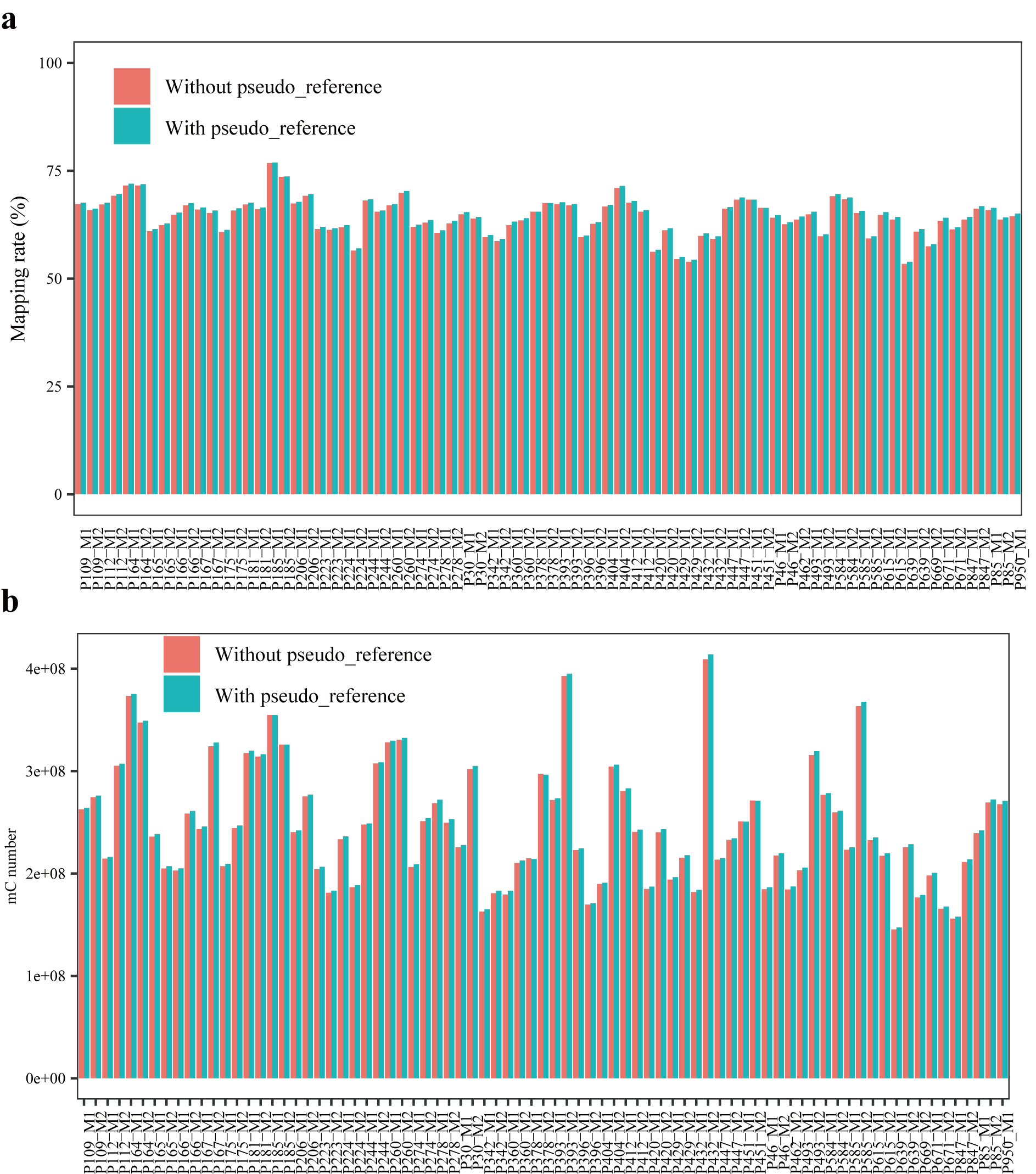


**Fig. S2**: Read mapping rates for the WGBS data in the 41 pear accessions with two biological replicates. The mapping rates (a) and the number of methylated cytosine (mC) sites (b) with and without using pseudo reference genomes in all 41 accessions.


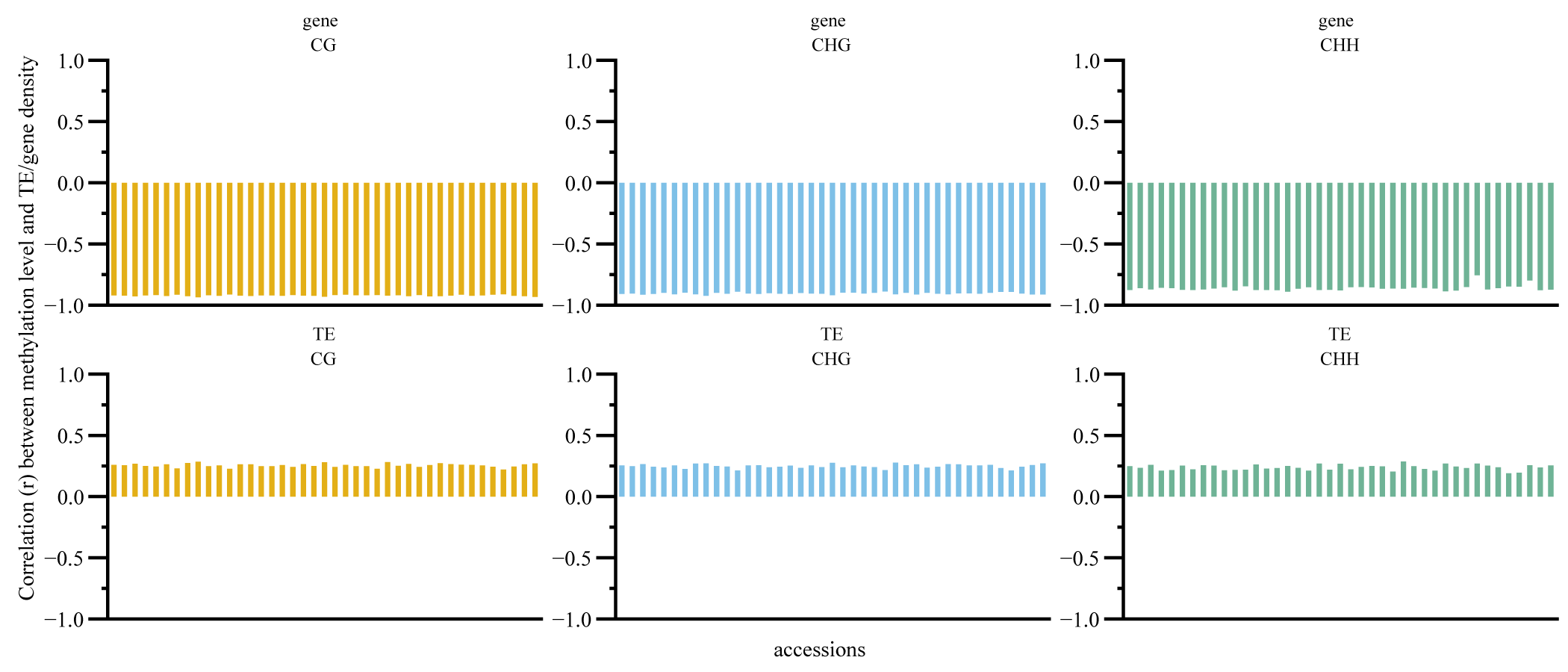


**Fig. S3**: The Pearson correlation coefficients between DNA methylation levels and gene/TE densities. DNA methylation levels were positively correlated with TE density but were negatively correlated with gene density.


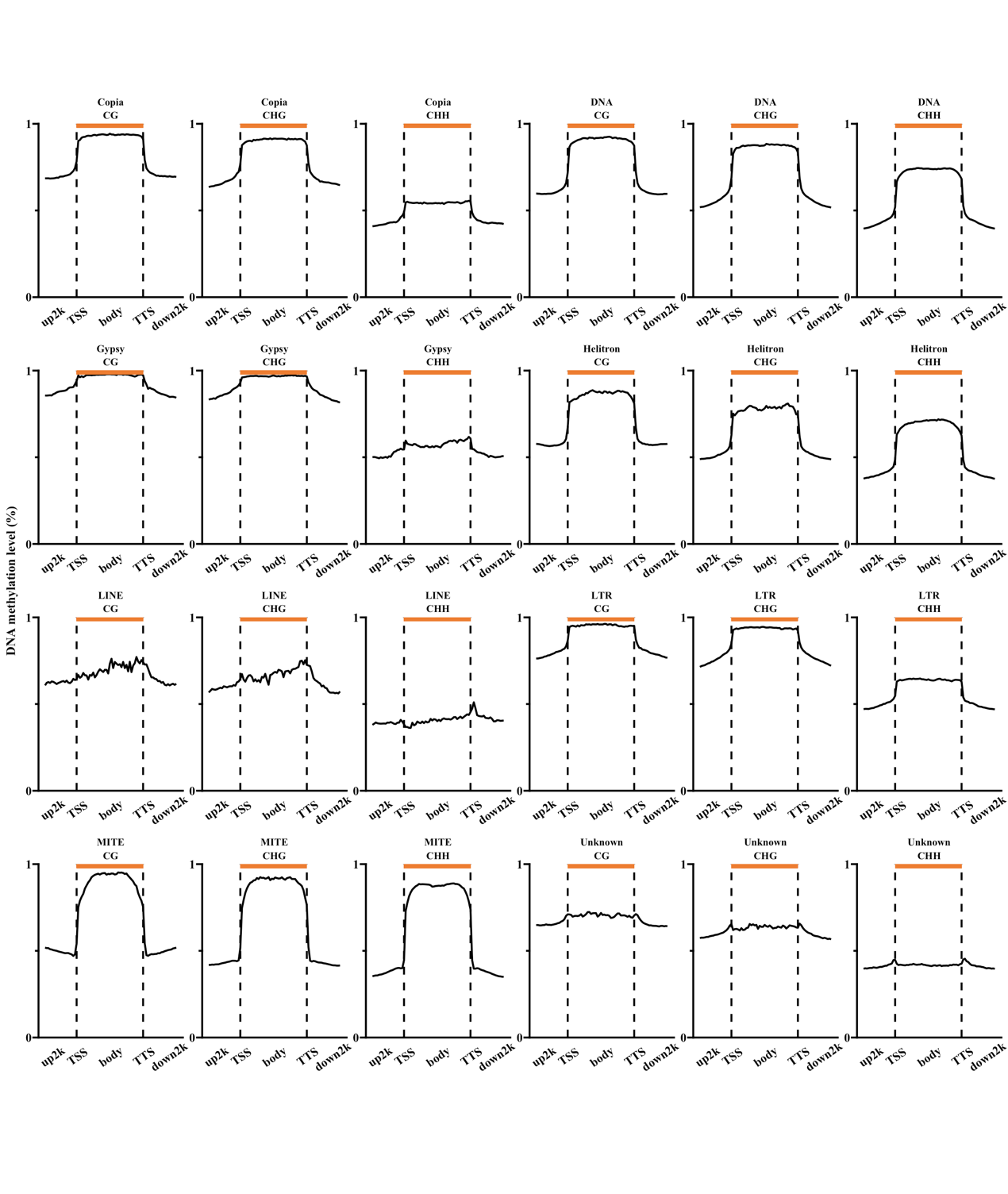


**Fig. S4**: Distribution of DNA methylation levels across the upstream 2k, gene body, and downstream 2k regions of different type TEs.


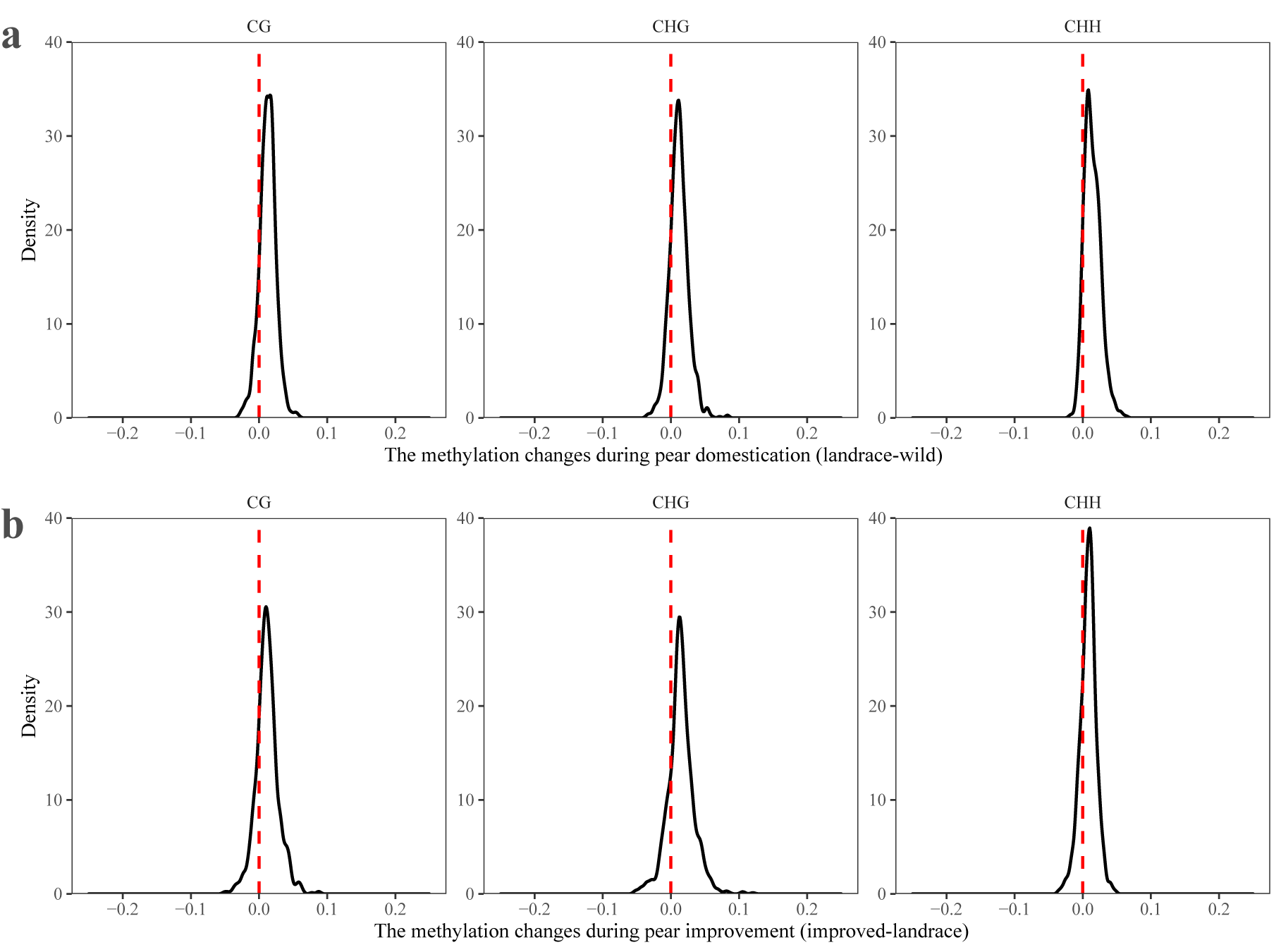


**Fig. S5**: The DNA methylation changes during pear domestication and improvement process. (a) Kernel density distribution of DNA methylation changes between wild and landrace pear population. (b) Kernel density distribution of DNA methylation changes between landrace and improved pear population.


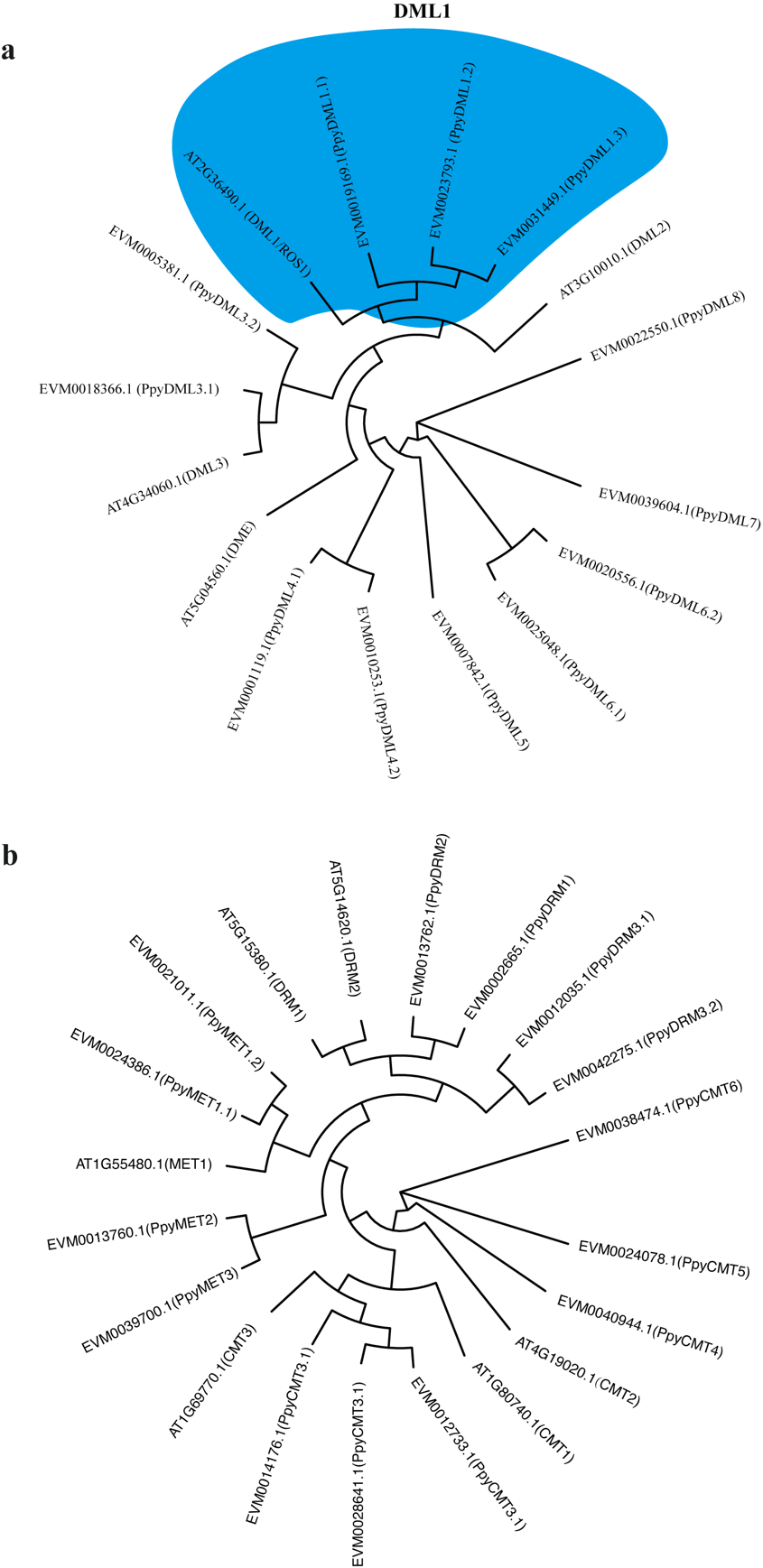


**Fig. S6**: Phylogenetic analysis of DNA demethylases (a) and methyltransferases (b) from *Arabidopsis thaliana* and *Pyrus pyrifolia*.


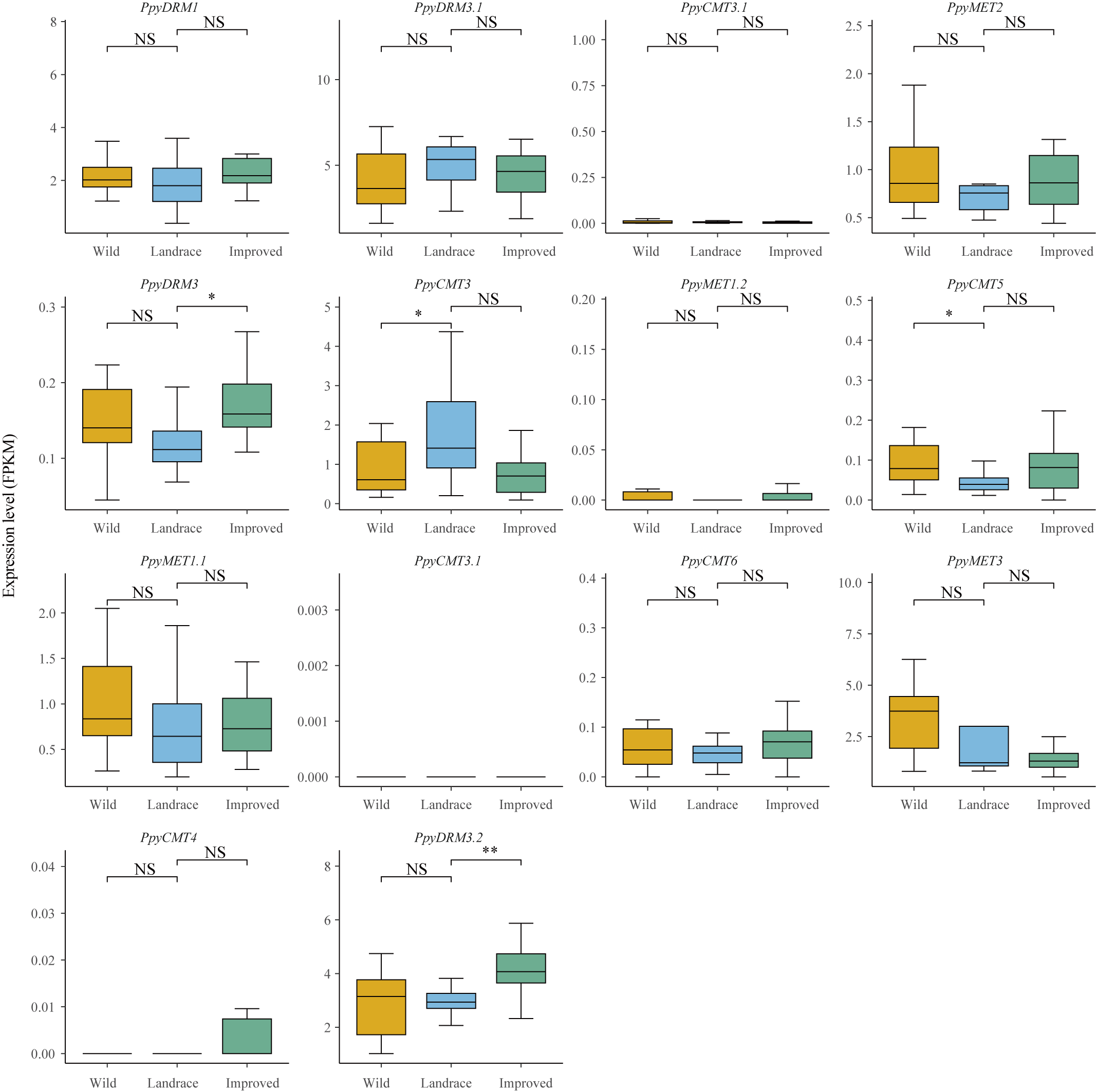


**Fig. S7:** The expression level of 14 methylation genes in wild, landrace and improved pear populations.


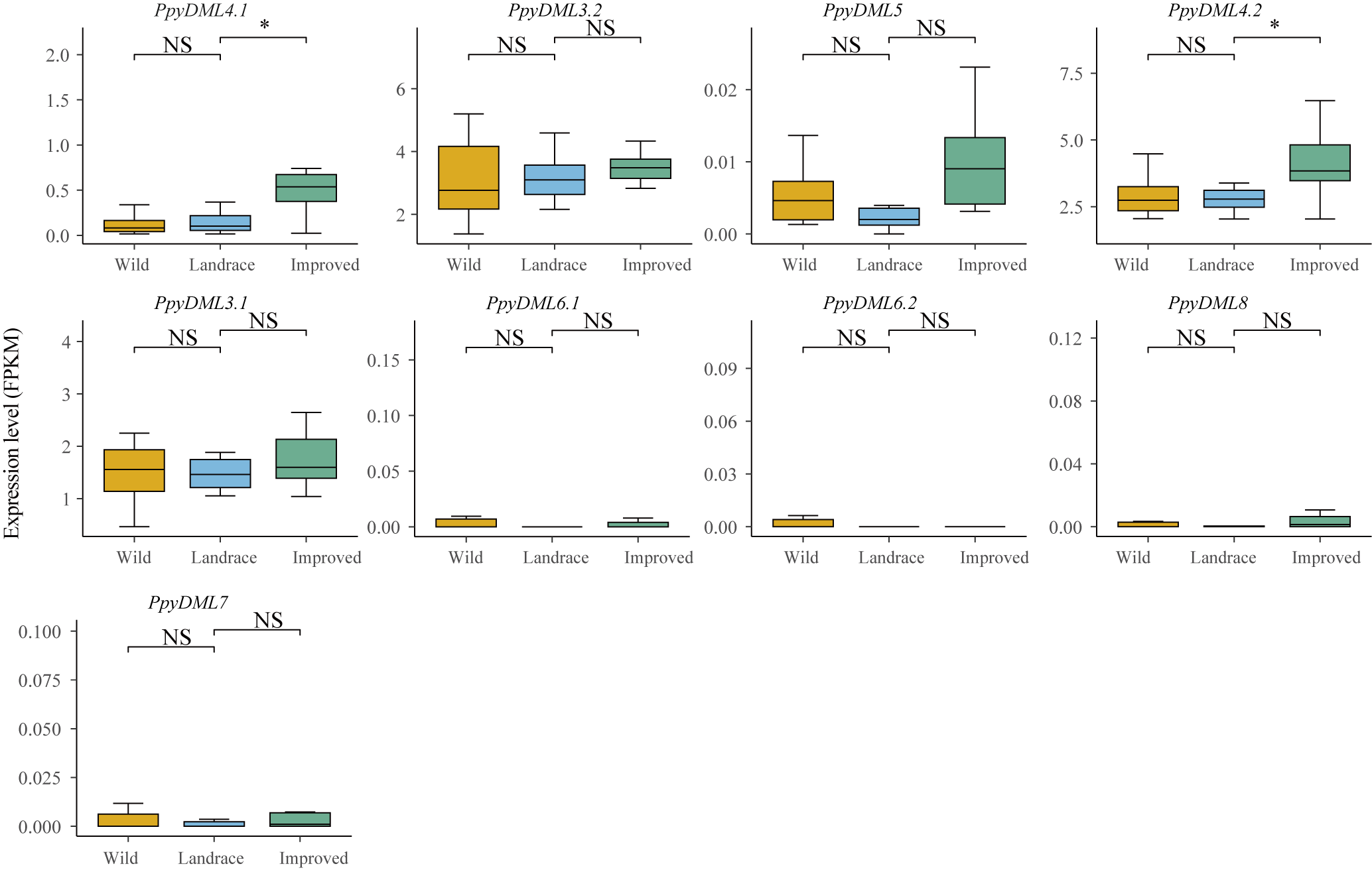


**Fig. S8:** The expression level of nine demethylation genes in wild, landrace and improved pear populations.


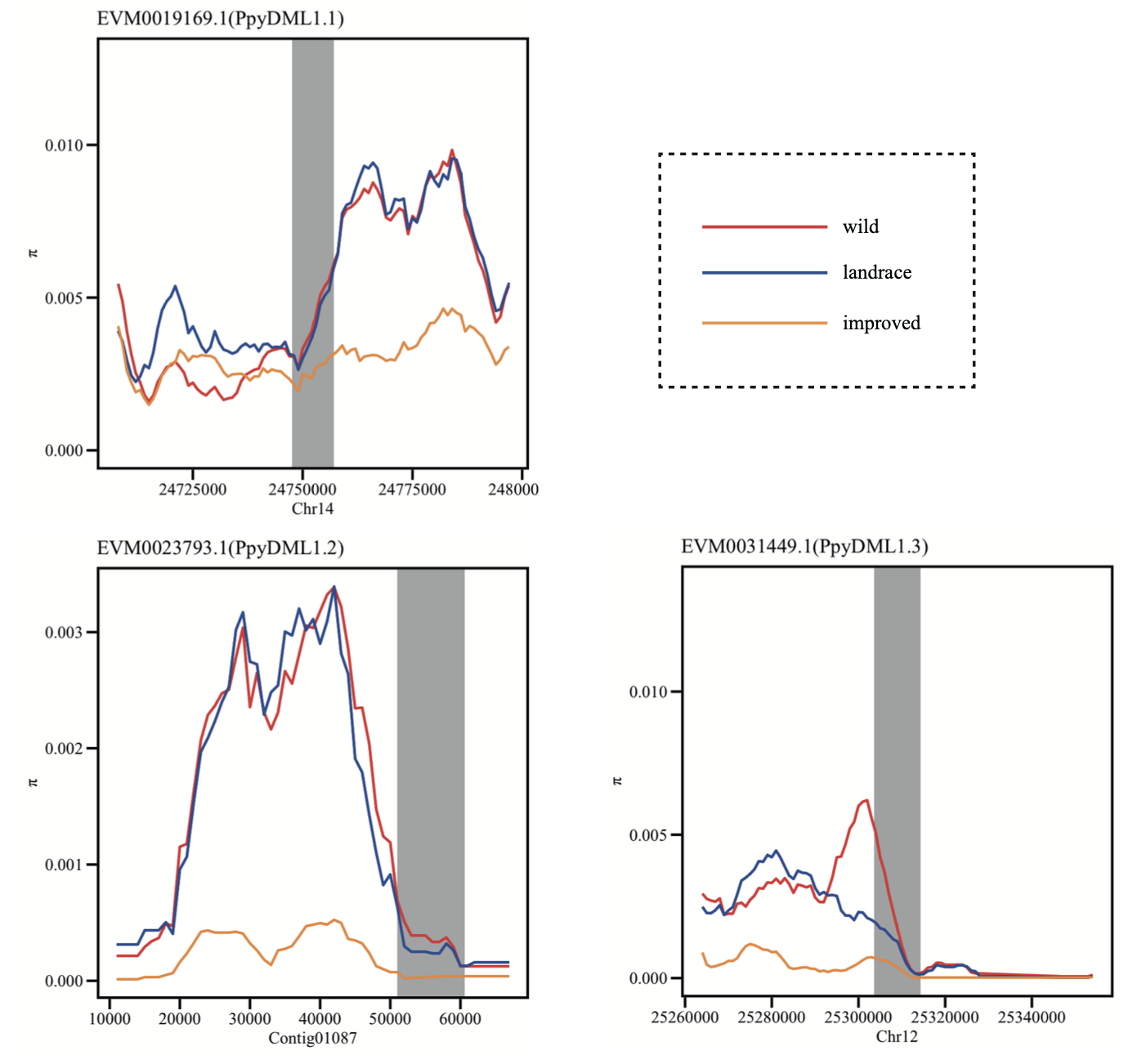


**Fig. S9**: Selective sweep analysis for three *Demeter-like1* (*DML1*) genes from *P. pyrifolia* during pear domestication and improvement. Comparisons of π (nucleotide diversity) in the three different pear populations on two chromosomes and one contig. The red line represents the wild population, the blue line represents the landrace population, and the orange line represents the improved population. The grey region shows the genomic locations of the *DML* genes.


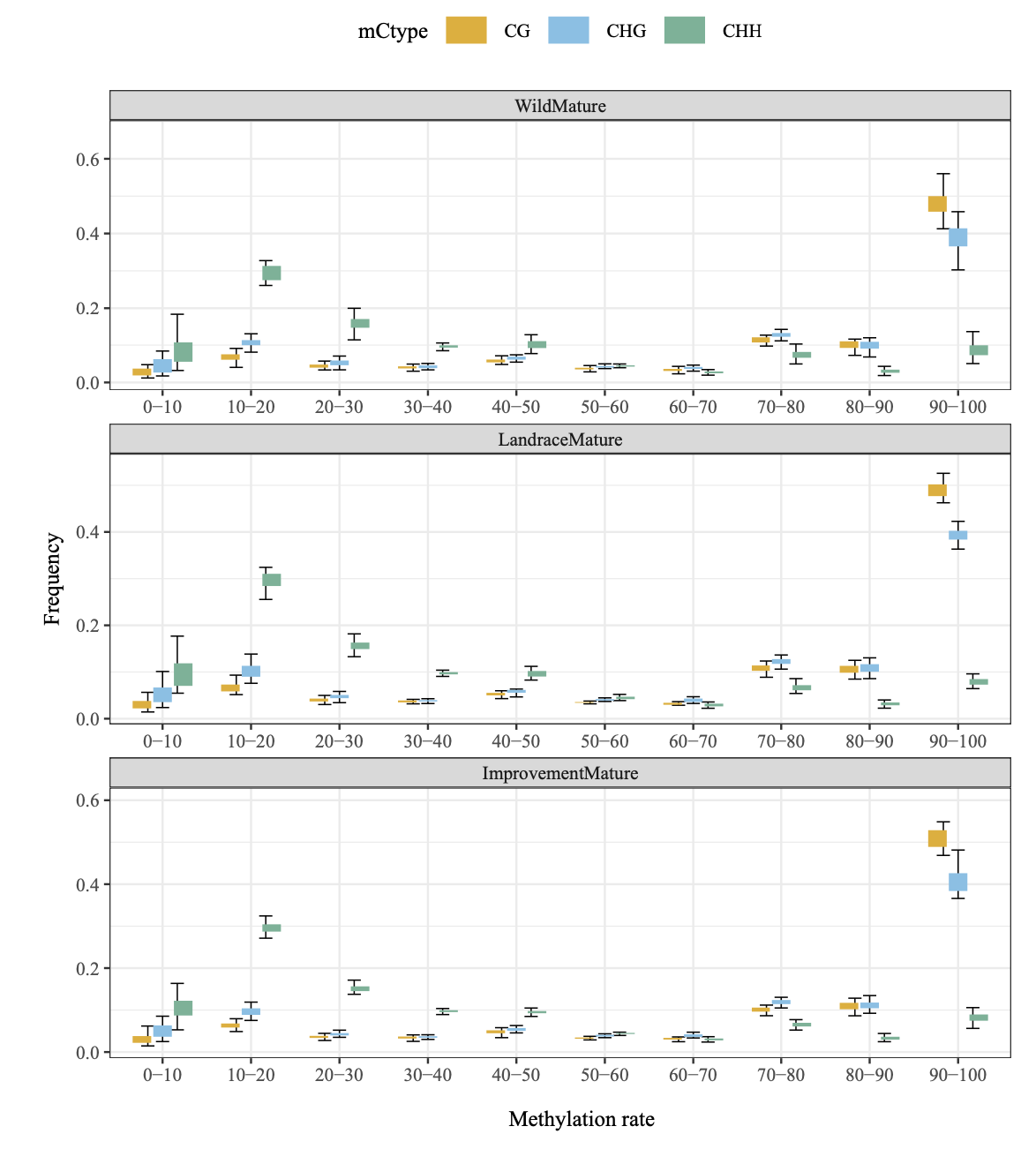


**Fig. S10**: Methylated cytosine site frequency spectrum (mC reads/total reads) for the CG, CHG, and CHH contexts in the wild, landrace, and improved pear populations. The x-axes consist of 10 different degree windows (from 0-10% to 90%-100%) for DNA methylation levels. The y-axes indicate frequency distribution. The CG and CHG sites are highly methylated. However, the distribution is highly skewed towards the unmethylated status for the CHH sites.


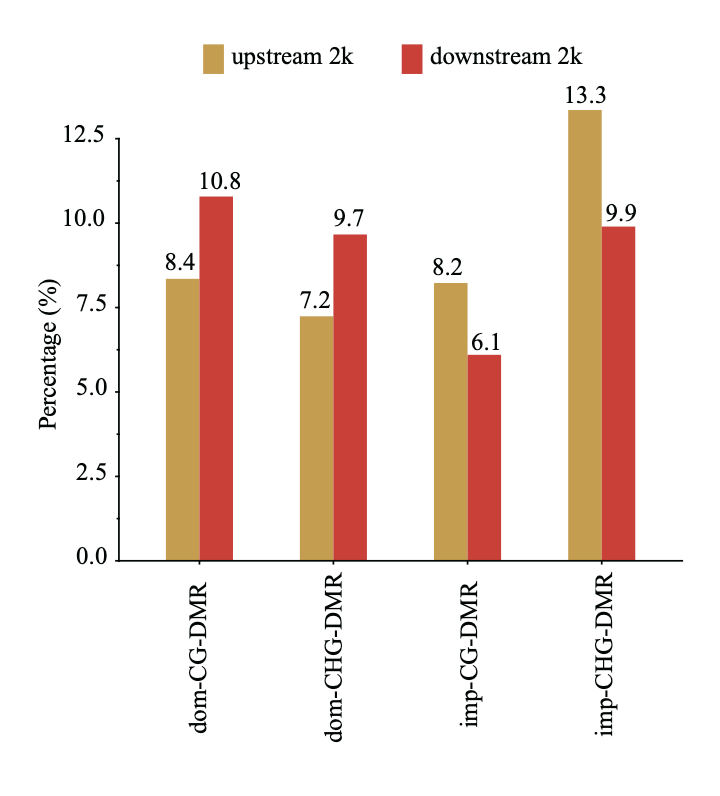


**Fig. S11**: The distribution of DMRs in the upstream and downstream regions of genes.


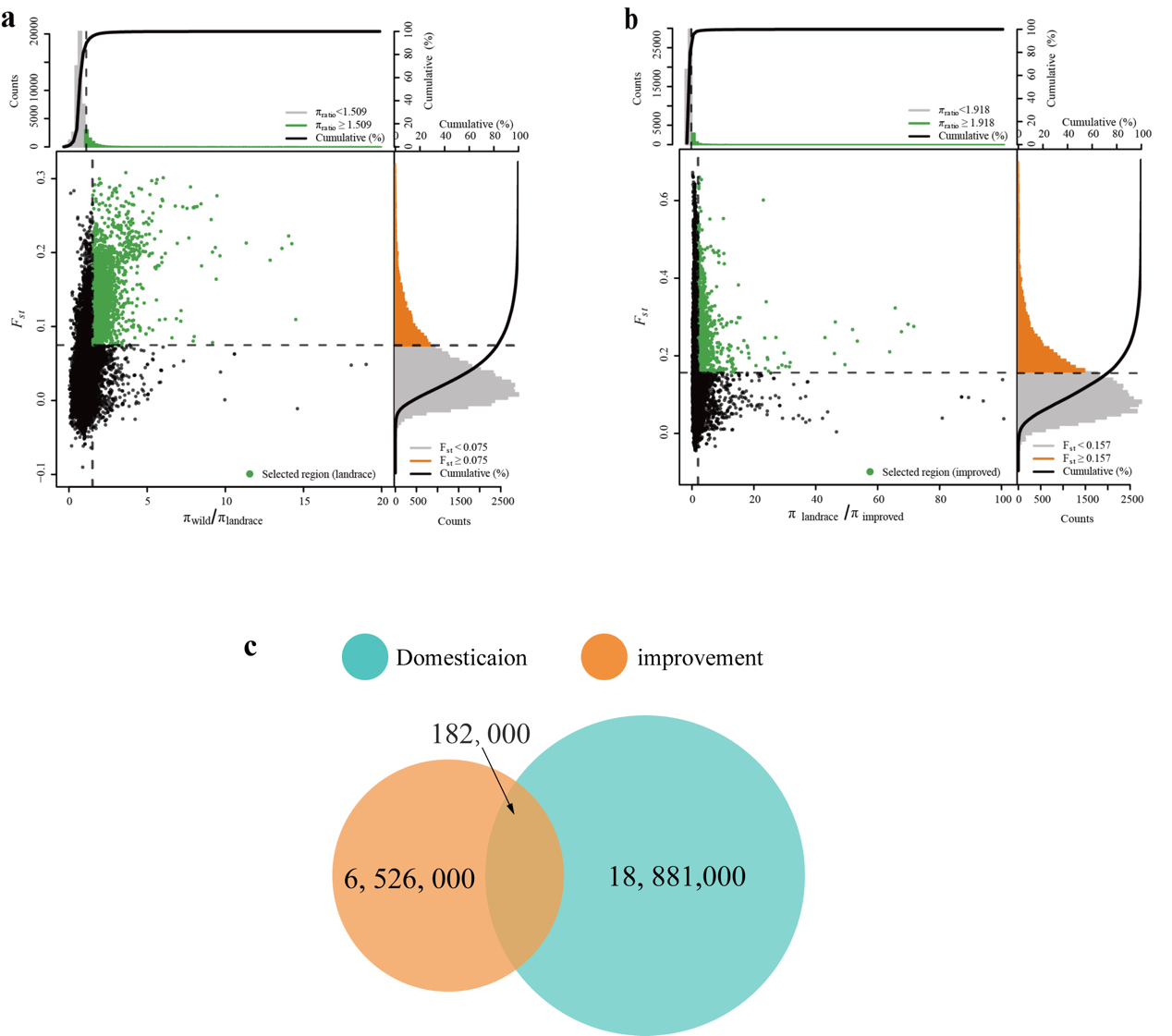


**Fig. S12**: Selection sweep analysis during pear domestication and improvement process. (a) The identification of selective sweeps during pear domestication. Each point represents a 10-kb slide window. If one slide window with π ratio > 1.509 and *F_ST_* > 0.075 is defined as selective sweep regions during domestication. (b) The identification of selective sweeps during pear improvement. Each point represents a 10-kb slide window. If one slide window with π ratio > 1.918 and *F_ST_* > 0.157 is defined as selective sweep regions during domestication. (c) Overlap of selective sweep region in the wild vs. landrace (domestication process) and landrace vs. improved (improvement process) comparisons.


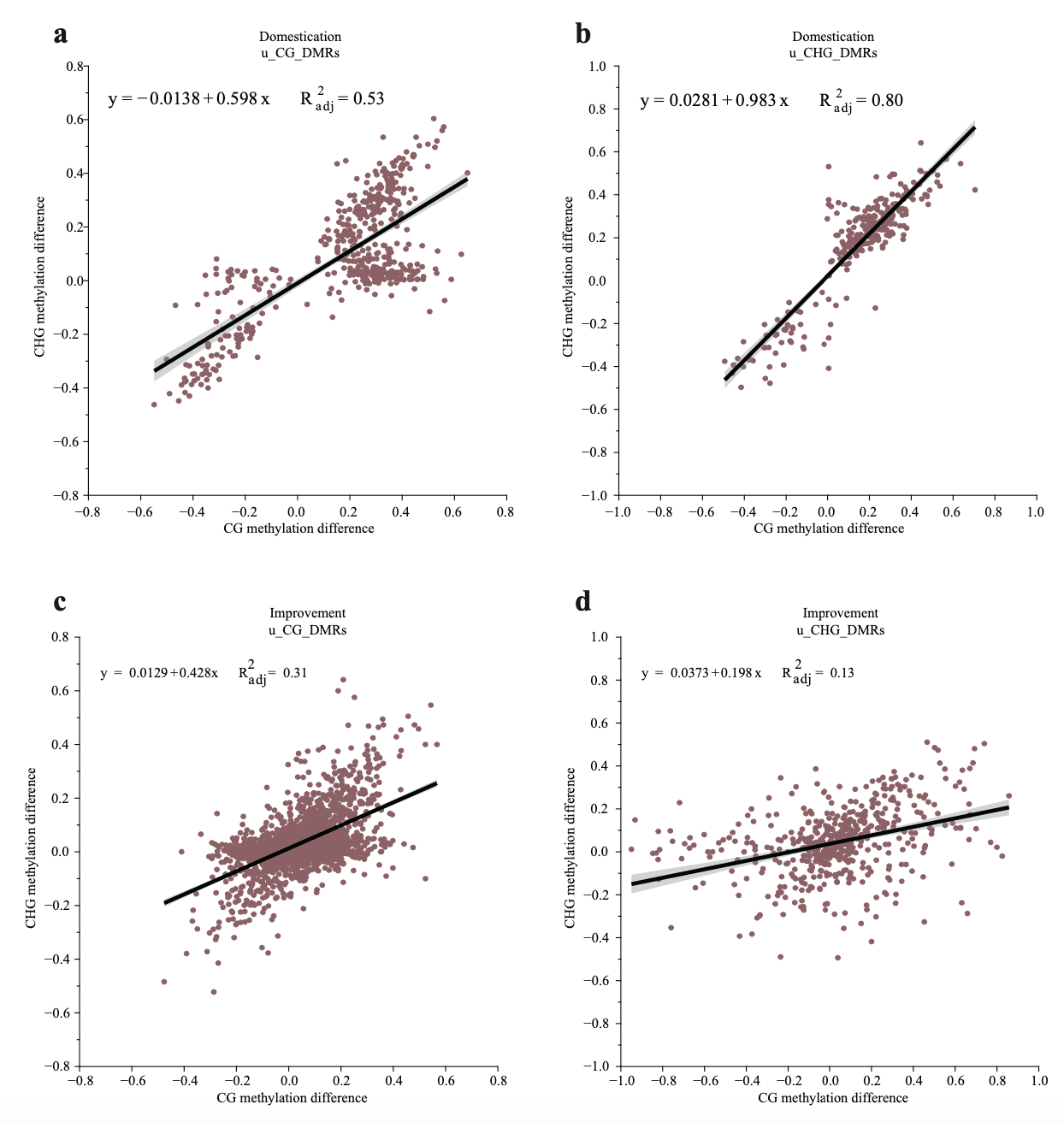


**Fig. S13**: Correlation analyses between the methylation levels of the CG and CHG contexts in the u_CG_DMRs (a) and the u_CG_DMRs (b) during pear domestication. Correlation analysis between the methylation levels of the CG and CHG contexts in the u_CG_DMRs (c) and the u_CG_DMRs (d) during pear improvement.


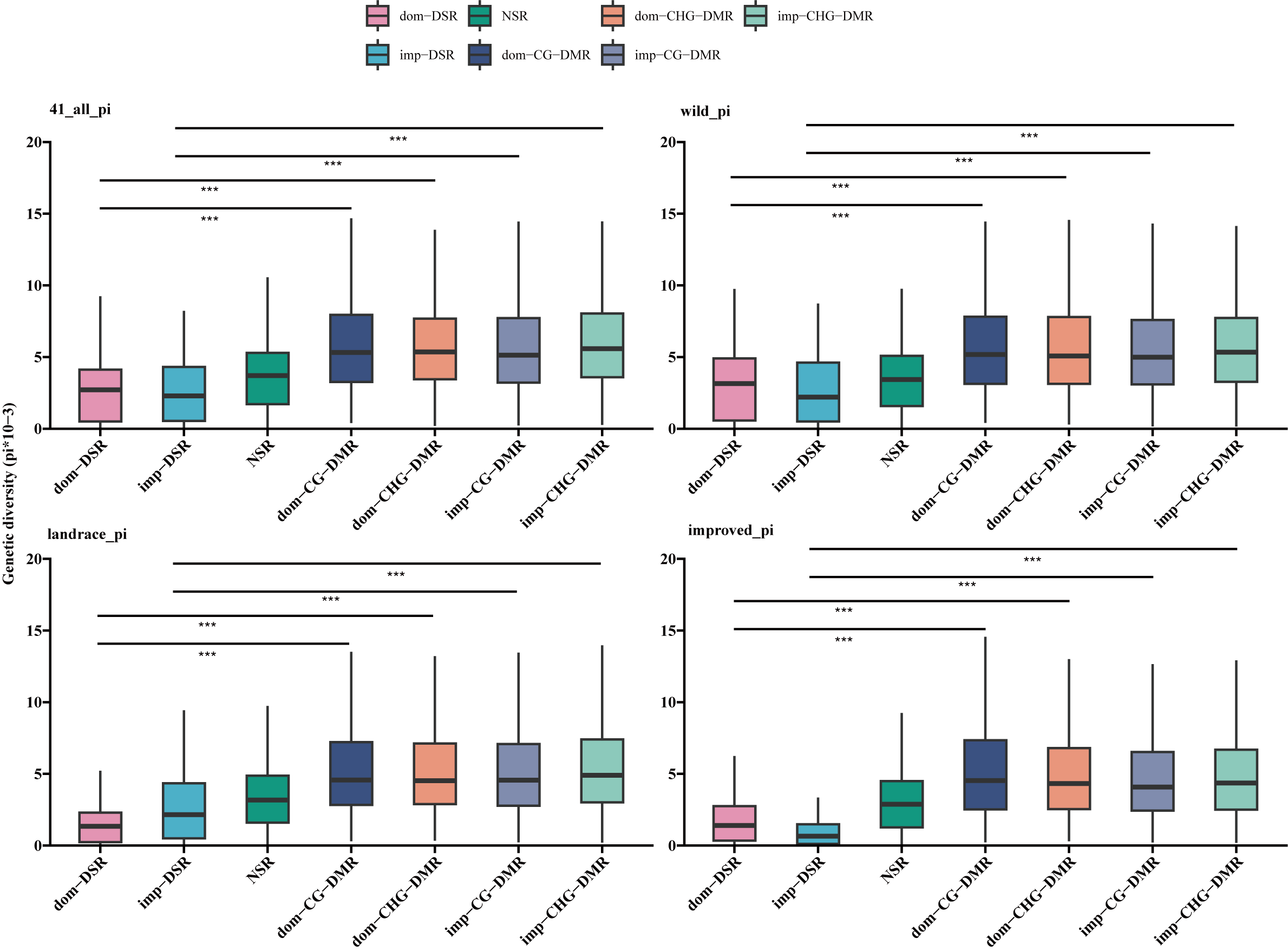


**Fig. S14**: Genetic diversity comparisons between DMRs, DSRs, and NSRs for all pears and the wild, landrace, and improved populations (**P* < 0.05; ***P* < 0.01; ****P* < 0.001, two-tailed paired Student’s *t*-test).


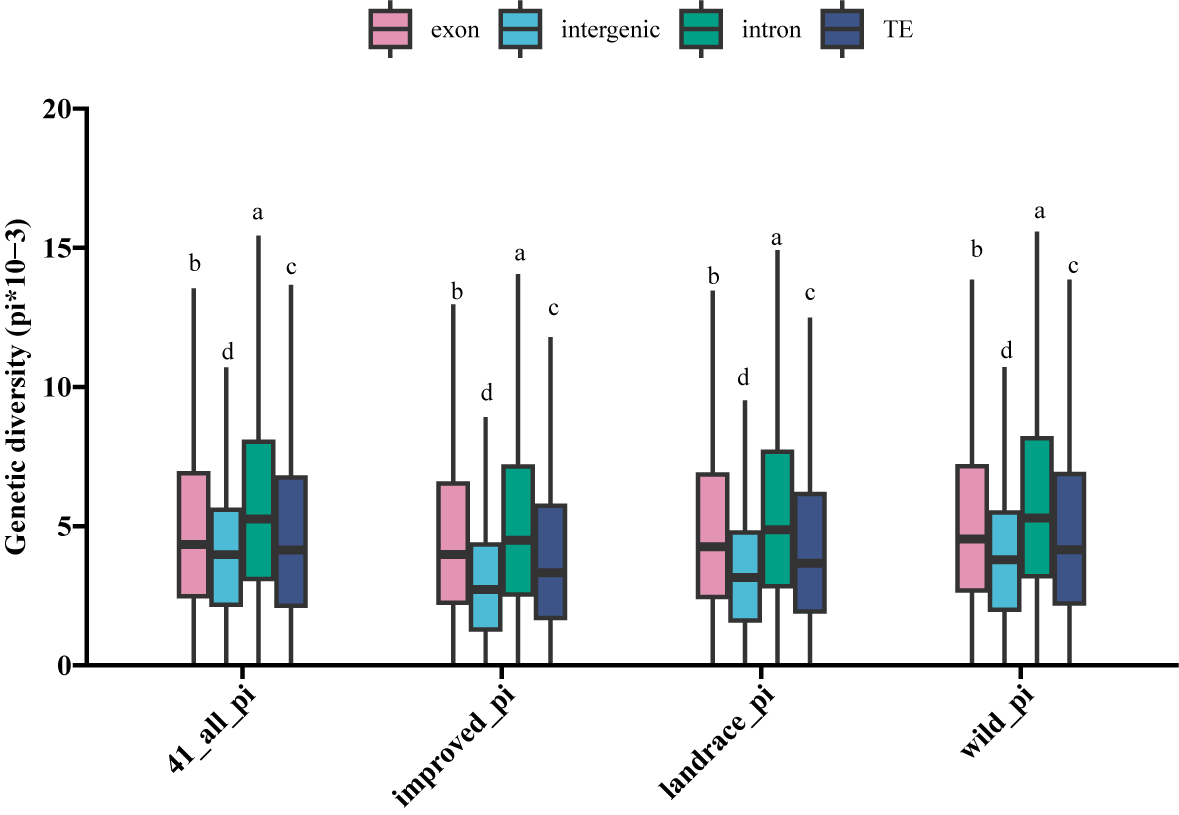


**Fig. S15**: The genetic diversity differences in different genomic backgrounds for the wild, landrace, and improved populations and all pears. The four different genomic backgrounds exhibited different degrees of diversity, and the introns had highest levels of genetic diversity.


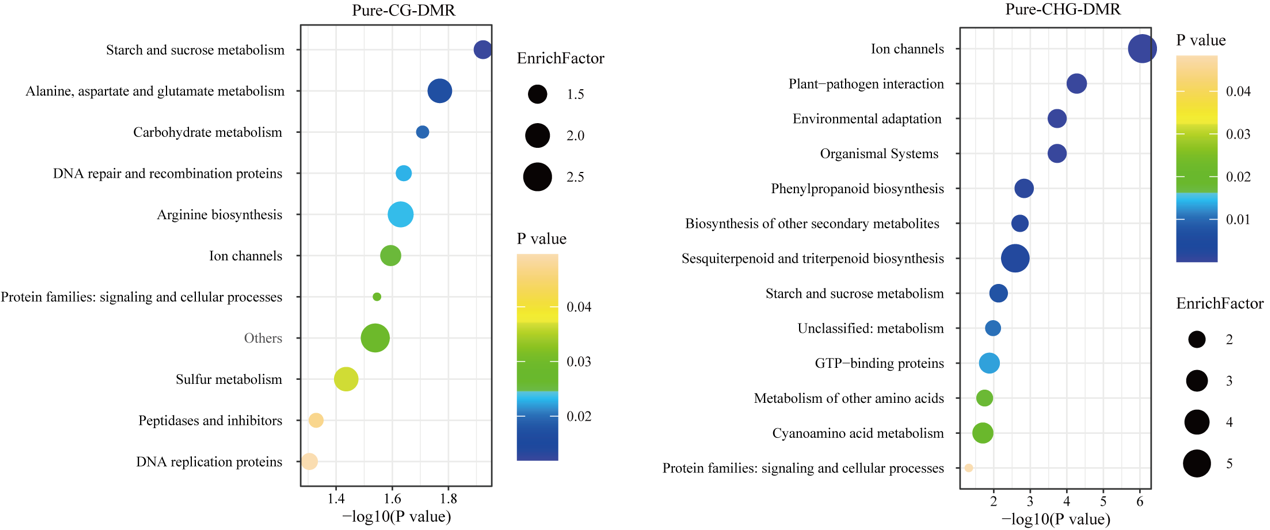


**Fig. S16**: KEGG enrichment analysis of genes associated with “Pure-CG-DMRs” (a) and “Pure-CHG-DMRs” (b).


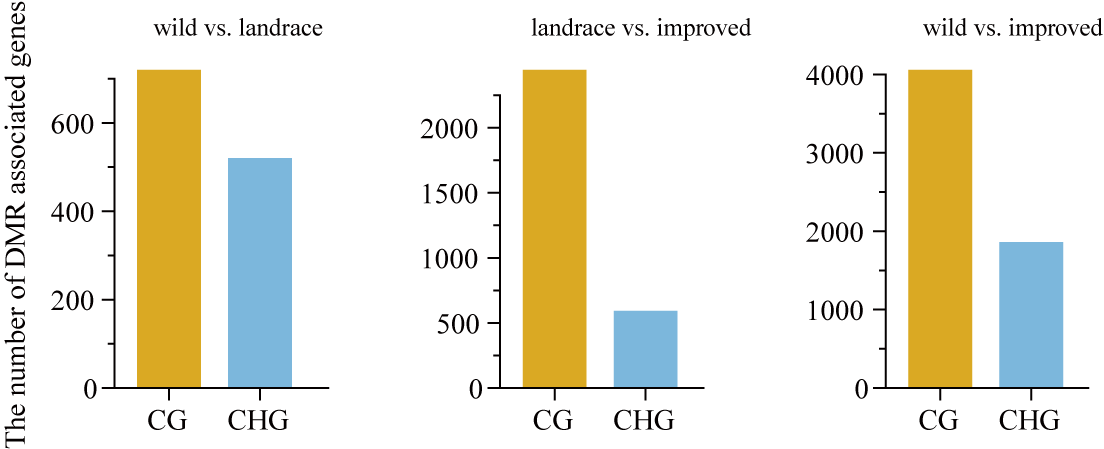


**Fig. S17**: The number of genes found near DMRs in wild vs. landrace, landrace vs. improved, and wild vs. improved comparisons.


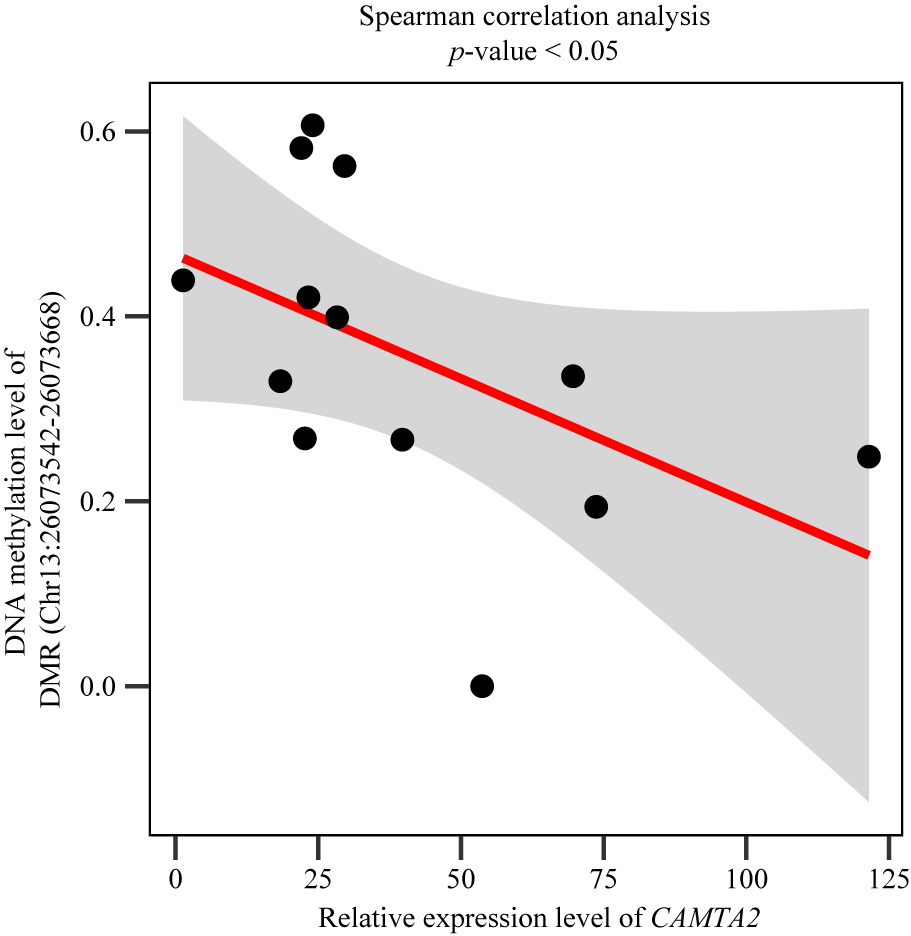


**Fig. S18**: The correlation analysis between DNA methylation level of DMRs (Chr13:26073542-26073668) and expression level of *CAMTA2*.


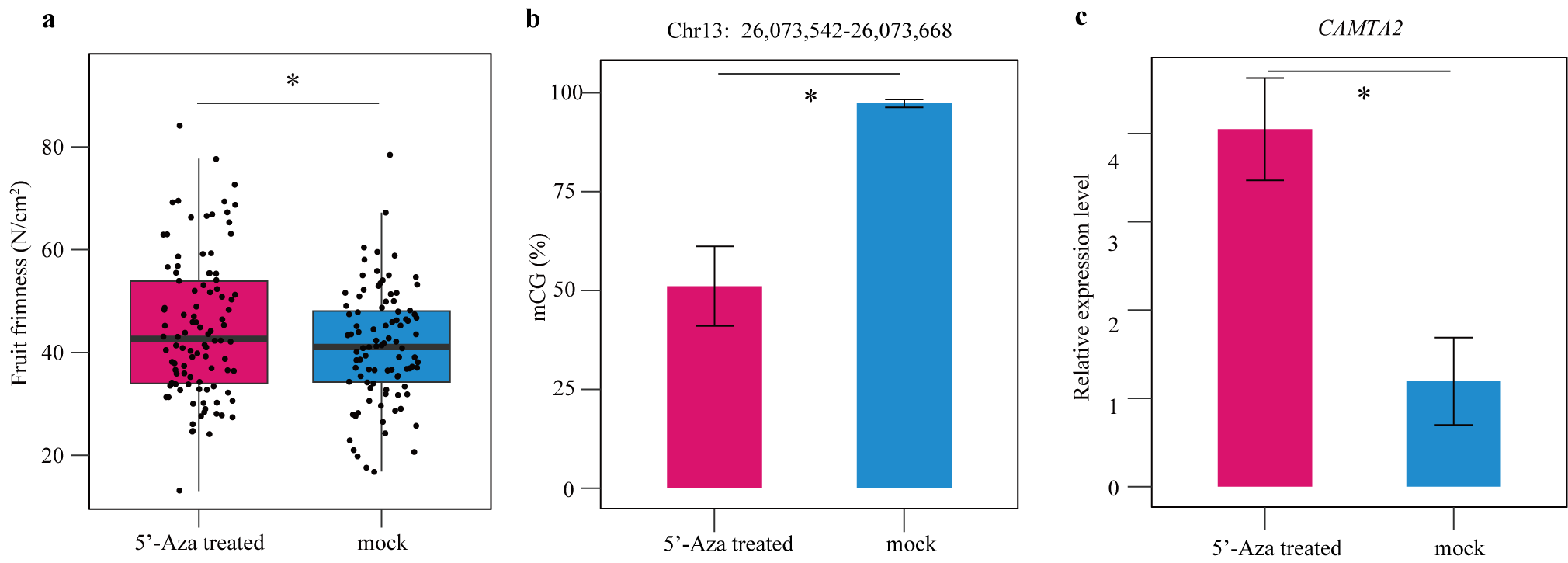


**Fig. S19**: 5’-Aza, and ddH_2_O (mock) were injected into pear fruit. (a) Comparison of fruit firmness between 5’-Aza treated samples and mock. (b) Comparison of gene expression levels of CG methylation level (mCG) in candidate DMR (Chr13: 26,073,542-26,073,668) between 5’-Aza treated samples and mock. (c) Comparison of gene expression levels of *CAMTA2* between 5’-Aza treated samples and mock (**P* < 0.05, two-tailed paired Student’s *t*-test).


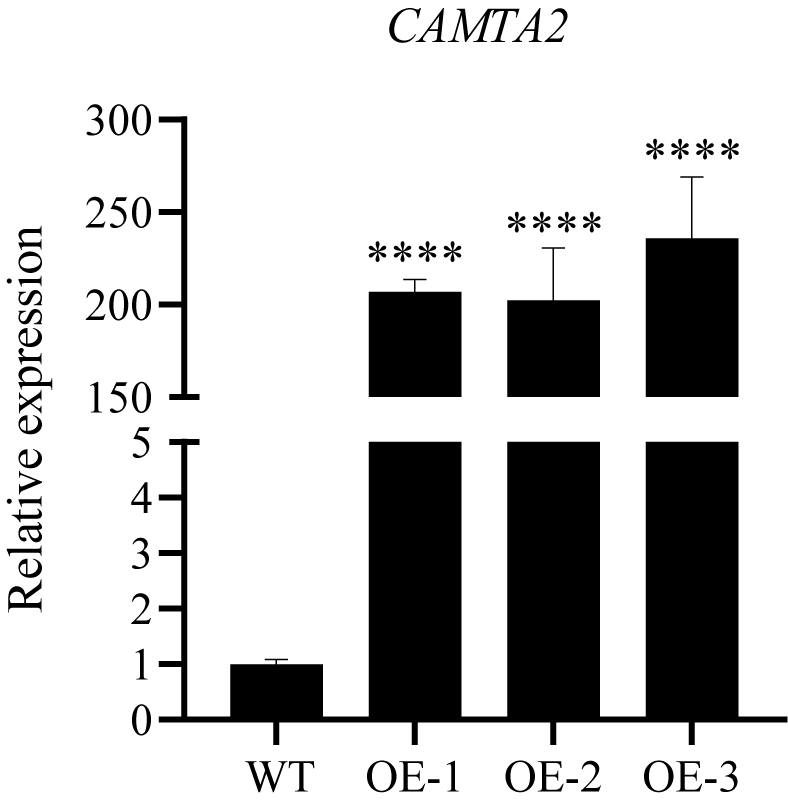


**Fig. S20**: The expression levels of *CAMTA2* in callus tissues of wild-type (WT) and three *CAMTA2* overexpression lines (**P* < 0.05; ***P* < 0.01; ****P* < 0.001; *****P* < 0.0001; NS = not significant, two-tailed paired Student’s *t*-test).


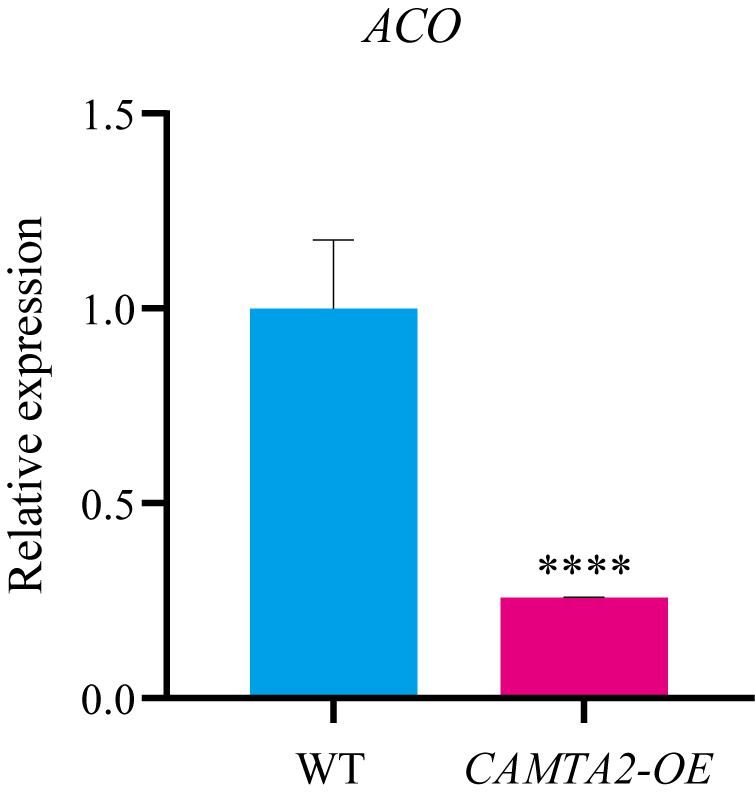


**Fig. S21**: The expression level of the *ACO* gene in wild-type (WT) and *CAMTA2*-overexpressing pear callus tissue (**P* < 0.05; ***P* < 0.01; ****P* < 0.001; *****P* < 0.0001; NS = not significant, two-tailed paired Student’s *t*-test).


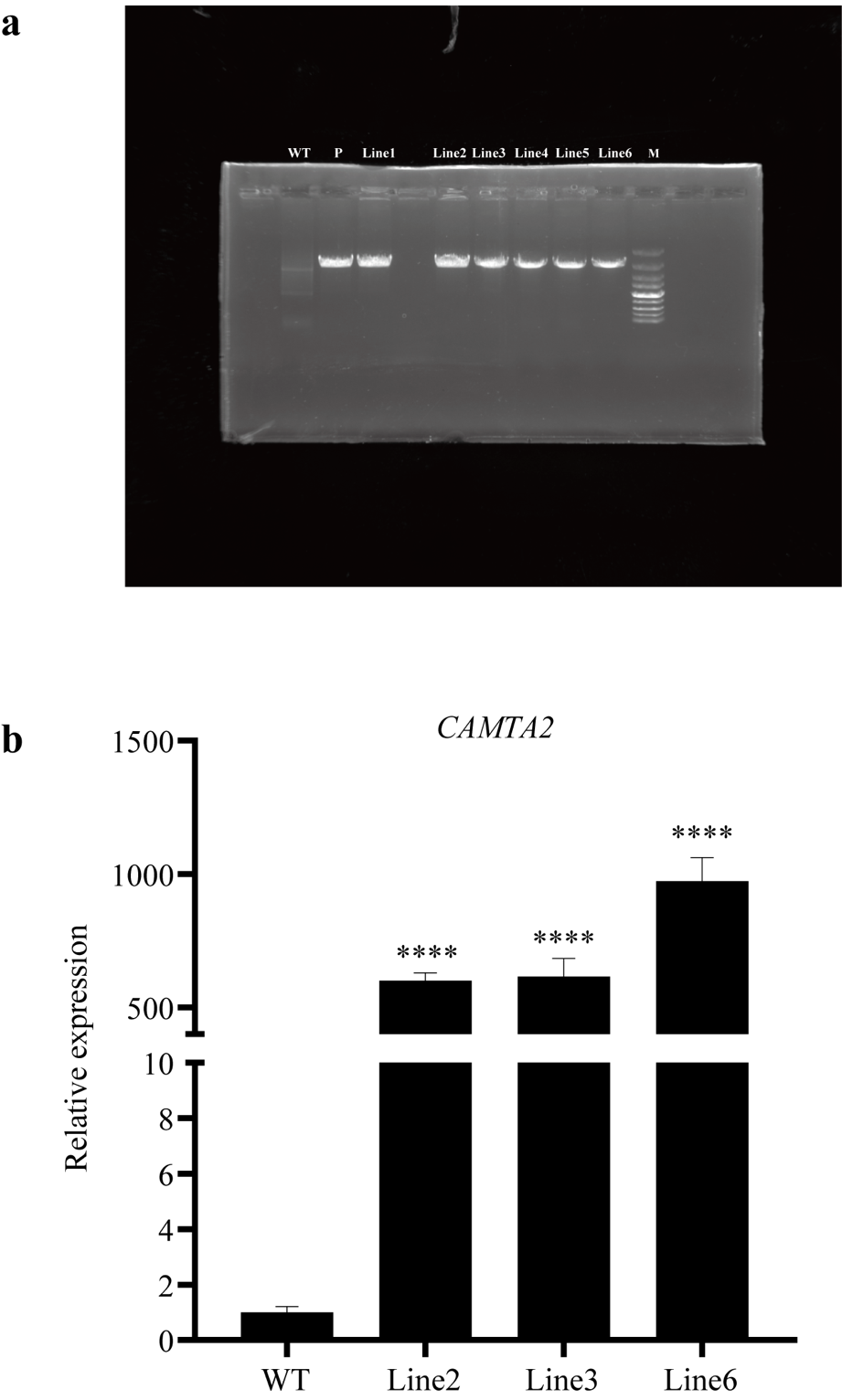


**Fig. S22:** Identification of *CAMTA2* in transgenic T_1_-generation tomato plants. (a) detection of transgenic plants carrying *CAMTA2* using PCR amplification. WT represents wild-type plants (the cultivar ‘Micro-Tom’; the negative control), P represents the plasmid used for transformation (the positive control), and Lines 1-6 are transgenic tomato plants. (b) Quantitative RT-PCR was used to determine the relative expression of *CAMTA2* in plants of the three transgenic lines (**P* < 0.05; ***P* < 0.01; ****P* < 0.001; *****P* < 0.0001; NS = not significant, two-tailed paired Student’s *t*-test).


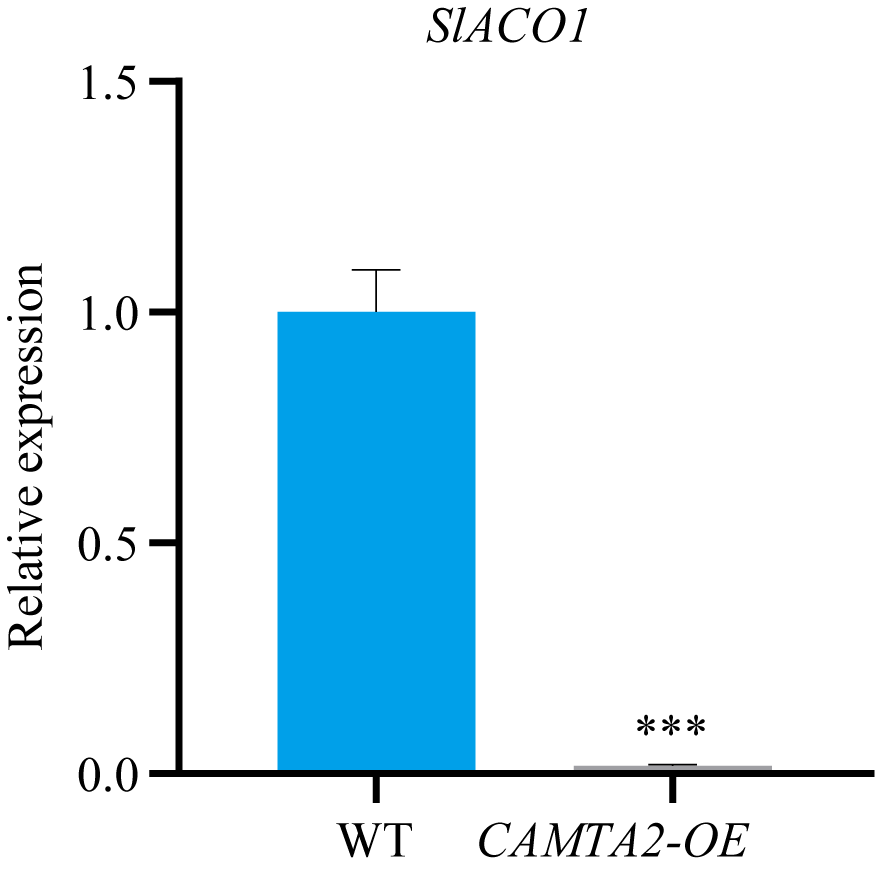


**Fig. S23**: Relative expression levels of the *SlACO* gene in wild-type (WT; the cultivar ‘Micro-Tom’) and transgenic *CAMTA2*-overexpressing (*CAMTA2-OE*) tomato plants **P* < 0.05; ***P* < 0.01; ****P* < 0.001; *****P* < 0.0001; NS = not significant, two-tailed paired Student’s *t*-test).


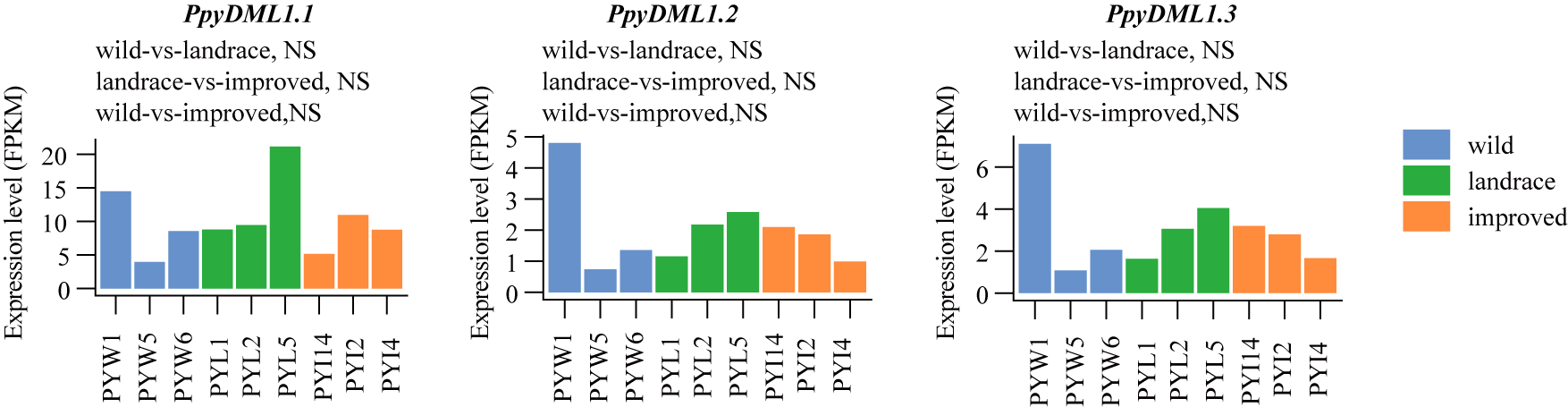


**Fig. S24**. The expression level of *PpyDML1.1, PpyDML1.2* and *PpyDML1.3* genes in leaf of three wild, three landrace and three improved pears.


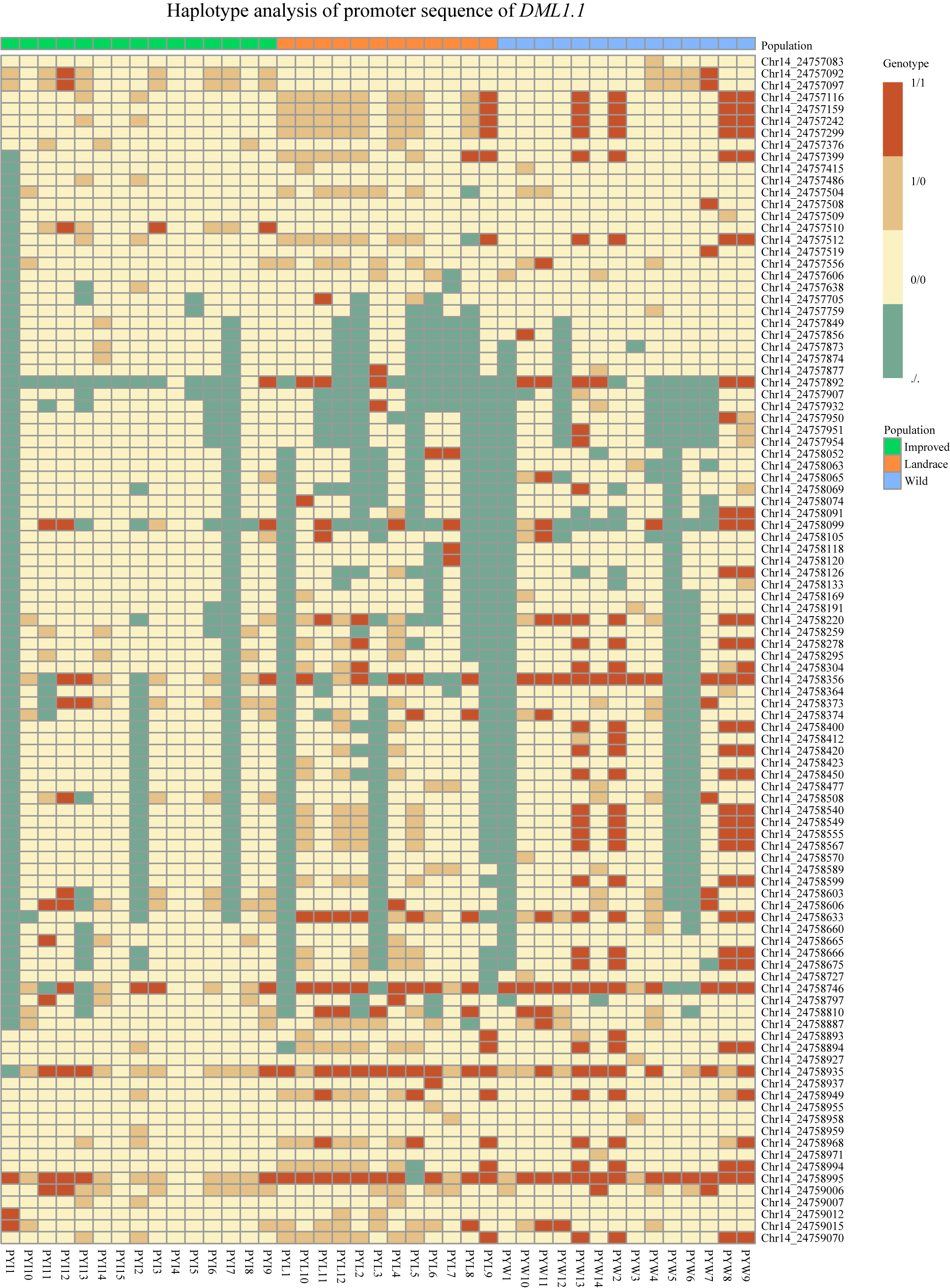


**Fig. S25:** The cis-acting elements analysis and haplotype analysis of promoter sequences of *PpyDML1.1* genes.


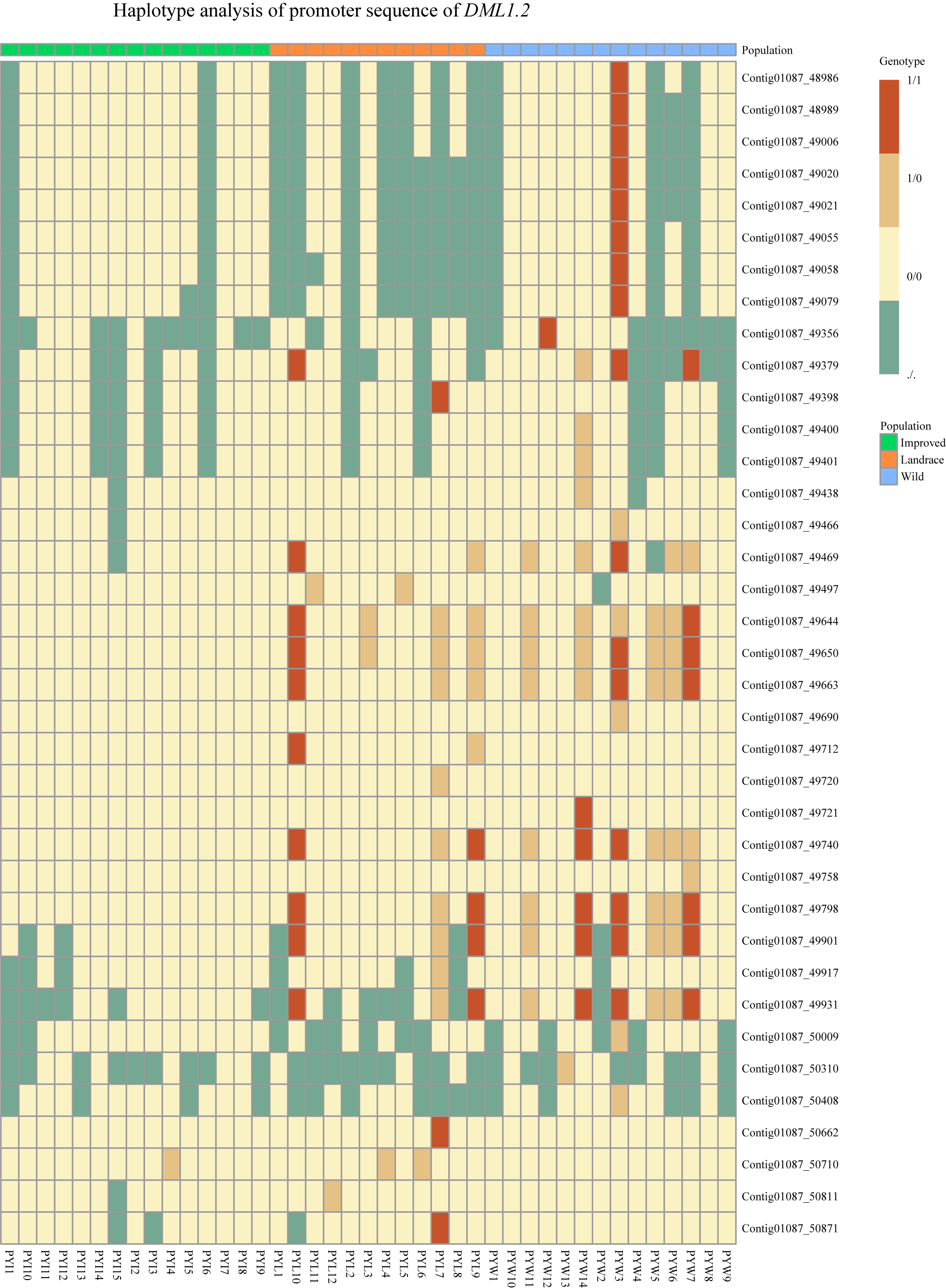


**Fig. S26:** The cis-acting elements analysis and haplotype analysis of promoter sequences of *PpyDML1.2* genes.


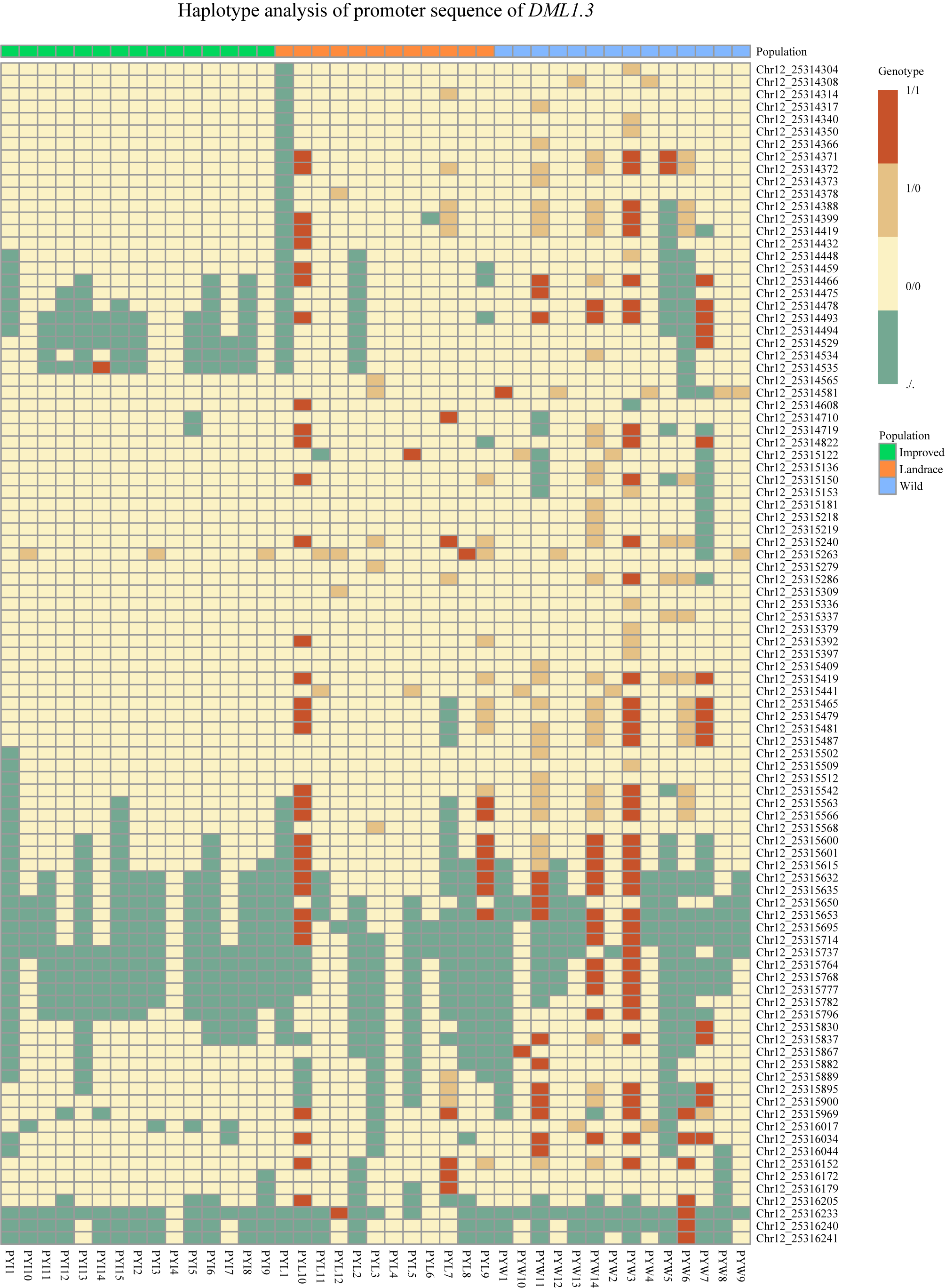


**Fig. S27:** The cis-acting elements analysis and haplotype analysis of promoter sequences of *PpyDML1.3* genes.
