# Supplementary material for: Increased DNA methylation contributes to the early ripening of pear fruits during domestication and improvement
Source: Genome Biol. 2024 Apr 5;25:87. doi: 10.1186/s13059-024-03220-y (PMC10996114; doi:10.1186/s13059-024-03220-y)
Supplement: Supplementary file 3 — Additional file 3. Review history. [file 13059_2024_3220_MOESM3_ESM.docx]

Review History

First round of review

Reviewer 1

Were you able to assess all statistics in the manuscript, including the appropriateness of statistical tests used?

No, I do not feel adequately qualified to assess the statistics.

Comments to author:

The manuscript titled "Increased DNA methylation contributed to early ripening of pear fruits during domestication and improvement" by Song et al. studied the variations in DNA methylation during pear domestication and improvement using 41 pear accessions. They found a global increase in DNA methylation during pear domestication and improvement and identified thousands of DMRs. This global increase in DNA methylation is correlated with down-expression of DML1 (encoding DNA demethylase) under human selection. The majority of DMRs are not associated with SNPs. They also examined the correlation between gene expression and DNA methylation levels. Moreover, they studied the function of a hyper-DMR-associated gene CAMTA2 by overexpressing CAMTA2 in tomato and pear callus.

The authors integrated the data from DNA methylation and transcriptome, which may serve as valuable sources for future studies. Below I list several suggestions to enhance the overall quality of the manuscript.

Major comments:

1. Most of the main figures lack statistically significant tests. Statistical tests are essential.

2. The authors collected fruit flesh samples for DNA methylation variation testing. Considering previous reports indicating that wild pears possess a higher stone cell content in their flesh compared to cultivated accessions, the DNA methylation changes, especially in the CHG context, might largely reflect differences in cell types.

3. For the meQTLs analysis:

The pear genome is ~500 MB with 17 chromosomes, and each chromosome is about 30 Mb in length. The authors used 10 Mb for defining local SNPs, which seems not appropriate. Moreover, given the large diversity of small number of accessions (41) included in this study, it might be challenging to draw conclusive results from the meQTL analysis. Expanding the analysis to include more populations would be better.

4. The authors tried to establish several links in their study including 1) the link between DNA methylation level change and expression level of CAMTA2; 2) the link between DNA methylation variations and fruit ripening. They need to provide more experimental evidence to support their conclusion.

First, Figure 6C shows an increase in methylation levels on the 10th exon of CAMTA2, leading to the repression of its expression as the authors stated in Line 295. However, the correlation observed between DNA methylation variations and expression changes of CAMTA2 needs further experimental evidence. Moreover, could the authors provide more insights into how these methylation changes on this locus influence gene expression? Alternatively, citing evidence from other studies that demonstrate similar transcriptional regulation through DNA methylation on exons, especially last exons, could strengthen this conclusion.

Second, 5-Aza inhibits global DNA methylation to alter expression of many genes. However, some of these gene expression changes might not directly result from the methylation alterations induced by 5-Aza. Additionally, tissue culture could induce substantial changes in DNA methylation and transcription, particularly in callus, when compared to normal plants. These limitations of using 5-Aza and callus compromised the conclusion in this study. In addition, it would be better to show the expression levels and DNA methylation patterns of CAMTA2 in wild species of pear in comparison with cultivated and improved accessions.

5. The authors should provide more detailed information, especially for the data analysis, in the Methods part. For example, in the section of "DMR identification" (Line 477-482), it would be better to include information about the length distribution of DMRs for the comparisons and the specific filtering criteria and method employed. Additionally, since metilene was trained using human data for CG methylation testing, a new training model might be necessary to ensure the accuracy of CHG and CHH DMR identification. Did the authors modify the pipeline to ensure validity of CHG and CHH DMRs?

6. The manuscript contains many grammatical errors. English editing is necessary to improve its clarity.

Minor comments:

1. The authors did not mention the heterozygosity of the wild, landrace and improved accessions of pear used in the study. It would be difficult to access the effects of heterozygosity on the analysis of DNA methylation.

2. Could the authors explain why certain pear accessions, like P585_M2 and P224_M2, were detected with significantly fewer cytosines, on the order of magnitude less?

3. Line 115 is confusing. Helitron and DNA transposons typically may not undergo transcription. Furthermore, could the authors elaborate on the factors contributing to the negative correlation between their density and DNA methylation?

4. Line 121: the conclusion of high CHH context in woody may be too hasty based on the data from two species. Moreover, the high context of CHH in pear suggests its importance, but the authors exclude CHH in their further analysis.

5. Line 143, I could not find expression data for other genes related to methylation. Are the three demethylation genes consistently expressed across multiple tissues? Could the authors elucidate the factors contributing to the downregulation of their expression?

6. Line 162, how much percentage of variation is presented by the PC1/2 of Figures 2a and 2b? Why are there three wild accessions separated far from others? Could these accessions affect final analysis?

7. Line 177, Figure 2f shows that most DMRs are the intergenic. How prevalent are DMRs in the upstream and downstream regions of genes?

8. Line 180, it would be better if the authors could provide insights into why the majority of DMRs are not shared between domestication and improvement. Is it possible that the currently cultivated or improved accessions do not have a direct lineage from wild pears, or might there be other factors affecting the identification of DMRs?

9. Line 230, The limited number of local SNPs relative to dom-DMRs (Figure 4a) appears somewhat implausible. Furthermore, the observation that most imp-CHG-DMRs correspond to more than four SNPs requires further clarification.

10. Line 457, what are the parameters for SNPs filtering?

11. Line 460, the rationale behind adjusting the calculation for pi based on the commonly used 100 kb bins with a 10 kb step should be explained.

12. Line 465, additional details about the analysis of RNA-seq would be beneficial

13. Line 489, what is length of LD for filtering SNPs.

Reviewer 2

Were you able to assess all statistics in the manuscript, including the appropriateness of statistical tests used?

Yes, and I have assessed the statistics in my report.

Comments to author:

The manuscript perspective is interesting although addresses a good contribution to clarify the relationship of human collection and selection to early ripening trait in pear fruits and its correlation to DNA methylation patterns from the wild, landraces and improved cultivars. I recommend accepting this paper but with following minor revisions.

1. 136-137, results indicate to down-regulation of DNA demethylase in pear ripening, while in tomato ripening was up-regulation of DNA demethylase genes (https://doi.org/10.1093/jxb/eraa003 ), while the authors used tomato to verify DNA methylation related genes in another part, may be compatibility or contradiction according to the fruits type. So, can the authors explain the variant in the discussion.

2. The concept of DNA methylation levels and genetic diversity need to be more considered because during the bisulfite treatment, insufficient conversion and DNA degradation can also result in a loss of sequencing diversity and specificity. (for more info https://doi.org/10.1038/s41467-021-26278-9 )

3. Is better if the authors select another scale color of figure (Fig.4 a) to be clearer. While in Fig. 6 the color scale needs to be added.

4. Line 360 - 363, DNA demethylation regulates, The authors need to support these information (See these article, it can be giving the authors more information "Regulation of Leaf Longevity by DML3-Mediated DNA Demethylation" https://doi.org/10.1016/j.molp.2020.06.006

5. Line 388-391: The comparison between prior research about the impact of DNA methylation on gene expression is confusing, so how could you refer to this as a contradiction? Furthermore, the concluding comment distracts the reader, therefore it is recommended that it be reconsidered.

6. There is some writing deficit so this manuscript should undergo prof-language editing before it can be published.

Authors Response

Point-by-point responses to the reviewers’ comments:

Reviewer #1:

The manuscript titled "Increased DNA methylation contributed to early ripening of pear fruits during domestication and improvement" by Song et al. studied the variations in DNA methylation during pear domestication and improvement using 41 pear accessions. They found a global increase in DNA methylation during pear domestication and improvement and identified thousands of DMRs. This global increase in DNA methylation is correlated with down-expression of DML1 (encoding DNA demethylase) under human selection. The majority of DMRs are not associated with SNPs. They also examined the correlation between gene expression and DNA methylation levels. Moreover, they studied the function of a hyper-DMR-associated gene CAMTA2 by overexpressing CAMTA2 in tomato and pear callus.
The authors integrated the data from DNA methylation and transcriptome, which may serve as valuable sources for future studies. Below I list several suggestions to enhance the overall quality of the manuscript.
**Authors’ response:** We greatly appreciate the review’s positive comments and constructive suggestion for our manuscript, which was helpful for improving the quality of manuscript. We had revised the manuscript according to the reviewer’s suggestions point by point as follows.

Major comments:
1. Most of the main figures lack statistically significant tests. Statistical tests are essential.
**Authors’ response:** Thanks for your nice suggestion. We had added statistical tests for the analysis in this study. Please see the revision in Fig. 1-7 and Additional file 2: Fig. S1-S27.

2. The authors collected fruit flesh samples for DNA methylation variation testing. Considering previous reports indicating that wild pears possess a higher stone cell content in their flesh compared to cultivated accessions, the DNA methylation changes, especially in the CHG context, might largely reflect differences in cell types.
**Authors’ response:** We greatly appreciate review’s nice suggestion. It’s true the wild pears possess a higher stone cell content in their flesh compared to cultivated accessions. However, the stone cell is mainly accumulated in early fruit development stages in pear (21-49 days after full blooming) (Xue et al. 2019). As we known, the secondary cell wall thickening and lignin deposition are key steps during the development from parenchyma cells to stone cells. With the thickening of the secondary cell wall, the cell contents gradually shrink to the cell center. Eventually, the cell contents disappear, and the entire cell is completely composed of secondary cell wall structures (Choi and Lee, 2013; Jin et al. 2013; Zhao et al., 2013). In our study, we collected mature pear fruit for DNA methylation variation testing, the stone cell is dead cell and did not contain nuclear DNA at this stage, which will not affect DNA methylation analysis of fruit flesh at mature stage. So, the DNA methylation changes in fruit flesh at mature stage would not affect by differences in cell types.

Reference:

Xue, C., Yao, J.-L., Qin, M.-F., Zhang, M.-Y., Allan, A.C., Wang, D.-F. and Wu, J. (2019), PbrmiR397a regulates lignification during stone cell development in pear fruit. Plant Biotechnol J, 17: 103-117. <https://doi.org/10.1111/pbi.12950>

Choi J H, Lee S H. Distribution of stone cell in Asian, Chinese, and European pear fruit and its morphological changes[J]. Journal of Applied Botany and Food Quality, 2013, 86(1). https://doi.org/10.5073/JABFQ.2013.086.025

Jin Q, Yan C, Qiu J, et al. Structural characterization and deposition of stone cell lignin in Dangshan Su pear[J]. Scientia Horticulturae, 2013, 155: 123-130. https://doi.org/10.1016/j.scienta.2013.03.020

Zhao S G, Zhang J G, Zhao Y P, et al. New discoveries of stone cell differentiation in fruitlets of ‘Yali’pears (Pyrus bretschneideri Rehd.)[J]. J. Food Agric. Enviro, 2013, 11: 937-942.

3. For the meQTLs analysis:
The pear genome is ~500 MB with 17 chromosomes, and each chromosome is about 30 Mb in length. The authors used 10 Mb for defining local SNPs, which seems not appropriate. Moreover, given the large diversity of small number of accessions (41) included in this study, it might be challenging to draw conclusive results from the meQTL analysis. Expanding the analysis to include more populations would be better.
**Authors’ response:** We greatly appreciate your constructive suggestion.

(1) Thanks for your constructive suggestion. We totally agree with reviewer’s opinion. In this revision, we changed the parameter and used 1Mb for defining local SNPs (Fig. 4d) as reference (Guo et al. 2023). Finally, a total of 14 (67% of the total) and 13 (72% of the total) of the local meQTLs were identified as dom-CG-DMRs and dom-CHG-DMRs, respectively. Moreover, a total of 80 (58% of the total) and 34 (59% of the total) of the local meQTLs were identified as imp-CG-DMRs and imp-CHG-DMRs, respectively. These results indicated that dom-DMRs and imp-DMRs tend to be controlled by local meQTLs. In the revised manuscript, we identified more proportion local meQTLs than that of our previous manuscript. This result is consistent with the previous reported in maize that DNA methylation mainly associated with local meQTLs (Shen et al. 2018). We have revised the relevant figure and descriptions in the manuscript. Please see lines 218-227.

(2) Indeed, we agree with reviewer’s opinion that small number of accessions used in our study. Firstly, in view of the collection of a big panel of accession for perennial fruit species is difficult, we used 41 pear accession to do meQTLs analysis. The population number is similar with previous research in soybean (Schmitz et al. 2013; Shen et al. 2018), maize (Steven et al. 2013), and tomato (Guo et al. 2023). For example, Shen used 45 soybean accessions to explore the relationship between SNPs and DNA methylation level of DMRs (Shen et al. 2018). In maize, 51 maizes were used to identified significantly associated SNPs with DMRs (Steven et al. 2013). All these references supported the reliable results could be achieved using similar numbers of population.

Secondly, in our study, we performed two biology replicates to increase the accuracy of meQTLs, while the previous reference (Shen et al. 2018; Xu et al. 2019) only use one biology replicates.

Thirdly, to minimize false positive SNPs, we used a relatively strict threshold value to filter candidate SNPs. A Bonferroni-corrected P < 1.78 * 10^-9^ (0.01/N, N=5,618,948) was used to determine significant associations.

Therefore, the conclusive results draw from the meQTLs analysis in our study could be reliable.

Reference:

Guo, H., Cao, P., Wang, C. et al. Population analysis reveals the roles of DNA methylation in tomato domestication and metabolic diversity. Sci. China Life Sci. 66, 1888–1902 (2023). https://doi.org/10.1007/s11427-022-2299-5

Shen, Y., Zhang, J., Liu, Y. et al. DNA methylation footprints during soybean domestication and improvement. Genome Biol 19, 128 (2018). https://doi.org/10.1186/s13059-018-1516-z

Schmitz R J, He Y, Valdés-López O, et al. Epigenome-wide inheritance of cytosine methylation variants in a recombinant inbred population[J]. Genome research, 2013, 23(10): 1663-1674. https://doi.org/10.1101/gr.152538.112

Eichten, S. R., Briskine, R., Song, J., Li, Q., Swanson-Wagner, R., Hermanson, P. J., Waters, A. J., Starr, E., West, P. T., Tiffin, P., Myers, C. L., Vaughn, M. W., & Springer, N. M. (2013). Epigenetic and genetic influences on DNA methylation variation in maize populations. The Plant cell, 25(8), 2783–2797. <https://doi.org/10.1105/tpc.113.114793>

Xu, J., Chen, G., Hermanson, P.J. et al. Population-level analysis reveals the widespread occurrence and phenotypic consequence of DNA methylation variation not tagged by genetic variation in maize. Genome Biol 20, 243 (2019). https://doi.org/10.1186/s13059-019-1859-0

4. The authors tried to establish several links in their study including 1) the link between DNA methylation level change and expression level of CAMTA2; 2) the link between DNA methylation variations and fruit ripening. They need to provide more experimental evidence to support their conclusion.

First, Figure 6C shows an increase in methylation levels on the 10th exon of CAMTA2, leading to the repression of its expression as the authors stated in Line 295. However, the correlation observed between DNA methylation variations and expression changes of CAMTA2 needs further experimental evidence. Moreover, could the authors provide more insights into how these methylation changes on this locus influence gene expression? Alternatively, citing evidence from other studies that demonstrate similar transcriptional regulation through DNA methylation on exons, especially last exons, could strengthen this conclusion.

**Authors’ response:** We greatly appreciate reviewer’s constructive suggestion.

(1) To verify the correlation between DNA methylation variations and expression changes of CAMTA2, we performed qRT-PCR to measure the expression level of CAMTA2 in 13 pear accessions. The correlation analysis showed that expression level of CAMTA2 exhibited significant negative correlation (P-value < 0.05) with DNA methylation level in different accessions (Figure R1). We added the relevant figure and description in the revised manuscript. Please see lines 277-281 and Additional file 2: Fig. S18.

Figure R1. Correlation analysis between DNA methylation level of DMRs (Chr13:26073542-26073668) and the expression level of CAMTA2.

(2) Additionally, sorry for our unclear description. Actually, the DMR in our study is located on the middle exon (gene body) rather than the last exon. It is not unusual about that these methylation changes on gene body influence gene expression (Chuang et al. 2012; Martino et al. 2015; Moore et al. 2018; Luo et al. 2018; Li et al. 2018; Wei et al. 2023; Li et al. 2023). Previous studies have reported that DNA methylation in the gene body might influence gene expression by promoting chromatin densification (Deaton et al. 2011) and interacting with functional elements in transcribed regions (Yang et al. 2014; Wang et al. 2022). To make it clear, we added corresponding description in discussion, please see lines 422-425.

Reference:

Chuang T J, Chen F C, Chen Y Z. Position-dependent correlations between DNA methylation and the evolutionary rates of mammalian coding exons[J]. Proceedings of the National Academy of Sciences, 2012, 109(39): 15841-15846. <https://doi.org/10.1073/pnas.1208214109>

Martino D, Saffery R. Characteristics of DNA methylation and gene expression in regulatory features on the Infinium 450k Beadchip[J]. BioRxiv, 2015: 032862

Moore, L., Le, T. & Fan, G. DNA Methylation and Its Basic Function. Neuropsychopharmacol 38, 23–38 (2013). <https://doi.org/10.1038/npp.2012.112>

Luo R, Bai C, Yang L, et al. DNA methylation subpatterns at distinct regulatory regions in human early embryos[J]. Royal Society Open Biology, 2018, 8(10): 180131. https://doi.org/10.1098/rsob.180131

Shengli Li, Jiwei Zhang, Shenglin Huang, Xianghuo He, Genome-wide analysis reveals that exon methylation facilitates its selective usage in the human transcriptome, Briefings in Bioinformatics, Volume 19, Issue 5, September 2018, Pages 754–764, https://doi.org/10.1093/bib/bbx019

WEI W, JIANG F, LIU H, et al. The PcHY5 methylation is associated with anthocyanin biosynthesis and transport in ‘Max red Bartlett’and ‘Bartlett’pears[J]. Journal of Integrative Agriculture, 2023. https://doi.org/10.1016/j.jia.2023.07.017

Li, J., Han, F., Yuan, T. et al. The methylation landscape of giga-genome and the epigenetic timer of age in Chinese pine. Nat Commun 14, 1947 (2023). <https://doi.org/10.1038/s41467-023-37684-6>

Deaton AM, Webb S, Kerr ARW, Illingworth RS, Guy J, Andrews R, et al. Cell type-specific DNA methylation at intragenic CpG islands in the immune system. Genome Res. 2011;21(7):1074–86. https://doi.org/10.1101/gr.118703.110

Yang X, Han H, De Carvalho DD, Lay FD, Jones PA, Liang G. Gene body methylation can alter gene expression and is a therapeutic target in cancer. Cancer Cell. 2014;26(4):577–90. <https://doi.org/10.1016/j.ccr.2014.07.028>

Wang Q, Xiong F, Wu G, Liu W, Chen J, Wang B, Chen Y. Gene body methylation in cancer: molecular mechanisms and clinical applications. Clin Epigenetics. 2022 Nov 28;14(1):154. doi: 10.1186/s13148-022-01382-9. PMID: 36443876; PMCID: PMC9706891

Second, 5’-Aza inhibits global DNA methylation to alter expression of many genes. However, some of these gene expression changes might not directly result from the methylation alterations induced by 5’-Aza. Additionally, tissue culture could induce substantial changes in DNA methylation and transcription, particularly in callus, when compared to normal plants. These limitations of using 5’-Aza and callus compromised the conclusion in this study. In addition, it would be better to show the expression levels and DNA methylation patterns of CAMTA2 in wild species of pear in comparison with cultivated and improved accessions.

**Authors’ response:** We greatly appreciate reviewer’s constructive suggestion.

(1) Firstly, we agree with reviewer’s opinion that it is difficult to find a way specifically change DNA methylation of the target gene. So, the 5’-Aza treatment was used to inhibits global DNA methylation to alter expression of many genes, which has been widely used in plant species to study the role of DNA methylation, such as tomato (Zhong et al, 2013), orange (Huang et al. 2018), pear (Liu et al. 2023), etc. To verify the correlation between methylation level and gene expression change in our study, after 5’-Aza treatment, we found that the CG methylation level in candidate DMR (Chr13: 26,073,542-26,073,668) was significantly decreased. Furthermore, we verified CAMTA2 gene expression using qRT-PCR, which significantly up-regulated in 5’-Aza treated samples than mock (Figure R2). This result supported that 5’-Aza treatment increased the CAMTA2 expression by inhibiting CG methylation level on CAMTA2.

(2) Secondly, we agree with the review’s opinion that tissue culture could induce substantial changes in DNA methylation and transcription. To further support our results, we added the experiment of 5’-Aza treatment in pear fruit, the results showed that fruit firmness after five days of 5’-Aza treatment was significantly higher than that of mock (Figure R2a), which indicated 5’-Aza treatment delayed fruit softening. Furthermore, bisulfite sequencing PCR indicated that the CG methylation level in candidate DMR (Chr13: 26,073,542-26,073,668) significantly decreased (Figure R2b). Meanwhile, qRT-PCR showed that CAMTA2 was significantly up-regulated in 5’-Aza treated samples than mock (Figure R2c). Therefore, the fruit treatment and callus treatment showed the similar results that DNA methylation play important roles during fruit ripening of pear by regulating gene expression of CAMTA2. To make it clear, we have added the relevant descriptions in the revised manuscript. Please see lines 286-295 and Additional file 2: Fig. S19.

(3) Thirdly, according to reviewer’s suggestion, we had added the expression levels and DNA methylation patterns of CAMTA2 in wild population in fig. 6c and fig 6d. The results showed that the DNA methylation were continues increased, while the expression levels of CAMTA2 were continues decreased during pear domestication and improvement process, which further supported the correlation between the expression levels and DNA methylation patterns of CAMTA2. We have added the relevant descriptions in the revised manuscript. Please see lines 272-277.

Figure R2. 5’-Aza, and ddH_2_O (mock) were injected into pear fruits. (a) Comparison of fruit firmness between 5’-Aza treated samples and mock. (b) Comparison of CG methylation level (mCG) gene expression levels in candidate DMR (Chr13: 26,073,542-26,073,668) between 5’-Aza treated samples and mock. (c) Comparison of gene expression levels of CAMTA2 between 5’-Aza treated samples and mock (*P < 0.05, two-tailed paired Student’s t-test).

Reference:

Zhong, S., Fei, Z., Chen, Y. R., Zheng, Y., Huang, M., Vrebalov, J., McQuinn, R., Gapper, N., Liu, B., Xiang, J., Shao, Y., & Giovannoni, J. J. (2013). Single-base resolution methylomes of tomato fruit development reveal epigenome modifications associated with ripening. Nature biotechnology, 31(2), 154–159. https://doi.org/10.1038/nbt.2462

Huang H, Liu R, Niu Q, Tang K, Zhang B, Zhang H, Chen K, Zhu J-K, Lang Z: Global increase in DNA methylation during orange fruit development and ripening. Proceedings of the National Academy of Sciences 2019, 116:1430-1436. https://doi.org/10.1073/pnas.1815441116

Liu, H. N., Shu, Q., Lin-Wang, K., Espley, R. V., Allan, A. C., Pei, M. S., Li, X. L., Su, J., & Wu, J. (2023). DNA methylation reprogramming provides insights into light-induced anthocyanin biosynthesis in red pear. Plant science : an international journal of experimental plant biology, 326, 111499. <https://doi.org/10.1016/j.plantsci.2022.111499>

5. The authors should provide more detailed information, especially for the data analysis, in the Methods part. For example, in the section of "DMR identification" (Line 477-482), it would be better to include information about the length distribution of DMRs for the comparisons and the specific filtering criteria and method employed. Additionally, since metilene was trained using human data for CG methylation testing, a new training model might be necessary to ensure the accuracy of CHG and CHH DMR identification. Did the authors modify the pipeline to ensure validity of CHG and CHH DMRs?

**Authors’ response:** We greatly appreciate for the constructive suggestion.

(1) Sorry for the unclear description for data analysis. In this revision, we added more detailed description for the data analysis in methods part, including resequencing analysis, WGBS analysis, DMR identification, length distribution of DMRs, diversity analysis of DMRs, and TE identification, KEGG and GO enrichment analysis, etc. Please see lines 456-662.

(2) Yes, the ‘metilene’ software was trained using human data, but it was often used to identified CG-DMR, CHG-DMR and CHH DMR in many plant species, such as maize (Xu et al. 2020), soybean (Shen et al. 2018), Welwitschia (Wan et al. 2021), tomato (Guo et al. 2023), strawberry (Sammarco et al. 2020) and citrus (Huang et al. 2023). So, we choose the ‘metilene’ software, which is widely used and accepted in plant research. According to these references, we used ‘metilene’ software to identify DMRs developed from reference’s parameters in plant: at least eight cytosine sites in a DMR; the distance between any two adjacent cytosine sites was <300 bp; the average methylation level between the two populations was >25%. Finally, we used a corrected P-value < 0.01 as the cutoff. The detailed descriptions about DMR identification were included in the revised manuscript. Please see lines 543-553.

Reference:

Xu, G., Lyu, J., Li, Q. et al. Evolutionary and functional genomics of DNA methylation in maize domestication and improvement. Nat Commun 11, 5539 (2020). <https://doi.org/10.1038/s41467-020-19333-4>

Shen, Y., Zhang, J., Liu, Y. et al. DNA methylation footprints during soybean domestication and improvement. Genome Biol 19, 128 (2018). <https://doi.org/10.1186/s13059-018-1516-z>

Wan, T., Liu, Z., Leitch, I.J. et al. The Welwitschia genome reveals a unique biology underpinning extreme longevity in deserts. Nat Commun 12, 4247 (2021). https://doi.org/10.1038/s41467-021-24528-4

Guo, H., Cao, P., Wang, C. et al. Population analysis reveals the roles of DNA methylation in tomato domestication and metabolic diversity. Sci. China Life Sci. 66, 1888–1902 (2023). <https://doi.org/10.1007/s11427-022-2299-5>

Sammarco, I., Díez Rodríguez, B., Galanti, D., Nunn, A., Becker, C., Bossdorf, O., Münzbergová, Z. and Latzel, V. (2023), DNA methylation in the wild: epigenetic transgenerational inheritance can mediate adaptation in clones of wild strawberry (Fragaria vesca). New Phytol. https://doi.org/10.1111/nph.19464

Huang B, Wang P, Jin L, et al. Methylome and transcriptome analysis of flowering branches building of Citrus plants induced by drought stress[J]. Gene, 2023, 880: 147595. https://doi.org/10.1016/j.gene.2023.147595

6. The manuscript contains many grammatical errors. English editing is necessary to improve its clarity.
**Authors’ response:** We are so sorry for some mistake in English writing. In this revision, we asked for the help from a native English speaker colleague to polish the language. Wishing the English writing of this revision has been greatly improved and arrived the publication quality requirement for the journal.

Minor comments:
1. The authors did not mention the heterozygosity of the wild, landrace and improved accessions of pear used in the study. It would be difficult to access the effects of heterozygosity on the analysis of DNA methylation.

**Authors’ response:** We greatly appreciate for the constructive suggestion.

(1) Firstly, we estimated the heterozygosity of each pear accessions using plink software, and compared the heterozygosity of the wild, landrace and improved pear. As result, we found that there is no significant difference of heterozygosity in comparison of wild-vs-landrace, landrace-vs-improved, and wild-vs-improved (Figure R3). The low difference of heterozygosity in wild, landrace and improved pear might due to typical self-incompatibility and relatively week selection during pear domestication and improvement process (Wu et al. 2018; Li et al. 2019; Zhang et al. 2021).

Figure R3. The comparison of heterozygosity between wild, landrace and improved pear.

(2) Furthermore, to investigate the relationship between heterozygosity and DNA methylation level in pear, we performed Pearson correlation coefficient analysis between heterozygosity and DNA methylation level of CG, CHG and CHH context in 41 pear accessions. As result, we found that there is not significantly correlation between heterozygosity and DNA methylation level in pear (Figure R4).

Figure R4. The Pearson correlation coefficient analysis between heterozygosity and DNA methylation level of three context (mCG, mCHG, mCHH).

Reference:

Wu, J., Wang, Y., Xu, J. et al. Diversification and independent domestication of Asian and European pears. Genome Biol **19**, 77 (2018). https://doi.org/10.1186/s13059-018-1452-y

Xiaolong Li, Lun Liu, Meiling Ming, Hongju Hu, Mingyue Zhang, Jing Fan, Bobo Song, Shaoling Zhang, Jun Wu, Comparative Transcriptomic Analysis Provides Insight into the Domestication and Improvement of Pear (P. pyrifolia) Fruit, Plant Physiology, Volume 180, Issue 1, May 2019, Pages 435–452, https://doi.org/10.1104/pp.18.01322

Zhang, MY., Xue, C., Hu, H. et al. Genome-wide association studies provide insights into the genetic determination of fruit traits of pear. Nat Commun 12, 1144 (2021). https://doi.org/10.1038/s41467-021-21378-y

2. Could the authors explain why certain pear accessions, like P585_M2 and P224_M2, were detected with significantly fewer cytosines, on the order of magnitude less?

**Authors’ response:** We greatly appreciate the nice suggestion. The library of P585_M2, P224_M2 and P432_M2 were detected with significantly fewer cytosines, which might cause by low sequence depth (P585_M2: 2, P224_M2: 2.7, P432_M2: 3.6). In this revision, to avoid bias from these three samples, we removed these three samples and reanalyzed all data, the correction does not affect the overall result or conclusion of the article. We had updated data analysis and the corresponding revision in Fig. 1-7 and Additional file 2: Fig. S1-S27.

3. Line 115 is confusing. Helitron and DNA transposons typically may not undergo transcription. Furthermore, could the authors elaborate on the factors contributing to the negative correlation between their density and DNA methylation?

**Authors’ response:** We greatly appreciate for the constructive suggestion.

(1) We are agreed to reviewer’s opinion that Helitron and DNA transposons typically may not undergo transcription. In this revision, we deleted the description related “transcription of Helitron and DNA transposons”.

(2) We greatly appreciate reviewer’s suggestion, and try to investigate the factors contributing to the negative correlation between their density and DNA methylation. Originally, we explore the relationship between the density of one type TEs and DNA methylation level of genome window, and found that the negative correlation between their density and DNA methylation. However, the DNA methylation level of a window is determined by multiple types of TEs in this window, so our previous analysis is unreasonable.

So, we reanalyze the data as follows: to investigate the relationship between DNA methylation level and different type TEs, we calculated the distribution of DNA methylation level across the upstream 2k, gene body, and downstream 2k regions of different type TEs (Figure R5). The result showed that all type TEs exhibited higher methylation level, which is consistent with previous reported that DNA methylation play important role in preventing their transposition (He et al. 2022; Xu et al. 2022). In this revision, we changed the relevant figure and description. Please see lines 107-112.

Figure R5. Distribution of DNA methylation levels across the upstream 2 kb, gene body, and downstream 2 kb regions of different TEs.

Reference:

He, L., Huang, H., Bradai, M. et al. DNA methylation-free Arabidopsis reveals crucial roles of DNA methylation in regulating gene expression and development. Nat Commun 13, 1335 (2022). <https://doi.org/10.1038/s41467-022-28940-2>

Xu, Q., Wu, L., Luo, Z. et al. DNA demethylation affects imprinted gene expression in maize endosperm. Genome Biol 23, 77 (2022). https://doi.org/10.1186/s13059-022-02641-x

4. Line 121: the conclusion of high CHH context in woody may be too hasty based on the data from two species. Moreover, the high context of CHH in pear suggests its importance, but the authors exclude CHH in their further analysis.

**Authors’ response:** We greatly appreciate for the constructive suggestion.

(1) We agreed with your suggestion, and it is too hasty to draw the conclusion based on the data from only two species. Therefore, we deleted this sentence in revised manuscript.

(2) Yes, the high context of CHH in pear was found in our study. However, the CHH sites exhibit much low methylation level in pear comparing with CG and CHG (Additional file 2: Fig. S10). This phenomenon was also found in other plant species, maize and tomato (Xu et al. 2020; Guo et al. 2023), and the CHH context was removed in previous studies due to low methylation level. Therefore, we also removed the CHH methylation in further analysis. Please see lines 148-155.

Reference:

Xu, G., Lyu, J., Li, Q. et al. Evolutionary and functional genomics of DNA methylation in maize domestication and improvement. Nat Commun 11, 5539 (2020). <https://doi.org/10.1038/s41467-020-19333-4>

Guo, H., Cao, P., Wang, C. et al. Population analysis reveals the roles of DNA methylation in tomato domestication and metabolic diversity. Sci. China Life Sci. 66, 1888–1902 (2023). <https://doi.org/10.1007/s11427-022-2299-5>

5. Line 143, I could not find expression data for other genes related to methylation. Are the three demethylation genes consistently expressed across multiple tissues? Could the authors elucidate the factors contributing to the downregulation of their expression?

**Authors’ response:** We greatly appreciate for the constructive suggestion.

(1) According to the reviewer’s suggestion, we added the expression analysis of other genes related to methylation and demethylation in pear. For all identified 12 demethylation genes, only three genes (PpyDML1.1, PpyDML1.2, PpyDML1.3) were exhibited continues decreased during pear domestication and improvement (Fig. 1e and Figure R6a). For all identified 14 methylation genes, none of genes were continues up-regulated during pear domestication and improvement (Figure R6b). Therefore, these results showed that the PpyDML1.1, PpyDML1.2, PpyDML1.3 genes might have been involved in the increase of DNA methylation levels during pear domestication and improvement. We have added relevant description and figures in revised manuscript. Please see lines 130-143 and Additional file 2: Fig. S7-S8.

(2) In previous analysis, we only detect demethylation genes in fruit tissue. In this revision, we added leaf of nine pear accessions for ssRNA-seq, including three wild, three landrace and three improved pears. Result showed that there was no significant difference in the expression of these three genes in the leaf tissues between wild, landrace, and improved pear populations (Figure R7), indicating that PpyDML1.1, PpyDML1.2, PpyDML1.3 exhibited tissue specificity expression. The result is consistent with previous reports that DNA methylation exhibited tissue specificity (Lippman et al. 2004; Ma et al. 2013; Zhang et al. 2020). We added the relevant description in the revised manuscript. Please see lines 352-356.

(3) According to previous reference, genetic variations in promoter might affect gene expression (Williams et al. 2007). To explore the potential reasons of the downregulation of DML1, we performed haplotype analysis of prompter sequence of PpyDML1.1, PpyDML1.2, and PpyDML1.3. As results, we found the apparent divergence of genotype in the promoter sequence of PpyDML1.1, PpyDML1.2, and PpyDML1.3 between wild, landrace, and improved pears (Figure R8). These results indicated that the down-regulation of PpyDML1.1, PpyDML1.2, and PpyDML1.3 might be caused by the variation in promoter sequence. Please see lines 356-363.

Figure R6. The expression level of demethylation (a) and methylation genes (b) in wild, landrace and improved pear populations.

Figure R7. The expression level of PpyDML1.1, PpyDML1.2, and PpyDML1.3 genes in leaf of three wild, three landrace and three improved pears.

Figure R8. The cis-acting elements analysis and haplotype analysis of promoter sequences of PpyDML1.1, PpyDML1.2, and PpyDML1.3 genes.

Reference:

Zhang Y, Liu C, Cheng H, Tian S, Liu Y, Wang S, Zhang H, Saqib M, Wei H, Wei Z: DNA methylation and its effects on gene expression during primary to secondary growth in poplar stems. BMC Genomics 2020, 21:498. https://doi.org/10.1186/s12864-020-06902-6

Lippman Z, Gendrel A-V, Black M, Vaughn MW, Dedhia N, Richard McCombie W, Lavine K, Mittal V, May B, Kasschau KD, et al: Role of transposable elements in heterochromatin and epigenetic control. Nature 2004, 430:471-476. https://doi.org/10.1038/nature02651

Ma K, Song Y, Yang X, Zhang Z, Zhang D: Variation in Genomic Methylation in Natural Populations of Chinese White Poplar. PLOS ONE 2013, 8:e63977. https://doi.org/10.1371/journal.pone.0063977

Concha C M, Figueroa N E, Poblete L A, et al. Methyl jasmonate treatment induces changes in fruit ripening by modifying the expression of several ripening genes in Fragaria chiloensis fruit[J]. Plant Physiology and Biochemistry, 2013, 70: 433-444. https://doi.org/10.1016/j.plaphy.2013.06.008

Tao X, Wu Q, Li J, et al. Ethylene biosynthesis and signal transduction are enhanced during accelerated ripening of postharvest tomato treated with exogenous methyl jasmonate[J]. Scientia Horticulturae, 2021, 281: 109965. https://doi.org/10.1016/j.scienta.2021.109965

Vaishnav, E.D., de Boer, C.G., Molinet, J. et al. The evolution, evolvability and engineering of gene regulatory DNA. Nature 603, 455–463 (2022). <https://doi.org/10.1038/s41586-022-04506-6>

Villao-Uzho, L.; Chávez-Navarrete, T.; Pacheco-Coello, R.; Sánchez-Timm, E.; Santos-Ordóñez, E. Plant Promoters: Their Identification, Characterization, and Role in Gene Regulation. Genes 2023, 14, 1226. https://doi.org/10.3390/genes14061226

Williams R B H, Chan E K F, Cowley M J, et al. The influence of genetic variation on gene expression[J]. Genome research, 2007, 17(12): 1707-1716. https://doi.org/10.1101/gr.6981507

6. Line 162, how much percentage of variation is presented by the PC1/2 of Figures 2a and 2b? Why are there three wild accessions separated far from others? Could these accessions affect final analysis?

**Authors’ response:** We greatly appreciate for the constructive suggestion.

(1) According to the suggestion, we had added percentage of variation in fig. 2a and 2b in the revised manuscript.

(2) We detected the three wild accessions separated far from others, and found that these three wild accessions correspond to three samples (P585_M2, P224_M2 and P432_M2) with lower sequence depth. According to minor comment 2 from reviewers, we removed three libraries with lower sequence depth, and re-performed PCA analysis (Fig. 2a). As a result, these three wild accessions were clustered into wild population.

(3) To avoid bias from these three samples, we removed these three libraries and reanalyzed our data. The correction does not affect the overall result or conclusion of the article. Please see the revision in Fig. 1-7 and Additional file 2: Fig. S1-S27.

7. Line 177, Figure 2f shows that most DMRs are the intergenic. How prevalent are DMRs in the upstream and downstream regions of genes?

**Authors’ response:** Thanks for your nice suggestion. According reviewer’s suggestion, we investigated the DMRs distribution on the upstream 2k and downstream 2k regions of genes. Result showed that a total of 14%-23% of DMRs were located in the upstream and downstream regions of genes (Figure R9). We have added the corresponding description and figure in the revised manuscript. Please see lines 174-175.

Figure R9. The distribution of DMRs in the upstream and downstream regions of various genes.

8. Line 180, it would be better if the authors could provide insights into why the majority of DMRs are not shared between domestication and improvement. Is it possible that the currently cultivated or improved accessions do not have a direct lineage from wild pears, or might there be other factors affecting the identification of DMRs?

**Authors’ response:** Thanks for your nice suggestion. In our study, we found that only a small fraction of DMRs were shared between domestication and improvement in pear. This result is similar to selection sweep analysis in that only 3% of selection sweep regions (182 kb) are shared between domestication and improvement (Figure R10), which suggests that different selections have occurred at different stages. We have included the relevant figure and descriptions in the revised manuscript. Please see line 177-179.

Figure R10. Selection sweep analysis throughout pear domestication and improvement processes. (a) The identification of selective sweeps during pear domestication. Each point represents a 10-kb sliding window. If one sliding window with π ratio > 1.509 and F_ST_ > 0.075 is defined as a selective sweep region during domestication. (b) The identification of selective sweeps during pear improvement. Each point represents a 10-kb sliding window. If one sliding window with π ratio > 1.918 and F_ST_ > 0.157 is defined as selective sweep regions during domestication. (c) Overlap of selective sweep region in the wild vs. landrace (domestication process) and landrace vs. improved (improvement process) comparisons.

Reference:

Wu, J., Wang, Y., Xu, J. et al. Diversification and independent domestication of Asian and European pears. Genome Biol **19**, 77 (2018). https://doi.org/10.1186/s13059-018-1452-y

Xiaolong Li, Lun Liu, Meiling Ming, Hongju Hu, Mingyue Zhang, Jing Fan, Bobo Song, Shaoling Zhang, Jun Wu, Comparative Transcriptomic Analysis Provides Insight into the Domestication and Improvement of Pear (P. pyrifolia) Fruit, Plant Physiology, Volume 180, Issue 1, May 2019, Pages 435–452, https://doi.org/10.1104/pp.18.01322

Zhang, MY., Xue, C., Hu, H. et al. Genome-wide association studies provide insights into the genetic determination of fruit traits of pear. Nat Commun 12, 1144 (2021). <https://doi.org/10.1038/s41467-021-21378-y>

9. Line 230, The limited number of local SNPs relative to dom-DMRs (Figure 4a) appears somewhat implausible. Furthermore, the observation that most imp-CHG-DMRs correspond to more than four SNPs requires further clarification.

**Authors’ response:** Thanks for your nice suggestion.

(1) According to the reviewer’s suggestion, we re-performed meQTLs. Finally, A total of 14 (67% of the total) and 13 (72% of the total) of the local meQTLs were identified as dom-CG-DMRs and dom-CHG-DMRs, respectively. Moreover, 80 (58% of the total) and 34 (59% of the total) of the local meQTLs were identified as imp-CG-DMRs and imp-CHG-DMRs, respectively. These results indicated that dom-DMRs and imp-DMRs tend to be controlled by local meQTLs. Please see the revision in lines 218-228.

(2) According to the updated data analysis, we found that most DMRs were only tagged by one SNP (Fig. 4b). We made corresponding revision in this edition. Please see line 229-230.

10. Line 457, what are the parameters for SNPs filtering?

**Authors’ response:** Sorry for the unclear description, we have added the parameters for SNPs filtering in revised manuscript: missing rate ≤ 70% and MAF ≥ 0.05. Please see line 490.

11. Line 460, the rationale behind adjusting the calculation for pi based on the commonly used 100 kb bins with a 10 kb step should be explained.

**Authors’ response:** Thanks for your nice suggestion. In view of pear is a self-incompatibility species with high heterozygosity, and the previous studies have reported that the genome of pear hold much shorter LD decay distances (211 bp) (Wu et al. 2018) than those reported for annual crops, such as soybean (~150 kb) (Lam et al. 2010) and rice (~123 kb) (Xu et al. 2012). Also, many previous studies have used 10 kb as window size in selection sweeps analysis in pear (Wu et al. 2018; Zhang et al. 2021; Li et al. 2019), and apple (Duan et al. 2017). Therefore, compared to 100kb, here, we set 10 kb slide window more reasonable for the evaluation of selection sweeps region in pear.

Reference:

Wu, J., Wang, Y., Xu, J. et al. Diversification and independent domestication of Asian and European pears. Genome Biol **19**, 77 (2018). https://doi.org/10.1186/s13059-018-1452-y

Lam, HM., Xu, X., Liu, X. et al. Resequencing of 31 wild and cultivated soybean genomes identifies patterns of genetic diversity and selection. Nat Genet 42, 1053–1059 (2010). https://doi.org/10.1038/ng.715

Xu, X., Liu, X., Ge, S. et al. Resequencing 50 accessions of cultivated and wild rice yields markers for identifying agronomically important genes. Nat Biotechnol 30, 105–111 (2012). <https://doi.org/10.1038/nbt.2050>

Xiaolong Li, Lun Liu, Meiling Ming, Hongju Hu, Mingyue Zhang, Jing Fan, Bobo Song, Shaoling Zhang, Jun Wu, Comparative Transcriptomic Analysis Provides Insight into the Domestication and Improvement of Pear (P. pyrifolia) Fruit, Plant Physiology, Volume 180, Issue 1, May 2019, Pages 435–452, https://doi.org/10.1104/pp.18.01322

Zhang, MY., Xue, C., Hu, H. et al. Genome-wide association studies provide insights into the genetic determination of fruit traits of pear. Nat Commun 12, 1144 (2021). <https://doi.org/10.1038/s41467-021-21378-y>

Duan, N., Bai, Y., Sun, H. et al. Genome re-sequencing reveals the history of apple and supports a two-stage model for fruit enlargement. Nat Commun **8**, 249 (2017). https://doi.org/10.1038/s41467-017-00336-7

Cao, K., Zheng, Z., Wang, L. et al. Comparative population genomics reveals the domestication history of the peach, Prunus persica, and human influences on perennial fruit crops. Genome Biol 15, 415 (2014). <https://doi.org/10.1186/s13059-014-0415-1>

12. Line 465, additional details about the analysis of RNA-seq would be beneficial.

**Authors’ response:** Thanks for your suggestion, we have added more detail descriptions about “RNA-seq analysis” in revised manuscript. Please see the revision in lines 501-516.

13. Line 489, what is length of LD for filtering SNPs.

**Authors’ response:** Thanks for your suggestion. We used r^2^ to filter SNPs, and only independent SNPs (r^2^ < 0.25) were kept. Please see the revision in line 580.

Reviewer #2:

The manuscript perspective is interesting although addresses a good contribution to clarify the relationship of human collection and selection to early ripening trait in pear fruits and its correlation to DNA methylation patterns from the wild, landraces and improved cultivars. I recommend accepting this paper but with following minor revisions.

**Authors’ response:** We greatly appreciate the reviewer’s positive comments. We also thank the reviewer for all the constructive suggestions. In this edition, we had made corresponding analyses and revisions as suggested. Please see our point-by-point responses below.

1. 136-137, results indicate to down-regulation of DNA demethylase in pear ripening, while in tomato ripening was up-regulation of DNA demethylase genes (https://doi.org/10.1093/jxb/eraa003), while the authors used tomato to verify DNA methylation related genes in another part, may be compatibility or contradiction according to the fruits type. So, can the authors explain the variant in the discussion.

**Authors’ response:** Thanks for your nice suggestion. Actually, demethylase expression of tomato is increased during fruit ripening (Lang et al. 2017), while demethylase expression of pear is decreased during fruit ripening, indicating different methylation levels due to different species. In our study, the tomato was used to verify DNA methylation-related genes in pears; the reasons were as follows: Firstly, the tomato was often used as a model plant for fruit research (Quinet et al. 2019). The pear transformation system is especially difficult to accomplish, and it takes a long time to wait for fruit phenotyping after the transformation (Ni et al. 2020; Li et al. 2019; Yang et al. 2024). Secondly, in our experiment, the overexpression of the pear CAMTA2 gene in transgenic tomato plants exhibited a 500-fold higher expression level than that of WT (Additional file 2: Fig. S22) and showed ripening delaying in tomato fruit. All these results supported the idea that the CAMTA2 gene function in pears and tomatoes is similar. Although the DNA methylation pattern between tomato and pear during fruit ripening is different, the overexpression of CAMTA2 gene function in tomato is obvious; it indicated that there is little effect on the function. In this revision, we had added the explaination that the differences of DNA demethylase expression between pear ripening and tomato in discussion, and the reasons using tomato to verify methylation related CAMTA2 gene in pear. Please see the revision in line 427-439.

Reference:

Lang Z, Wang Y, Tang K, Tang D, Datsenka T, Cheng J, Zhang Y, Handa AK, Zhu J-K: Critical roles of DNA demethylation in the activation of ripening-induced genes and inhibition of ripening-repressed genes in tomato fruit. Proceedings of the National Academy of Sciences 2017, 114:E4511-E4519. https://doi.org/10.1073/pnas.1705233114

Quinet, M., Angosto, T., Yuste-Lisbona, F. J., Blanchard-Gros, R., Bigot, S., Martinez, J. P., & Lutts, S. (2019). Tomato Fruit Development and Metabolism. Frontiers in plant science, 10, 1554. <https://doi.org/10.3389/fpls.2019.01554>

Ni, J., Li, J., Zhu, R., Zhang, M., Qi, K., Zhang, S., & Wu, J. (2020). Overexpression of sugar transporter gene PbSWEET4 of pear causes sugar reduce and early senescence in leaves. Gene, 743, 144582. https://doi.org/10.1016/j.gene.2020.144582

Li M, Cheng C, Zhang X, Zhou S, Li L, Yang S. Overexpression of Pear (Pyrus pyrifolia) CAD2 in Tomato Affects Lignin Content. Molecules. 2019 Jul 17;24(14):2595. doi: 10.3390/molecules24142595. PMID: 31319487; PMCID: PMC6680972.

Yang, G., Sun, M., Brewer, L., Tang, Z., Nieuwenhuizen, N., Cooney, J., Xu, S., Sheng, J., Andre, C., Xue, C., Rebstock, R., Yang, B., Chang, W., Liu, Y., Li, J., Wang, R., Qin, M., Brendolise, C., Allan, A.C., Espley, R.V., Lin-Wang, K. and Wu, J. (2024), Allelic variation of BBX24 is a dominant determinant controlling red coloration and dwarfism in pear. Plant Biotechnol. J. <https://doi.org/10.1111/pbi.14280>

2. The concept of DNA methylation levels and genetic diversity need to be more considered because during the bisulfite treatment, insufficient conversion and DNA degradation can also result in a loss of sequencing diversity and specificity. (for more info <https://doi.org/10.1038/s41467-021-26278-9>)

**Authors’ response:** Sorry for our unclear description. Actually, we calculated the genetic diversity using SNPs data calling from whole genome resequencing (WGS) data, not bisulfite sequencing (BS-seq) data. Because WGS is often used to assess genetic diversity, which is more accurate than using BS-seq, as review’s comment that incomplete conversion and DNA degradation might result in the lack of specificity and the loss of sequencing diversity. Similar methods were also used in previous studies, including soybean (Shen et al. 2018) and tomato (Guo et al. 2023). In this revision, to avoid misunderstanding, we declare the method in detail. Please see the revision in line 568.

Reference:

Shen, Y., Zhang, J., Liu, Y. et al. DNA methylation footprints during soybean domestication and improvement. Genome Biol 19, 128 (2018). https://doi.org/10.1186/s13059-018-1516-z

Guo, H., Cao, P., Wang, C. et al. Population analysis reveals the roles of DNA methylation in tomato domestication and metabolic diversity. Sci. China Life Sci. 66, 1888–1902 (2023). https://doi.org/10.1007/s11427-022-2299-5

3. Is better if the authors select another scale color of figure (Fig.4a) to be clearer. While in Fig. 6 the color scale needs to be added.

**Authors’ response:** Thank you for your nice suggestion. We have selected another scale color of fig. 4a, and added the color scale in fig. 6a. Please see the corresponding revision in figures.

4. Line 360 - 363, DNA demethylation regulates, The authors need to support these information (See these article, it can be giving the authors more information "Regulation of Leaf Longevity by DML3-Mediated DNA Demethylation" https://doi.org/10.1016/j.molp.2020.06.006

**Authors’ response:** Thank you for the nice suggestion. We checked and studied the recommended reference, this research found that DNA demethylation regulates the expression of senescence-associated genes (SAGs) and further affect leaf senescence of Arabidopsis (Yuan et al. 2020). This is a good example that revealed the relationship between DNA methylation and leaf senescence. We have cited and added the relevant descriptions in discussion section. Please see lines 342-348.

Reference:

Yuan L, Wang D, Cao L, et al. Regulation of leaf longevity by DML3-mediated DNA demethylation[J]. Molecular plant, 2020, 13(8): 1149-1161. https://doi.org/10.1016/j.molp.2020.06.006

5. Line 388-391: The comparison between prior research about the impact of DNA methylation on gene expression is confusing, so how could you refer to this as a contradiction? Furthermore, the concluding comment distracts the reader, therefore it is recommended that it be reconsidered.

**Authors’ response:** Thank you for the nice suggestion. We agree with your suggestion, according to the comparison with previous research, we only can draw the conclusion that the relationship between the location of DMRs and expression level of their nearby gene is complicated. So, to avoid distracts the reader, we deleted the corresponding description.

6. There is some writing deficit so this manuscript should undergo prof-language editing before it can be published.

**Authors’ response:** We are so sorry for some mistake in English writing. In this revision, we asked for the help from a native English speaker colleague to polish the language. Wishing the English writing of this revision has been greatly improved and arrived the publication quality requirement for the journal.

Second round of review

Reviewer 1

The authors have fully addressed my concerns in the revised manuscript.

Reviewer 2

I have carefully reviewed the revised manuscript titled "Increased DNA methylation contributed to early ripening of pear fruits during domestication and improvement" and would like to express my appreciation for the thoughtful revisions made in response to the previous feedback. It is evident that the feedback provided during the initial review process has been thoroughly considered and addressed. The revisions reflect a commendable effort to improve the scientific rigor and overall quality of the manuscript significantly. I am pleased to recommend the acceptance of the revised manuscript for publication in Genome Biology.

Thank you for the opportunity to review this manuscript, and I look forward to seeing it published.

Authors Response

Point-by-point responses to the reviewers’ comments:

Reviewer #1:

The authors have fully addressed my concerns in the revised manuscript.

Authors’ response: Thanks for your valuable comment for the manuscript revision.

Reviewer #2:

I have carefully reviewed the revised manuscript titled "Increased DNA methylation contributed to early ripening of pear fruits during domestication and improvement" and would like to express my appreciation for the thoughtful revisions made in response to the previous feedback. It is evident that the feedback provided during the initial review process has been thoroughly considered and addressed. The revisions reflect a commendable effort to improve the scientific rigor and overall quality of the manuscript significantly. I am pleased to recommend the acceptance of the revised manuscript for publication in Genome Biology. Thank you for the opportunity to review this manuscript, and I look forward to seeing it published.

Authors’ response: Thanks for your valuable comment for the manuscript revision.
